# Neuroserpin gene therapy inhibits retinal ganglion cell apoptosis and promotes functional preservation in glaucoma

**DOI:** 10.1016/j.ymthe.2023.03.008

**Published:** 2023-03-11

**Authors:** Nitin Chitranshi, Rashi Rajput, Angela Godinez, Kanishka Pushpitha, Mehdi Mirzaei, Devaraj Basavarajappa, Veer Gupta, Samridhi Sharma, Yuyi You, Giovanna Galliciotti, Ghasem H. Salekdeh, Mark S. Baker, Stuart L. Graham, Vivek K. Gupta

**Affiliations:** 1 Faculty of Medicine and Health Sciences, Macquarie University, Sydney, NSW, Australia; 2 School of Medicine, Deakin University, Melbourne, VIC, Australia; 3 Institute of Neuropathology, University Medical Center Hamburg-Eppendorf, Hamburg, Germany; 4 School of Natural Sciences, Macquarie University, Macquarie Park, NSW 2109, Australia

**Keywords:** neuroserpin, plasminogen, tissue plasminogen activator, glaucoma, retinal ganglion cells, neurodegeneration

## Abstract

Our research has proven that the inhibitory activity of the serine protease inhibitor neuroserpin (NS) is impaired because of its oxidation deactivation in glaucoma. Using genetic NS knockout (*NS*^−/−^) and NS overexpression (*NS*^+/+ Tg^) animal models and antibody-based neutralization approaches, we demonstrate that NS loss is detrimental to retinal structure and function. NS ablation was associated with perturbations in autophagy and microglial and synaptic markers, leading to significantly enhanced IBA1, PSD95, beclin-1, and LC3-II/LC3-I ratio and reduced phosphorylated neurofilament heavy chain (pNFH) levels. On the other hand, NS upregulation promoted retinal ganglion cell (RGC) survival in wild-type and *NS*^−/−^ glaucomatous mice and increased pNFH expression. *NS*^+/+Tg^ mice demonstrated decreased PSD95, beclin-1, LC3-II/LC3-I ratio, and IBA1 following glaucoma induction, highlighting its protective role. We generated a novel reactive site NS variant (M^363^R-NS) resistant to oxidative deactivation. Intravitreal administration of M^363^R-NS was observed to rescue the RGC degenerative phenotype in *NS*^−/−^ mice. These findings demonstrate that NS dysfunction plays a key role in the glaucoma inner retinal degenerative phenotype and that modulating NS imparts significant protection to the retina. NS upregulation protected RGC function and restored biochemical networks associated with autophagy and microglial and synaptic function in glaucoma.

## Graphical abstract

**Figure:**
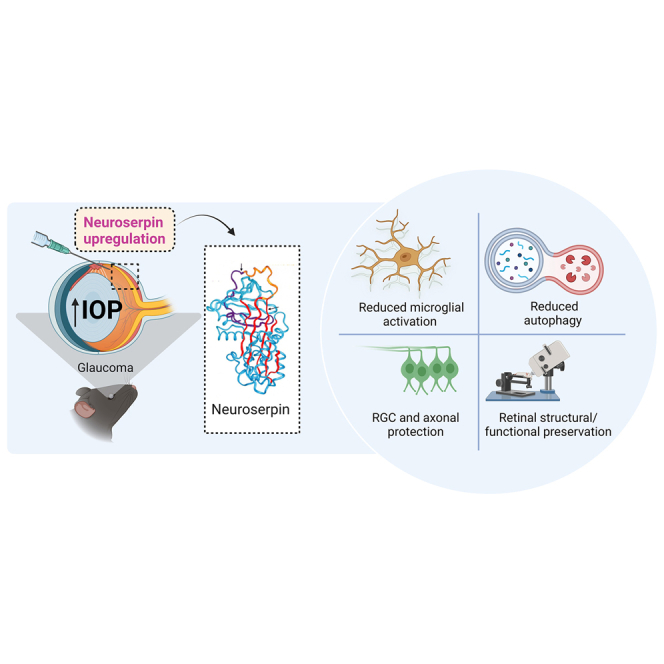

## Teaser

Targeting neuroserpin, a serine protease inhibitor in the retina, is protective against glaucoma damage in animals. An adeno-associated virus (AAV) gene therapy-based method for the overexpression of neuroserpin or administration of modified M^363^R neuroserpin, which is resistant to oxidation, provided retinal ganglion cell and optic nerve protection in glaucoma.

## Introduction

Glaucoma is the leading cause of irreversible vision loss, affecting more than 70 million people worldwide.^1^ Glaucoma is typically characterized by chronic injury to retinal ganglion cells (RGCs) and the optic nerve (ON). While increased intraocular pressure (IOP) has been shown to be a major risk factor, current treatment strategies are unable to prevent disease progression and subsequent vision loss in most patients.^2^^,^^3^ Several factors have been suggested to play a role in glaucoma pathology, such as remodeling of the lamina cribrosa, retrograde obstruction of neurotrophins, axonal compression of RGCs, chronic ischemic insult, and/or degradation of the extracellular matrix (ECM).^4^^,^^5^^,^^6^ Hence, enhancing our understanding of the molecular mechanisms of RGC and ON degeneration in glaucoma and preventing progression remains a priority.

Under physiological conditions, the zymogen pro-enzyme plasminogen (plg) is activated by tissue-type plg activator (tPA) and urokinase-type plg activator (uPA) to generate the broad-spectrum active serine protease plasmin.^7^ Within the nervous system, the activity of tPA is strongly modulated by the serine protease inhibitor neuroserpin (NS) encoded by the *SERPINI1* gene.^8^^,^^9^^,^^10^ NS is typically altered and expressed in the synaptic connections of the developing visual system of the mouse during activity-dependent remodeling processes.^11^ However, NS inhibitory activity against tPA/uPA is markedly diminished in various chronic neurodegenerative conditions and in acute-onset ischemic cerebral stroke.^12^ Moreover, reduced NS inhibitory activity results in aggravated ischemic brain injury and poor neurological outcomes.^13^ tPA activity is significantly increased and associated with exacerbated neuronal death and larger stroke infarct volume *in vivo*.^12^^,^^14^^,^^15^ However, recent animal model studies of retinal ischemic/reperfusion in glaucoma demonstrate the neuroprotective role of NS. This is proposed to occur through inhibition of intrinsic cell death signaling pathways mediated by caspase-3 and caspase-9, independent of any canonical inhibition with tPA/uPA.^16^

Accumulation of misfolded proteins within neurons is a hallmark of various chronic neurogenerative pathologies, where mutations of NS protein have been documented.^17^^,^^18^ The pathology of dementia familial encephalopathy with NS inclusion bodies (FENIB) is characterized by formation of mutant NS polymers, which accumulate within the neuronal endoplasmic reticulum (ER), resulting in enhanced ER stress.^19^ Further, ER-associated degradation (ERAD) is modulated by the ligases Hrd1 and gp78, which allow ubiquitination and degradation of mutant NS. Equally, loss of Hrd1 and gp78 activities increases the stability of mutant NS polymers.^20^^,^^21^ Accumulation of polymeric NS during disease progression induces an ER overload response, subsequently activating nuclear factor κB (NF-κB) signaling, which ultimately leads to increased neuronal apoptosis.^19^ Retention of NS polymers is also directly correlated with onset of dementia, cognitive decline, and epilepsy.^22^^,^^23^^,^^24^ Importantly, the mechanisms causing accumulation or (conversely) reduced NS clearance are poorly understood.

There is increasing evidence that tPA is involved in learning/memory in the hippocampus,^25^^,^^26^ fear/anxiety in the amygdala,^27^^,^^28^ autonomic and endocrine functions in the hypothalamus, and motor learning in the cerebellum.^29^^,^^30^ Detailed studies of Thy1cNS transgenic and NS knockout mice found increased phobic and anxiety-like responses, suggesting involvement in other brain functions involving behavior and emotions.^31^ Co-expression of NS in these regions implies roles in modifying elements of the tPA/plg cascade processes. In the adult brain, co-expression of tPA and NS reflects some role of NS in synaptic plasticity.^32^ Neuronal depolarization enhances transcription of NS, implicating it as an activity regulator of tPA activity and downstream proteolytic process within synapses.^33^ Further, the RNA binding protein HuD is co-expressed with NS mRNA in rat neurons and binds with high affinity to three AU-rich sequences in the 3′ UTR of NS mRNA. Overexpression of HuD leads to accumulation of NS mRNA and protein in rat PC12 cells.^34^ NS expression is also increased during neuroendocrine cell activation in *Xenopus* melanotrope cells.^35^

We have demonstrated previously that NS -plasmin interactions are increased in glaucoma and that NS’s plasmin-inhibitory activity is diminished. This loss of serpin-inhibitory activity was associated with enhanced methionine sulfoxide reactivity (presumably at the serpin active site) of NS under glaucomatous stress.^36^ In the current study, we expand that observation to investigate the neuroprotective role of NS in experimental glaucoma models using *NS*^−/−^ and *NS*^+/+Tg^ mice. Our results provide novel data suggesting that overexpression of NS in glaucoma imparts structural and functional protection to the retina, specifically by downregulating expression of the key postsynaptic scaffold protein PSD-95. Overexpression of NS in experimental glaucoma also reduced autophagy responses, where administration of NS protein or gene therapy promoted increased plasmin-inhibitory activity. Further, oxidative inactivation of NS by H_2_O_2_ (known to induce oxidative stress in SH-SY5Y cells) was overcome *in vivo* via administration of a novel, non-oxidizable, reactive-site-modified NS (M^363^R-NS). Administration of M^363^R-NS led to greater neurite outgrowth in SH-SY5Y cells and promoted neuroprotection of RGCs and ON axons in experimental animal glaucoma models. We also show that *in vivo* administration of oxidatively resistant M^363^R-NS provided increased plasmin-inhibitory activity and increased synaptophysin immunostaining in the glaucomatous retina.

## Results

### NS deficiency induces age-dependent degenerative changes in the retina

**Figure 1:**
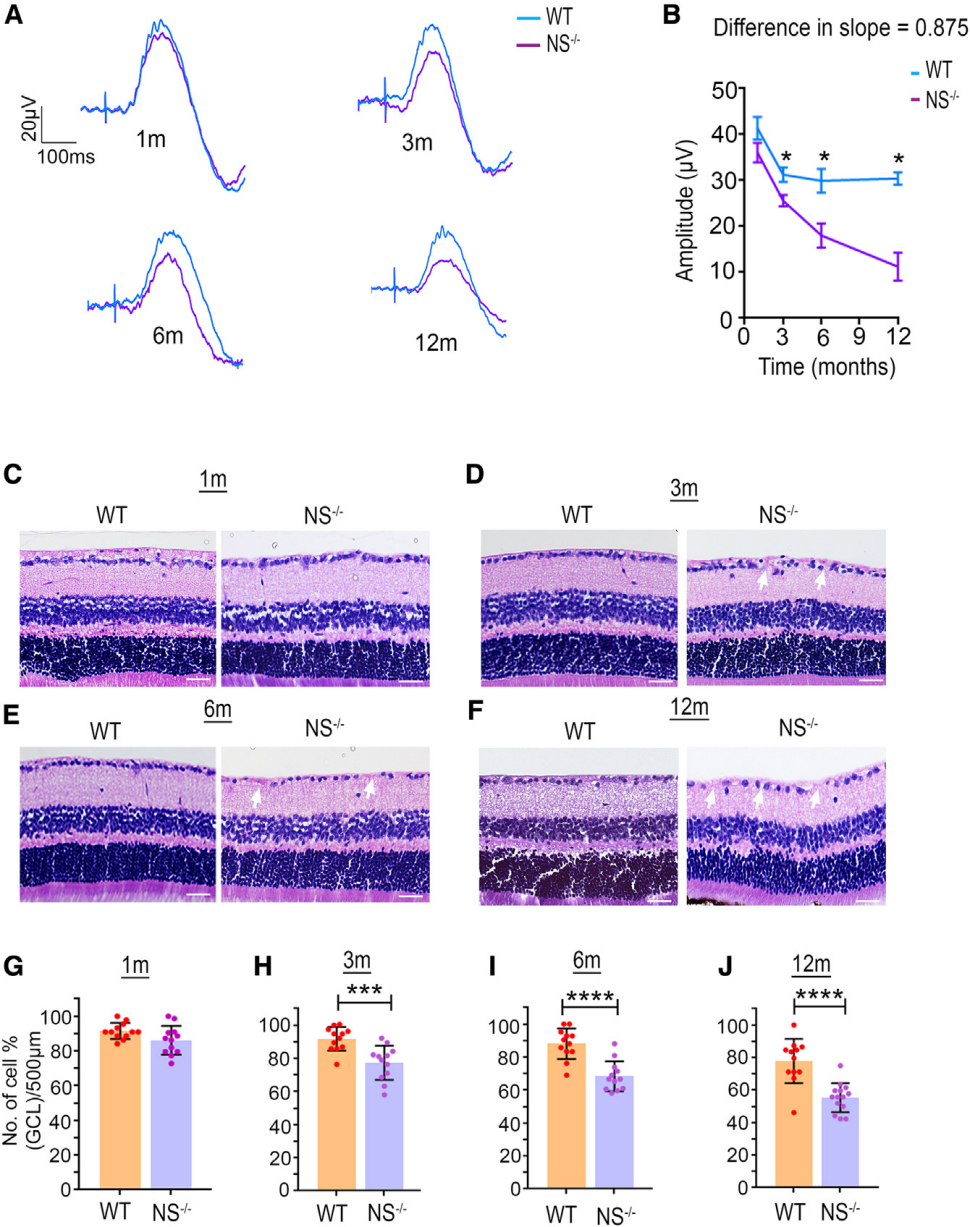
NS knockout mice show exacerbated retinal age-related degenerative changes Retinal function and structural damage in *NS*^−/−^ mice start early at 3 months of age. (A) Average trace of pSTR signal obtained from WT (blue) and *NS*^−/−^ mice (purple) obtained at different ages: 1 month, 3 months, 6 months, and 12 months. (B) Slope analysis for the pSTR functional parameter. A significant difference in the slope of decline was only observed in pSTR, suggesting that this parameters is exacerbated in the *NS*^−/−^ mouse group starting from 3 months and continued to 1 year (n = 10 animals/group, at time point). (C–F) Hematoxylin and eosin (H&E) staining of retinal sections, indicating changes in GCL density in WT and *NS*^−/−^ mice at four different age points: (C) 1 month, (D) 3 months, (E) 6 months, and (F) 12 months. Scale bars, 50 μm. (G) Quantification (H&E) indicating no differences observed in GCL density in *NS*^−/−^ mice at 1 month of age (n = 4 animals, 3 sections/animal in each group). (H–J) Quantification (H&E) indicating significant differences in GCL density in *NS*^−/−^ mice at 3, 6, and 12 months of age compared with age-matched WT mice (n = 4 animals, 3 sections/animal in each group; p < 0.005, p < 0.0001, p < 0.0001).

This study used wild-type (WT) and *NS*^−/−^ mice to examine the possibility that neurodegenerative changes to the retina could be induced by NS loss in an age-dependent manner, including resultant impacts on retinal structure and function. Retinal laminar structural changes were evaluated using H&E staining, while functional changes were analyzed using electroretinography (ERG) and positive scotopic threshold response (pSTR) electrophysiological recordings. Overall, inner retinal responses measured through pSTR amplitudes did not change significantly at 1 month of age, and significant progressive deficits were observed at 3 (p < 0.0001), 6 (p < 0.0002), and 12 (p < 0.0001) months of age. A loss of ∼26% was observed in WT mice at 1–12 months, while a more significant loss (∼64%) was observed in *NS*^−/−^ mice (Figures 1A and 1B). Whole retinal function measured through full-field scotopic ERGs further demonstrated no significant changes in waveform or amplitudes in the *NS*^−/−^ or *NS*^+/+Tg^ group, although a slight reduction in a- and b-wave amplitudes were observed in 6- and 12-month-old NS -ablated mice (Figure S1). Histochemical analysis of the retinal sections revealed that, while ganglion cell layer (GCL) density was unchanged at 1 month, and a progressive decline was seen at later ages, including 3 (p < 0.005), 6 (p < 0.0001), and 12 (p < 0.0001) months of age. WT mice demonstrated an ∼19% age-dependent decline over 1 year, while an ∼37% loss of GCL density was observed in *NS*^−/−^ mice (Figures 1C–1J). These histological findings were corroborated by optical coherence tomography OCT imaging results showing reduced GCL+IPL (inner plexiform layer) and whole retinal thickness in *NS*^−/−^ mice at 6 (p < 0.003) and 12 (p < 0.0001) months compared with WT mice (Figure S2). Loss of NS expression was established by densitometric quantification of western blot (WB) and immunofluorescence (IF) analysis of retinal sections at 3 and 12 months (Figure S3).

In contrast to *NS*^−/−^ mice, NS-overexpressing *NS*^+/+ Tg^ mice showed preservation of pSTR amplitudes with age compared with WT animals. At 1 and 3 months, no significant differences in pSTR amplitudes were evident; however, pSTR measurements at 6 (p < 0.0002) and 12 months (p < 0.0001) showed a progressive decline in amplitudes that was steeper in WT mice compared with NS^+/+ Tg^ mice (Figures S4A and S4B). H&E staining of retinal cross-sections revealed that GCL density was unaffected up to 3 months of age. At the 6-month (p = 0.003) and 12-month (p < 0.04) time points, a significantly greater age-dependent decline in GCL density was observed in WT mice compared with *NS*^+/+ Tg^ mice (Figures S4C–S4H). IF analyses revealed increased NS immunoreactivity in *NS*^+/+ Tg^ mouse retinal sections compared with WT mice. In addition, significantly increased NS levels in GCL were evident when normalized against co-staining of the RGC marker βIII-tubulin in *NS*^+/+ Tg^ mice at 3 (p < 0.0002) and 12 (p < 0.0001) months (Figures S5A–S5D). WB of retinal lysates and band density quantification data further confirmed the observation of higher NS levels at 3 (p < 0.007) and 12 (p < 0.003) months compared with WT mice (Figures S5E and S5F).

ON axonal staining in *NS*^−/−^ and *NS*^+/+ Tg^ mice supported retinal functional and morphometric observations with confirmation of insignificant differences at 1 month in the *NS*^−/−^ or *NS*^+/+ Tg^ group (Figures S6A and S6B) and progressive decline of axonal density in the *NS*^−/−^ group at 3 (p < 0.03), 6 (p < 0.009), and 12 (p < 0.0008) months compared with WT mice. In contrast, axonal density in *NS*^+/+ Tg^ mice was relatively preserved at 6 (p < 0.04) and 12 (p < 0.003) months compared with WT animals (Figures S6C–S6H). Histochemical analysis of ONs also showed altered pNFH levels that were diminished in *NS*^−/−^ at 3 (p < 0.0001) and 12 (p < 0.0001) months, suggesting neurodegenerative pathological changes with elevated levels of pNFH expression in *NS*^+/+ Tg^ mice at similar time points (p < 0.0001 [3 months]; p < 0.0001 [12 months]) (Figures S7A–S7D). On the other hand, expression of the inflammatory marker IBA1 in the ON was relatively increased in *NS*^−/−^ mice at 3 (p < 0.0001) and 12 (p < 0.0001) months and decreased in *NS*^+/+ Tg^ mice at 12 months (p < 0.007) (Figures S7E–S7H).

Because NS regulates synaptic function and axonal arborization, we analyzed potential alterations in synaptophysin and PSD95 expression as pre- and post-synaptic retinal markers, respectively. WB analysis revealed that synaptophysin expression was relatively unaffected in NS-ablated or -overexpression strains (Figures S8A–S8D). In contrast, PSD95 levels were elevated by ∼2-fold at 3 (p < 0.001) and 12 (p < 0.009) months in *NS*^−/−^ retinas, while no changes were evident in *NS*^+/+ Tg^ mice (Figures S8E–S8H). There was no change in autophagy responses in *NS*^−/−^ or *NS*^+/+ Tg^ mice at 3 or 12 months of age compared with age-matched WT controls (Figure S9).

### NS ablation exacerbates glaucoma deficits

**Figure 2:**
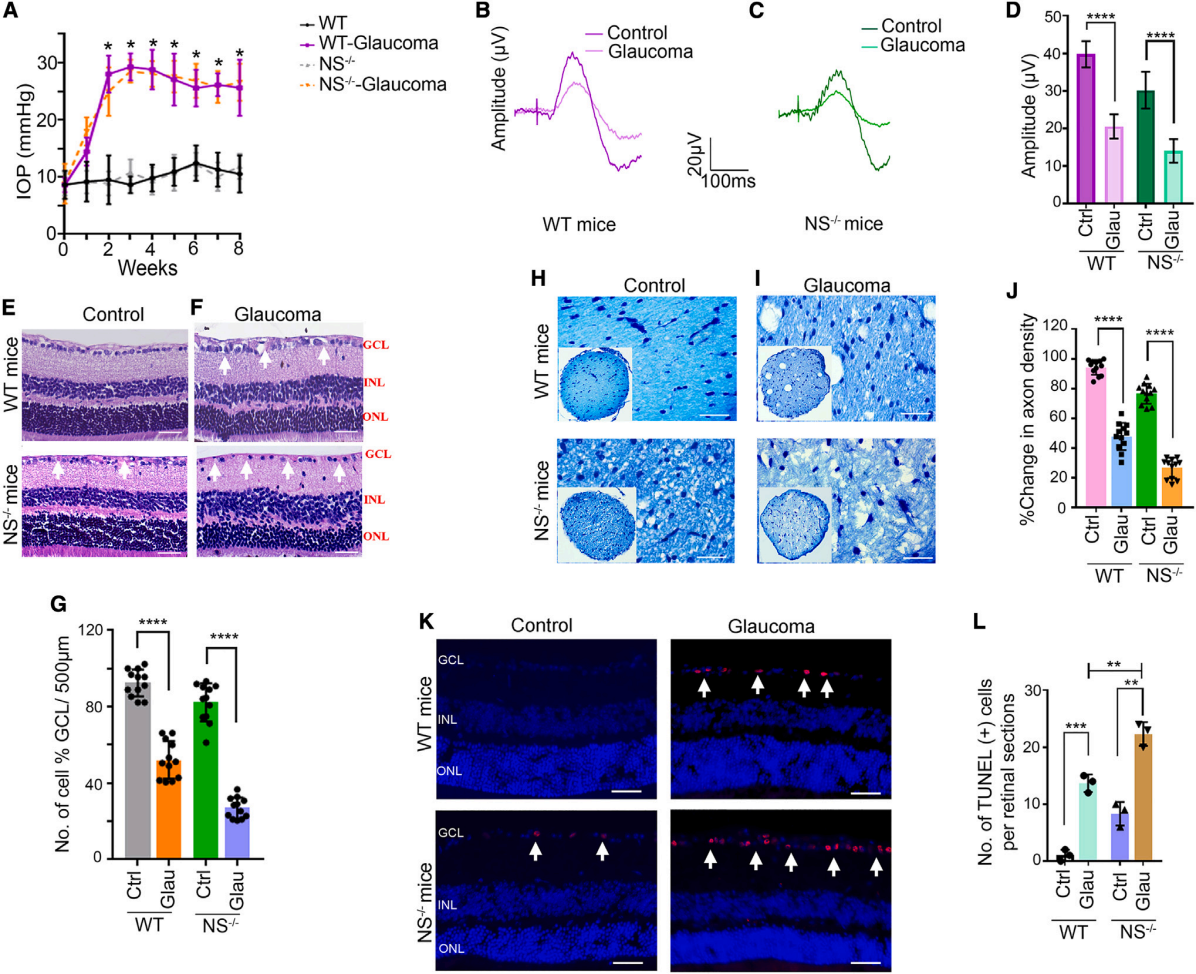
NS deficiency aggravates glaucomatous degenerative changes in the retina and ON Induction of chronic glaucoma causes inner retinal function and structure damage in WT and *NS*^−/−^ mice. (A) Non-injected mice (control) had a steady IOP level that was maintained at an average value of 11.6 ± 2.1 mm Hg (WT mice) and 11.4 ± 1.9 mm Hg (*NS*^−/−^ mice) throughout the period (n = 10 in each group). Weekly injections of microbeads induced an elevation of IOP and were maintained for 8 weeks (n = 10 in each group). Values are mean ± SEM. (B) pSTR traces in a normal (dark magenta) and chronic glaucoma (pink) model of WT retinas. (C) pSTR traces in a normal (dark green) and chronic glaucoma (cyan) model of *NS*^−/−^ retinas. (D) Significant differences in pSTR amplitudes were observed in WT mice exposed to chronically elevated IOP (n = 10 animals in each group, p < 0.001), and *NS*^−/−^ mice are more susceptible to glaucoma damage compared with WT counterparts (n = 10 animals in each group, p < 0.009 and p < 0.02). (E and F) Histological analysis of paraffin-embedded retinal sections from WT and *NS*^−/−^ mice stained by H&E under control and glaucoma conditions. Arrows indicate GCL. Scale bars, 50 μm). (G) There was a significant decrease in GCL cells in WT mice under chronic elevation of IOP (n = 4 animal, 3 sections/animal, p < 0.0001). The decrease in GCL density was significantly higher in *NS*^−/−^ mice under normal and glaucoma conditions (n = 4 animals, 3 sections/animal; p < 0.0001 and p < 0.004). (H and I) ON axonal appearance in WT and *NS*^−/−^ mice under control and experimental glaucoma conditions, stained with TB (10× and 63× resolution). (J) Quantification of axonal density revealed significant axonal loss in WT mice exposed to experimental glaucoma (n = 4 animal, 3 sections/animal, p < 0.0001). *NS*^−/−^ mice showed a significant lower axon density compared with WT mice, and experimental glaucoma further increases significant axon loss in these knockout mice (n = 4 animal, 3 sections/animal, p < 0.0001). (K) Increased TUNEL^+^ staining (red) was observed in retinal sections exposed to microbead injections in WT and *NS*^−/−^ mice in the inner retinal layers (white arrows). Shown are DAPI-stained cell nuclei (blue). Scale bars, 50 μm. (L) Quantification of TUNEL^+^ cells showing significantly increased numbers in WT glaucoma (n = 3 animals in each group, p < 0.0008). *NS*^−/−^ mice showed a significant increase in TUNEL^+^ cells in a control and experimental glaucoma model (n = 3 animals in each group, p < 0.002, p < 0.004). Graphs show means ± SEM, and p values were obtained using Student’s t test.

Potential detrimental effects of NS loss in glaucoma was investigated by subjecting WT and *NS*^−/−^ mice to an established experimental glaucoma model.^37^^,^^38^ As expected, increased IOP was observed in WT and *NS*^−/−^ mice subjected to microbead injections (mean ± SEM; WT, 23.7 ± 2.39; *NS*^−/−^, 23.90 ± 2.15) measured at 8 weeks compared with control eyes (mean ± SEM; WT, 10.11 ± 0.43; NS^−/−^, 10.19 ± 0.43) (Figure 2A). pSTR measurements showed reduced amplitudes in eyes with high IOP (WT, p < 0.0001; NS^−/−^, p < 0.009), with a greater loss noted in *NS*^−/−^ mice compared with WT mice (p < 0.0001) (Figures 2B–2D). The full-field scotopic ERGs representing whole retinal function were relatively unaffected, suggesting that retinal changes were mainly localized to the inner retina (Figure S10). Histochemical examination of retinal sections confirmed thinning of the GCL in response to high IOP exposure in WT and *NS*^−/−^ mice (WT, p < 0.0001; NS^−/−^, p < 0.0001) (Figures 2E and 2F). GCL loss was, however, exacerbated in *NS*^−/−^ mice compared with the WT (p < 0.0001) (Figure 2G). Similar detrimental effects of NS ablation were observed in ON axonal density in response to chronic IOP exposure (WT, p < 0.05; *NS*^−/−^ p < 0.001), with a greater loss evident in *NS*^−/−^ mice compared with the WT (p < 0.0001) (Figures 2H–2J). Reduced GCL density in high-IOP eyes concomitant with increased RGC apoptosis during experimental glaucoma (WT, p < 0.001; *NS*^−/−^, p < 0.0001). Terminal deoxynucleotidyltransferase-mediated dUTP nick end labeling (TUNEL)^+^ cells were increased greatly in *NS*^−/−^ mice compared with WT mouse eyes (p < 0.0001) (Figures 2K and 2L). IBA1 staining of ON sections revealed a significant elevation in experimental glaucoma (WT, p < 0.001; *NS*^−/−^, p < 0.001), and a greater increase in IBA1 immunoreactivity was observed in *NS*^−/−^ mice compared with WT mice (p < 0.0001) (Figures S11A–S11C). In contrast, pNFH levels were diminished in glaucoma, with a 1.6 ± 0.501-fold decrease in WT mice compared with a 2.46 ± 0.23-fold decrease in *NS*^−/−^ mice. This suggests that ON axons in *NS*^−/−^ mice were more susceptible to glaucoma-induced damage (p < 0.0001) (Figures S11D–S11F). We also analyzed NS expression in the retina using WB and IF. NS retinal protein expression and localization remained relatively unaltered under experimental glaucoma conditions; however, the plasmin inhibitory activity (PIA) of NS, as assessed by gelatin gel zymography, was reduced significantly (p < 0.005), indicating that NS function was compromised under disease conditions (Figure S12).

### NS neutralization induces retinal damage under healthy and glaucomatous conditions

We further analyzed the effect of intravitreal anti-NS antibody administration on retinas from healthy and experimental glaucoma animals. Eyes were subjected to weekly injections of anti-NS for 8 weeks, with control eyes subjected to control non-specific immunoglobulin G (IgG) injections. After 2 months, the eyes were examined for morphometric changes using retinal-section H&E staining. Significant thinning of the GCL was observed in retinas subjected to anti-NS treatment (p < 0.001), and this loss was exacerbated (p < 0.0001) in animals that were subjected to experimental glaucoma (Figures S13A–S13D). No significant impact of IgG treatment was noticeable under any treatment condition (Figure S13B). Retinal sections stained with NeuN/NS/DAPI corroborated these observations and showed a significant decline in NS expression and NeuN^+^ cells following anti-NS treatment under control and glaucomatous conditions (Figures S14 and S15). pSTR measurements supported the retinal histological findings, showing a reduced amplitude (p < 0.001) in eyes subjected to treatment with the anti-NS antibody (Figures S13E and S13F). Under glaucoma conditions, anti-NS antibody-treated eyes demonstrated a further decline in amplitude (p < 0.0001), suggesting that NS is essential for preservation of retinal function under normal and glaucomatous conditions (Figures S13G–S13H). ON axonal staining with toluidine blue further revealed that anti-NS treatment resulted in a significant negative impact on axonal density (p < 0.001) (Figures S13I and S13J), and this loss was enhanced significantly under glaucomatous conditions (p < 0.0001) (Figures S13K and S13L). Treatment with IgG alone had no significant impact under normal or experimental glaucoma conditions.

**Figure 3:**
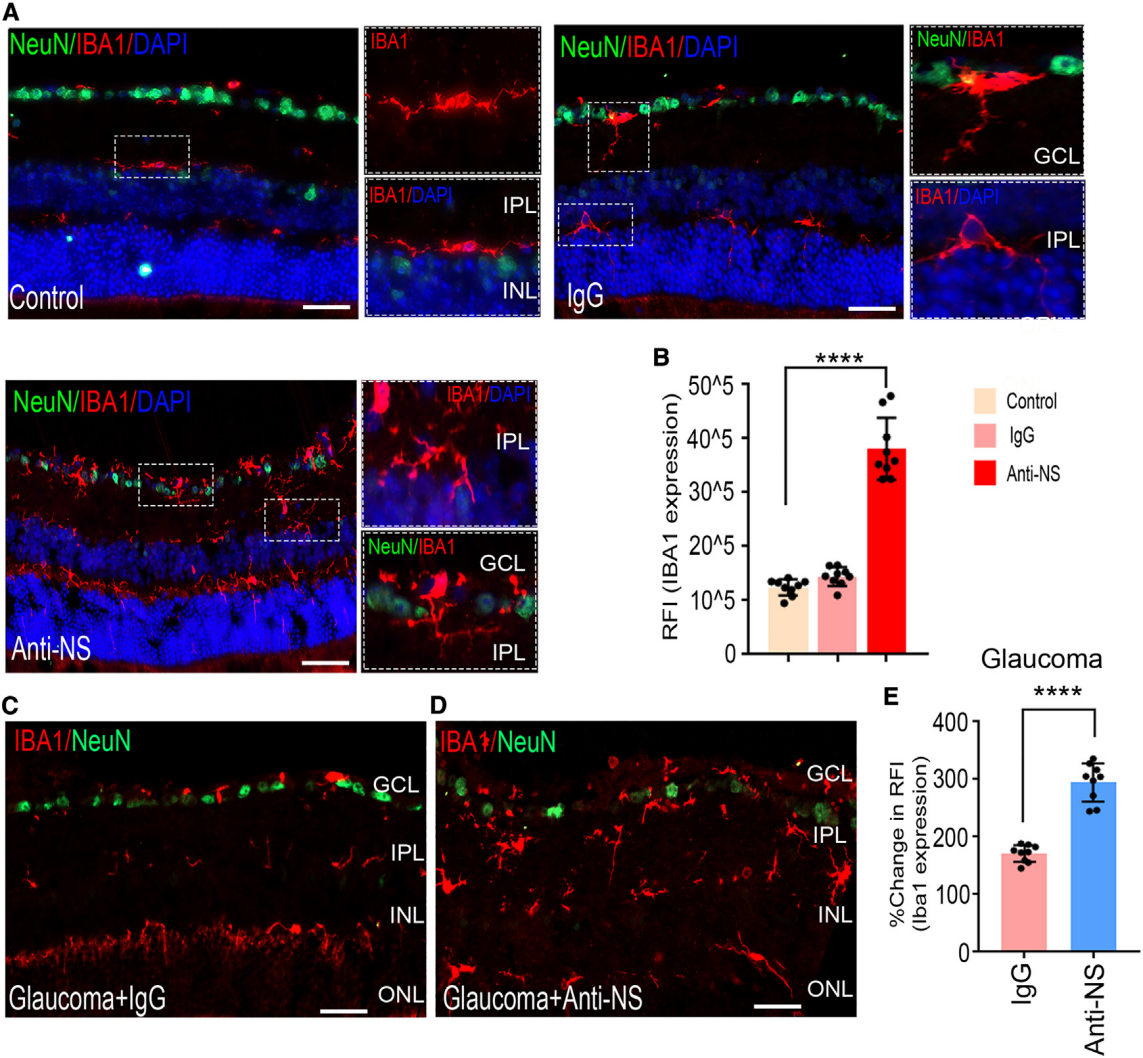
NS neutralization in the eyes induces microglial activation in healthy and glaucomatous animal retinas (A) Microglia analysis of retinal sections, showing representative photomicrographs indicating ionized calcium-binding adaptor molecule 1 (IBA1) immunostaining patterns in retinal cross-sections from control and IgG- and anti-NS-injected eyes of WT mice. Detailed images of microglia activation in the retina treated with anti-NS are shown in the right panel. (B) Analysis of IBA1 immunoreactivity following 8 weeks of IgG or anti-NS treatment showed a significant increase in IBA1 immunoreactivity in anti-NS-treated retinas (n = 3 animals, 3 sections/animal, p < 0.0001). (C and D) IBA1 immunostaining patterns in retinal cross-sections from glaucoma- and glaucoma + anti-NS-injected eyes. (E) Analysis of IBA1 immunoreactivity showed a significant increase in IBA1 immunoreactivity under high-IOP conditions at the 8-week time point compared with controls. IBA1 was significantly upregulated with anti-NS injections in experimental glaucoma. Scale bars, 50 μm). n = 3 animals, 3 sections/animal; p < 0.0001). IBA1, red; NeuN, green; DAPI, blue. Graphs show means ± SEM, and p values were obtained using Student’s t test.

We also evaluated retinal sections for evidence of apoptosis by TUNEL staining. The results supported histological findings and showed increased TUNEL^+^ cells mainly localized to inner retinal layers upon NS antibody treatment (p < 0.002) (Figures S13M and S13N). Further increased TUNEL^+^ staining was evident in glaucomatous retinas subjected to NS neutralization (p < 0.0003) (Figure S13O). Analysis of IBA1 showed significantly enhanced levels in anti-NS-treated retinas (Figures 3A and 3B) (p < 0.0009). IBA1 levels were enhanced in glaucoma and further exacerbated in glaucoma retinas subjected to NS neutralization (p < 0.0001) (Figures 3C–3E).

Effects of anti-NS administration on NS levels and PIA were assessed in the retina. WB (p < 0.0006) and IF (p < 0.0001) analysis demonstrated loss of NS immunoreactivity in the retina upon anti-NS treatment under control and experimental glaucoma conditions (Figures S14 and S16). The PIA of NS was also found to be significantly reduced in parallel with NS expression changes (p < 0.0007) (Figures S16A–S16D). A comparable increase in IOP was observed in high-IOP eyes in normal and anti-NS antibody-injected mice (Figure S17A). The average a- and b-wave scotopic ERG amplitudes were relatively unaffected in all groups, suggesting that anti-NS treatment preferentially affected only inner retinal layers (Figures S17B–S17G).

The impact of NS neutralization on autophagy was further examined in anti-NS-treated eyes. We observed significantly elevated levels of beclin-1 (control, p < 0.02; glaucoma, p < 0.03) and LC3BII/I markers (control, p < 0.03; glaucoma, p < 0.007) in retinas under control and experimental glaucoma conditions (Figures S18A–S18C). Equally, NS neutralization resulted in loss of pre-synaptic marker synaptophysin (p < 0.003) and resulted in upregulation of the postsynaptic marker PSD95 (p < 0.02) (Figures S18D–S18F), as observed in *NS*^−/−^ mice.

### *NS*^+/+ Tg^ animals are less susceptible to glaucoma damage

**Figure 4:**
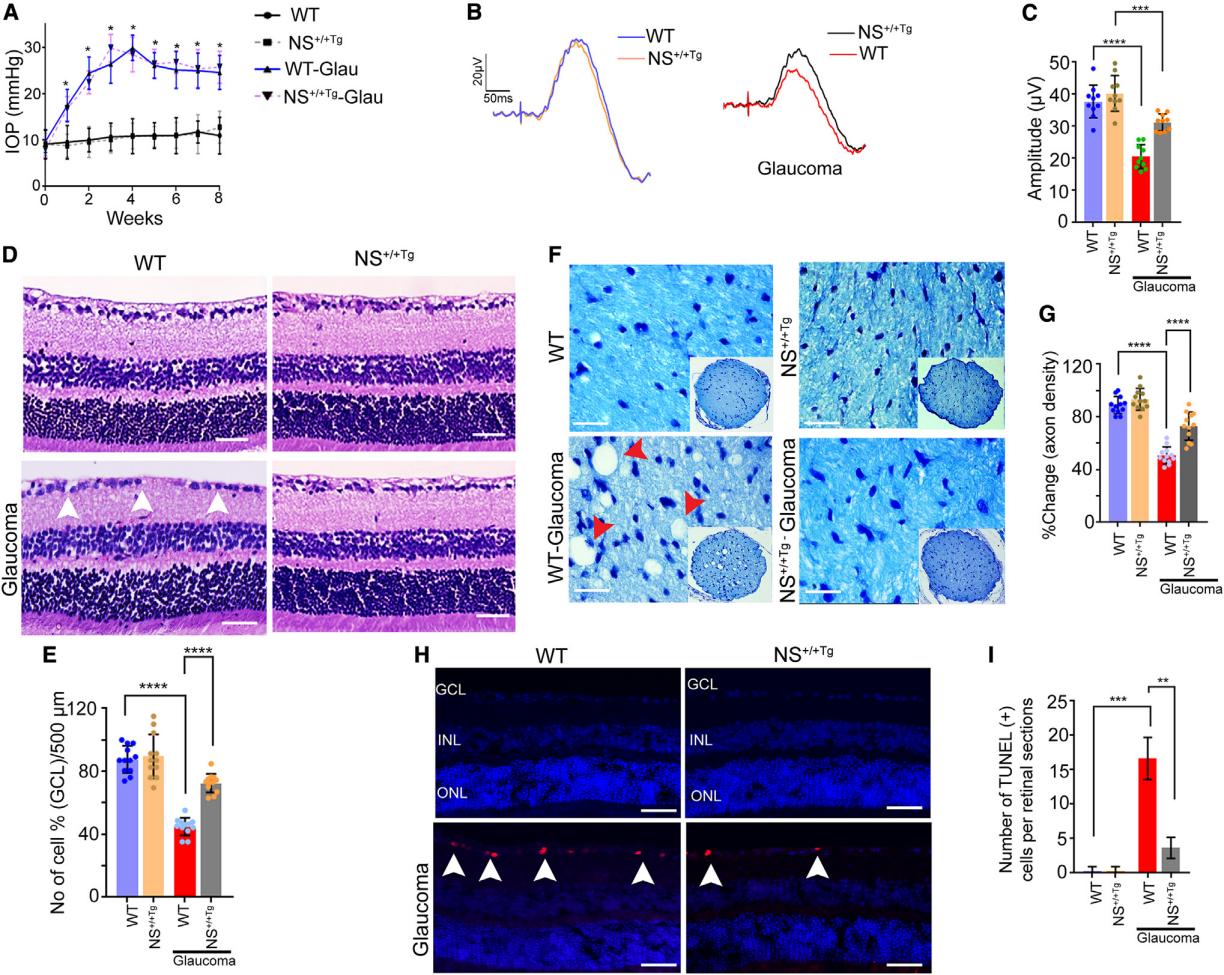
NS overexpression protects inner retinal function and structure in chronic glaucoma (A) Sustained IOP elevation in WT and *NS*^+/+ Tg^ animal eyes for 8 weeks following microbead injections. (B) pSTR responses in normal and chronic glaucoma models of WT (blue and red) and *NS*^+/+ Tg^ (orange and black) retinas. (C) No change in pSTR amplitude was observed in WT and *NS*^+/+ Tg^ mice under normal conditions. Significant differences were observed among the pSTR amplitudes of WT mice exposed to chronically elevated IOP (p < 0.0001), which are more susceptible to glaucomatous damage compared with their *NS*^+/+ Tg^ counterparts (p < 0.0001, n = 10 animals/group), which represents remarkable protection in inner retinal function under similar conditions. (D) Histological analysis of paraffin-embedded retinal sections from WT and *NS*^+/+ Tg^ stained by H&E. Arrows indicate GCL. Scale bars, 50 μm). (E) There was a significant decrease in GCL cells in WT mice under chronic elevation of IOP (p < 0.0001; n = 4 animals, 3 sections/animal) compared with *NS*^+/+ Tg^ mice (p < 0.0001; n = 6 animals in each group). (F) Cross-sections of WT and *NS*^+/+ Tg^ ON with or without high IOP counterstained with TB. Scale bars, 20 μm. (G) Quantification (TB) indicating a significant decline in axon density in WT compared with *NS*^+/+Tg^ mice under high IOP (n = 4 animals, 3 sections/animal; p < 0.0001 and p < 0.0001). (H) Increased TUNEL^+^ staining (red) was observed in WT retinal sections exposed to high IOP compared with *NS*^+/+ Tg^ mouse retinas under experimental glaucoma in the GCL (white arrows). Shown are DAPI-stained cell nuclei (blue). Scale bars, 50 μm), (I) Quantification of TUNEL^+^ cells showing significantly increased numbers in WT retinas exposed to high IOP compared with *NS*^+/+ Tg^ retinas (n = 3 animals in each group, p < 0.0003). Graphs show means ± SEM, and p values were obtained using Student’s t test.

We further sought to establish the protective role of NS by investigating *NS*^+/+ Tg^ mouse retinas using electrophysiological, histological, and biochemical analysis under control and glaucomatous conditions. WT and *NS*^+/+ Tg^ mice (6 weeks old) were subjected to experimental glaucoma conditions with comparable IOP observed in both groups under these conditions at 2 months (WT, 23.2 ± 2.00; *NS*^+/+ Tg^, 23.34 ± 2.29; WT microbeads, 10.55 ± 0.28 mm Hg; *NS*^+/+ Tg^ microbeads, 10.48 ± 0.41 mm Hg) (Figure 4A). pSTR measurements revealed comparable amplitudes between both groups. However, under glaucomatous conditions, *NS*^+/+ Tg^ mice were significantly protected from IOP injury compared with WT counterparts (p < 0.0001) (Figures 4B and 4C). The whole retinal scotopic a- and b-wave ERG amplitudes remained relatively unaltered in WT and *NS*^+/+ Tg^ mice under control and glaucoma conditions (Figures S19A–S19D). This protective effect was also corroborated histologically, where *NS*^+/+ Tg^ mice demonstrated significantly reduced GCL loss in the retina compared with the WT strain under increased IOP conditions (p < 0.0001) (Figures 4D and 4E). ON axonal staining confirmed these observations, showing that *NS*^+/+ Tg^ mice were relatively protected against ON injury in experimental glaucoma (p < 0.0001) (Figures 4F and 4G). No significant differences were observed between WT and *NS*^+/+ Tg^ mice under normal IOP conditions. Further, we investigated apoptosis pathway activation in *NS*^+/+ Tg^ mouse eyes by measuring retinal-section TUNEL staining (Figure 4H). Parallel to our histological findings, a significant enhancement in TUNEL^+^ cells was observed in WT mice with glaucoma, while increases were relatively modest in *NS*^+/+ Tg^ mice (p < 0.003) (Figures 4H and 4I). Analysis of retinal lysates established that *NS*^+/+ Tg^ expressed significantly elevated NS in control (p < 0.01) and glaucoma (p < 0.05) compared with WT c-ounterparts. Increased NS levels corresponded with increased PIA activity under control (p < 0.04) and glaucomatous conditions (p < 0.02) (Figures S20A–S20C). While PIA decreased significantly in WT mice exposed to high IOP (p < 0.009), declines were relatively modest in *NS*^+/+ Tg^ mouse retinas (p < 0.02) (Figures S20A and S20B). *NS*^+/+ Tg^ retinal examination with WB revealed enhanced levels of the synaptic marker synaptophysin (p < 0.04) with no change in PSD95 expression compared with WT mice under control conditions (Figure S21). However, when exposed to experimental glaucoma, synaptophysin showed an increase (p < 0.02) in *NS*^+/+ Tg^ mice, and PSD95 declined (p < 0.03), suggesting differential effects of NS expression change on pre- and post-synaptic biological processes (Figures S21A–S21D). Synaptophysin changes were further established using retinal IF staining (Figure S22), where *NS*^+/+ Tg^ mice demonstrated a differential effect on the autophagic markers beclin 1 and LC3B II/I ratio levels (Figure S23A). WB examination of retinal lysates revealed that, while beclin 1 expression was only affected under glaucoma conditions and was reduced compared with WT mice (p < 0.05), LC3BII/I was relatively reduced in *NS*^+/+ Tg^ retinas under control (p < 0.05) and experimental glaucoma conditions (p < 0.009; Figures S23A–S23C).

### Adeno-associated virus (AAV)-mediated NS overexpression imparts protection against glaucoma pathology

**Figure 5:**
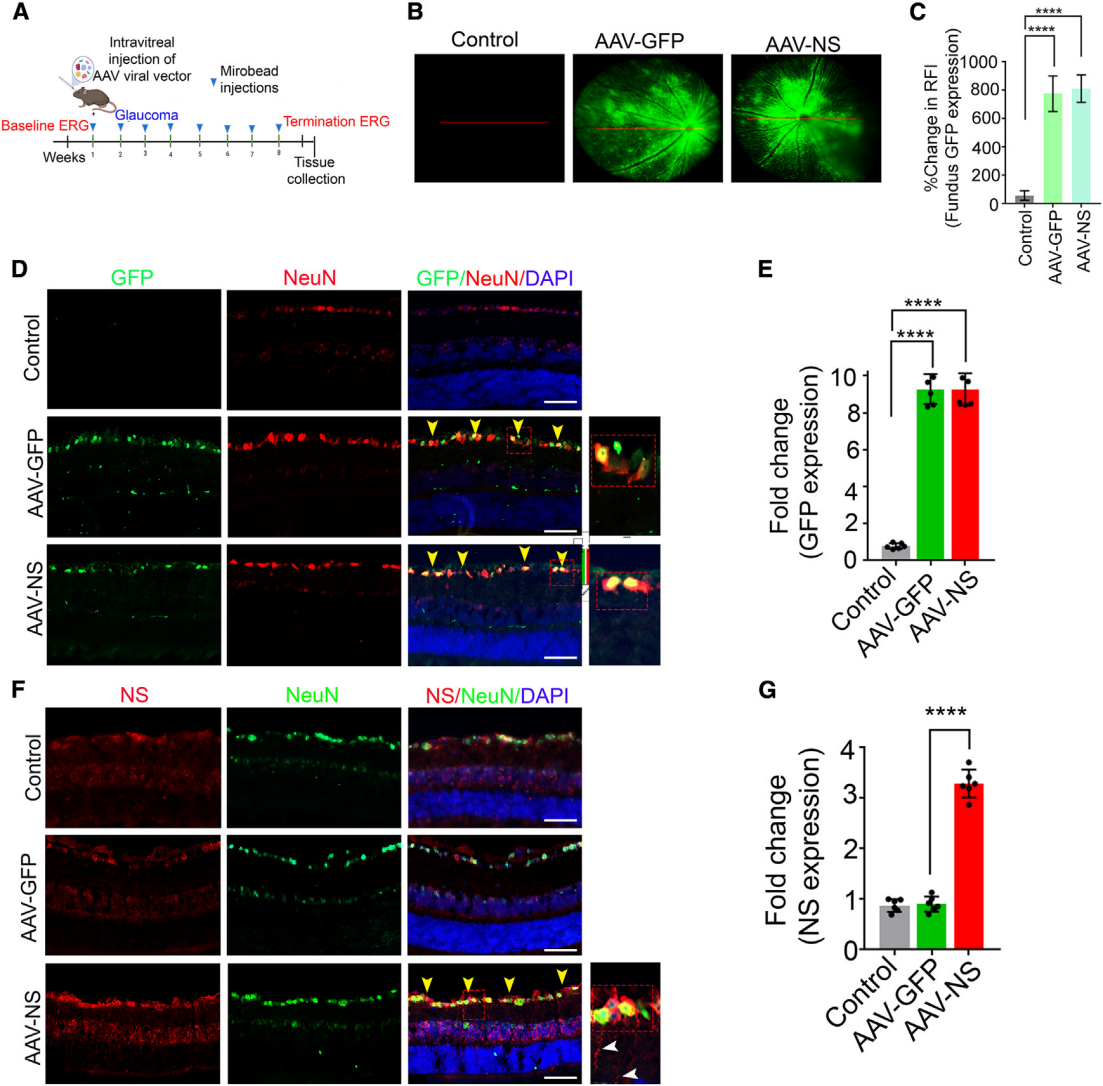
Adeno-associated virus serotype 2 (AAV-2)-mediated GFP and NS overexpression in ganglion cells (A) Schematic representation of the AAV administration experimental timeline. (B) Animal eyes were injected with AAV2 constructs for NS overexpression. GFP alone was used as a control. (C) Quantification of GFP fluorescence represented as the percentage of baseline GFP fluorescence. n = 9, p < 0.0001, paired Student’s t test. (D) Images of retinal sections from mice expressing EGFP (green) after 2 months of viral vector treatment. NeuN (red) was used to stain ganglion cells, and the yellow arrow shows colocalization of NeuN-GFP. A higher magnification of GCL is shown on the right. DAPI (blue) was used to stain nuclei. (E) Fold change in baseline GFP fluorescence. n = 4, p < 0.0001, paired Student’s t test. (F) Images of retinal sections from mice expressing NS (red) after 2 months of viral vector treatment. NeuN (green), colocalization of NeuN-NS (indicated by yellow arrowheads). A higher magnification of GCL is shown on the right. DAPI, blue. (G) Quantification of fluorescence intensity representing fold change in NS (n = 4, p < 0.0001, paired Student’s t test). Scale bars, 50 μm.

Having established that generalized NS overexpression was protective against injury during experimental glaucoma, we investigated whether targeting NS expression specifically to RGCs with intravitreal AAV administration protected the retina. The NS viral vectors containing human neuroserpin (hNS) sequence (AAV2-CAG2-EGFP-2A-hNS-woodchuck hepatitis virus posttranscriptional regulatory element (WPRE) or AAV2-NS; AAV2-CAG2-EGFP or AAV2-GFP) are represented in Figure S24A. Viral vector expression was initially validated by transducing SH-SY5Y cells with control, AAV-GFP, and AAV-NS vectors, followed by quantifying lysates for GFP and NS expression with antibodies (Figures S24B–S24F). Animals were subjected to AAV-GFP or AAV-NS administration, and expression was monitored using Micron IV fundus imaging in live animals at 2 months (p < 0.0001) (Figures 5A–5C) before animals were sacrificed and retinal sections were analyzed for GFP and NS expression. A 13% ± 4% increase in GFP expression was evident in AAV GFP and AAV-NS animals (p < 0.0001) (Figures 5D–5G). GFP immunoreactivity revealed a 12 ± 5-fold increase in AAV-GFP- and AAV-NS-administered animals (Figures 5D and 5E), and NS immunoreactivity revealed a 4 ± 2-fold increase in AAV-NS-administered animals compared with control and AAV-GFP-administered retinas (p < 0.0001) (Figures 5F and 5G). NeuN was used as a marker for ganglion cells (Figures 5D and 5F). WB analysis revealed significantly increased NS expression in AAV-NS treated retinas compared with control and AAV-GFP-treated mice (p < 0.01) (Figures S25A and S25C). Retinal tissues were subjected to PIA assays, and NS inhibitory activity was significantly elevated in AAV-NS-administered control IOP (p < 0.04) and glaucomatous mice (p < 0.001) compared with control AAV-GFP tissues (Figures S25A and S25B). Retinas subjected to high IOP for 2 months were examined for morphometric changes using H&E staining and inner retinal functional changes using pSTR and TUNEL apoptotic changes (Figure S26). The data revealed that GCL density in animals subjected to NS upregulation was significantly protected during glaucoma injury (p < 0.0001). However, no changes were observed when NS was upregulated under normal healthy conditions (Figures S26A–S26D). ON histochemical analysis using toluidine blue further revealed that axonal density was reduced under glaucomatous conditions while being significantly protected in mice administered AAV-NS (p < 0.003) (Figures S27A–S27C). Similarly, pSTR amplitudes were significantly protected in animals subjected to NS upregulation compared with control glaucomatous animals (p < 0.0004) (Figures S27E–S27H). These findings were supported by TUNEL staining observations, where reduced TUNEL apoptosis staining was observed in glaucomatous animals subjected to AAV-mediated NS upregulation in RGCs (p < 0.001) (Figures S26I and S26J). Biochemical analysis of ON sections demonstrated that pNFH levels were significantly reduced (p < 0.008) and IBA1 levels significantly (p < 0.0001) elevated under glaucoma conditions. pNFH immunoreactivity was relatively increased (p < 0.03) and IBA1 moderately decreased (p < 0.0001) in glaucomatous animals subjected to AAV NS treatment (Figures S27D–S27I). A comparable increase in IOP was observed in glaucoma-treated (22.44 ± 2.16), glaucoma + AAV-GFP-treated (23.65 ± 2.83), and glaucoma + AAV NS-treated (24.62 ± 2.35) mice compared with the control (10.68 ± 0.41 mm Hg) group (Figure S28A). No significant changes in global scotopic a- or b-wave amplitudes were observed in the glaucoma-, AAV-GFP-, or AAV-NS-treated groups (Figures S28B–S28E). WB analysis of the retinal tissues showed that the autophagy markers beclin 1 (p < 0.03) and *LC3B-II/I* (p < 0.03), which are enhanced under glaucoma conditions, were significantly decreased in the AAV-NS-treated glaucoma group (p < 0.007 and p < 0.005 respectively) (Figure S29). Pre-synaptic marker synaptophysin and post-synaptic marker PSD95 assessment showed their modulation in response to AAV-NS administration (Figure S30). Synaptophysin was decreased in the retina under glaucoma conditions (p < 0.005), while its levels were rescued in the group treated with AAV-NS (p < 0.006) (Figures S30A and S30B). Conversely, PSD95 levels were enhanced in glaucoma (p < 0.0004) and significantly reduced in response to AAV-NS treatment (p < 0.004) (Figures S30A and S30C).

### AAV NS gene therapy rescues RGC degeneration in glaucomatous *NS*^−/−^ mice

**Figure 6:**
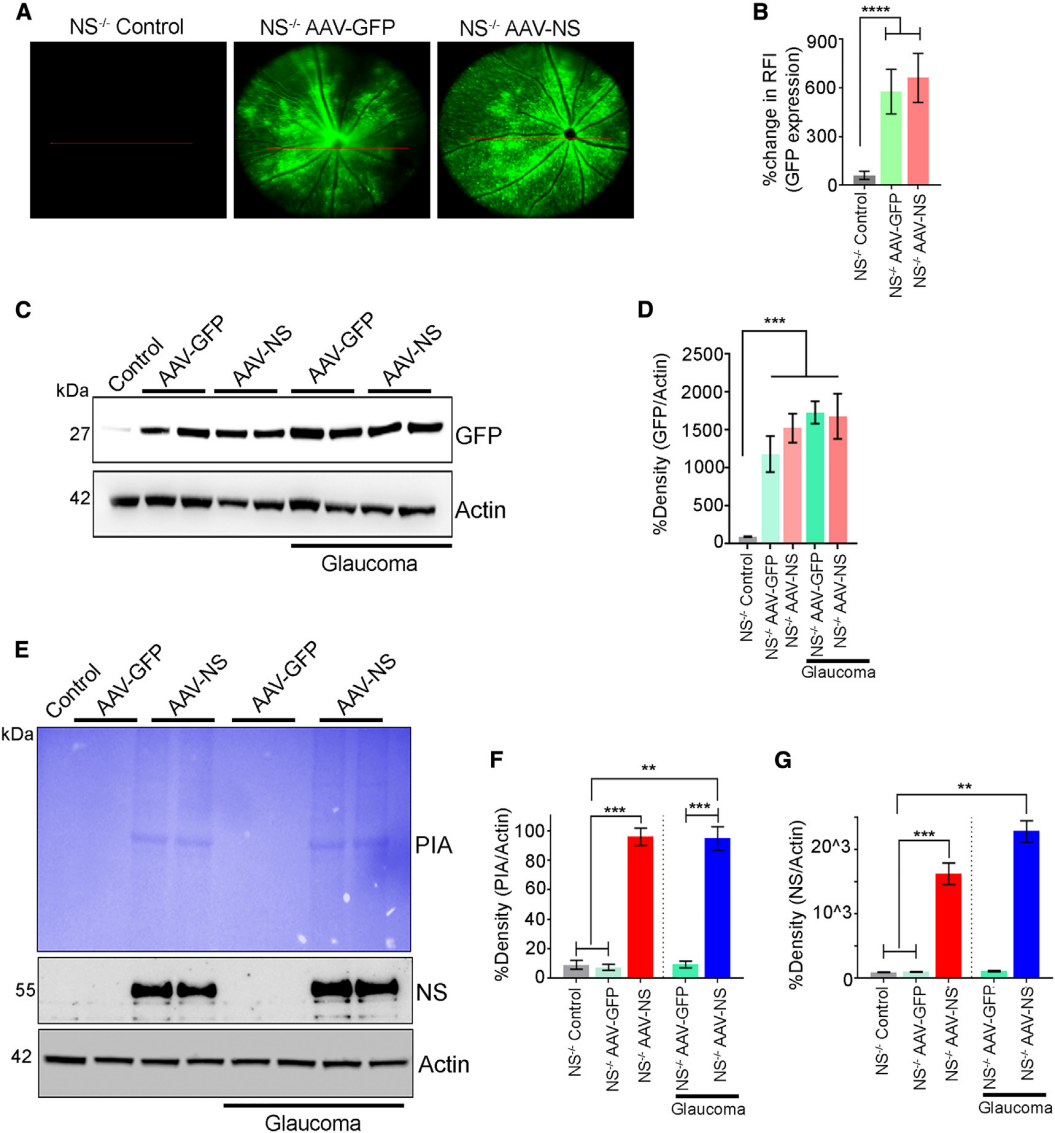
NS expression and plasmin inhibitory activity (PIA) in NS-ablated mice under control and glaucoma conditions (A) Fundus photographs showing GFP expression 2 months after intravitreal administration of AAV2 vectors in mouse retinas. (B) Quantification of the relative fluorescence intensity (p < 0.0001, n = 10 animals/group). (C) WB analysis of ONH lysates revealed expression of GFP in AAV-treated *NS*^−/−^ mouse retinas. (D) GFP fold changes relative to control *NS*^−/−^ mice retina under control and glaucoma conditions. Data are shown as mean ± SEM (n = 10 animals/group, p < 0.005, ANOVA). (E) ONH lysates from control and AAV-GFP- and AAV-NS-treated *NS*^−/−^ retinas under normal and high-IOP conditions were subjected to gelatin zymography to evaluate the PIA of NS. The blots were also probed for NS immunoreactivity in each case (n = 3 each). (F) Relative band intensities were quantified, and data analysis indicated that overexpression of NS in NS-ablated mice has significantly higher PIA under normal and glaucoma conditions (p < 0.003 and p < 0.005; n = 3 animals/group). (G) Significantly higher NS expression compared with control and AAV-GFP treatment under both control (p < 0.006) and glaucoma conditions was observed (p < 0.003, n = 3 animals/group).

To investigate whether AAV-NS gene therapy protected *NS*^−/−^ mice against inner retinal degeneration from experimental glaucoma, *NS*^−/−^ mice were subjected to AAV-GFP or AAV-NS administration, and expression was confirmed using Micron IV fundus imaging in live animals (p < 0.0001 at 2 months) (Figures 6A and 6B). *NS*^−/−^ mouse eyes were subjected to experimental chronic glaucoma, and IOP was observed in AAV treatment under control and glaucomatous conditions (8 weeks) (*NS*^−/−^, 10.11 ± 0.29 mm Hg; NS^−/−^ + AAV-GFP, 9.94 ± 0.31 mm Hg; NS^−/−^ + AAV-NS, 10.52 ± 0.36 mm Hg; NS^−/−^ microbeads, 24.13 ± 2.27 mm Hg; NS^−/−^ microbeads + AAV-GFP, 24.53 ± 2.40 mm Hg; NS^−/−^ microbeads + AAV-NS, 24.51 ± 2.46 mm Hg) (Figure S31A). Animals were sacrificed at 2 months, and retinal sections were analyzed for GFP expression. A 12- to 13-fold increase in GFP expression was evident in AAV-GFP and AAV-NS animals (p < 0.0001) (Figures 6A and 6B), and WB revealed a significant increase in GFP expression in AAV-GFP- and AAV-NS-treated retinas compared with non-treated control *NS*^−/−^ mouse samples (p < 0.005) (Figures 6C and 6D). Retinal tissues were also subjected to PIA assays, and NS activity was significantly elevated in AAV-NS-administered control IOP (p < 0.003) and glaucomatous mice (p < 0.005) compared with control tissues (Figures 6E–6G). *NS*^−/−^ mouse retinas were subjected to AAV-GFP and AAV-NS administration under healthy and high IOP conditions for 2 months and examined for inner retinal functional changes using pSTR, morphometric changes using H&E staining, and TUNEL apoptosis changes. Data revealed that pSTR amplitudes were significantly protected in animals subjected to NS upregulation compared with non-treated or AAV-GFP-treated *NS*^−/−^ mice in healthy and glaucomatous animals (p < 0.0008 and p < 0.0001) (Figures S32A–S32C). The whole retinal scotopic a- and b-wave ERG amplitudes remained relatively unaltered with AAV treatment under healthy and glaucomatous conditions (Figures S31B–S31G).

Correspondingly, GCL density in animals with NS upregulation was significantly higher under healthy conditions (p < 0.0001) compared with experimental glaucoma (p < 0.001). However, no changes were seen when GFP alone was upregulated (Figures S32D and S32E). These findings were supported by reduced TUNEL staining in normal healthy and glaucomatous *NS*^−/−^ animals with RGC NS upregulation (p < 0.0001) (Figures S32F and S32G). NeuN expression increased 1- to 3-fold in AAV-NS-administered animals compared with controls and AAV-GFP-administered *NS*^−/−^ mice retinas under normal and glaucomatous conditions (p < 0.0001) (Figures S33A–S33J). *NS*^−/−^ mouse ON histochemical analysis using toluidine blue further revealed that axonal density was reduced in glaucoma but significantly protected in AAV-NS mice under healthy and glaucomatous conditions (p < 0.002, p < 0.0003) (Figures S34A–S34C). Histochemical analysis of ON sections demonstrated that pNFH levels in *NS*^−/−^ mice were reduced with control and AAV-GFP treatment (p < 0.003) and significantly reduced (p < 0.0007) in glaucoma animals, but its levels were significantly increased in glaucomatous animals subjected to AAV-NS treatment (p < 0.0004) (Figures S34D–S34F). In contrast, IBA1 immunostaining in *NS*^−/−^ mice was higher in the control and AAV-GFP treatment groups (p < 0.0001) and significantly elevated under glaucoma conditions (p < 0.0001) while being decreased (p < 0.0001) in glaucomatous animals with AAV-NS treatment (Figures S34G–S34I).

WB analysis of retinal tissues showed that the autophagy markers beclin 1 (p < 0.03) and LC3B-II/I (p < 0.05) were enhanced under glaucomatous conditions and significantly decreased in AAV-NS-treated experimental glaucoma mice (p < 0.006 and p < 0.009, respectively) (Figures S35A–S35C). Pre-synaptic marker synaptophysin and post-synaptic marker PSD95 assessment showed their modulation in response to AAV-NS administration. Synaptophysin was decreased in the retina under glaucomatous conditions (p < 0.04), while its levels were rescued in AAV-NS mice (p < 0.02). Conversely, PSD95 levels were enhanced in glaucoma (p < 0.03) but significantly suppressed in response to AAV-NS (p < 0.02) (Figures S36A–S36C).

### Oxidatively resistant, active-site-modified NS protects RGCs against glaucoma damage

**Figure 7:**
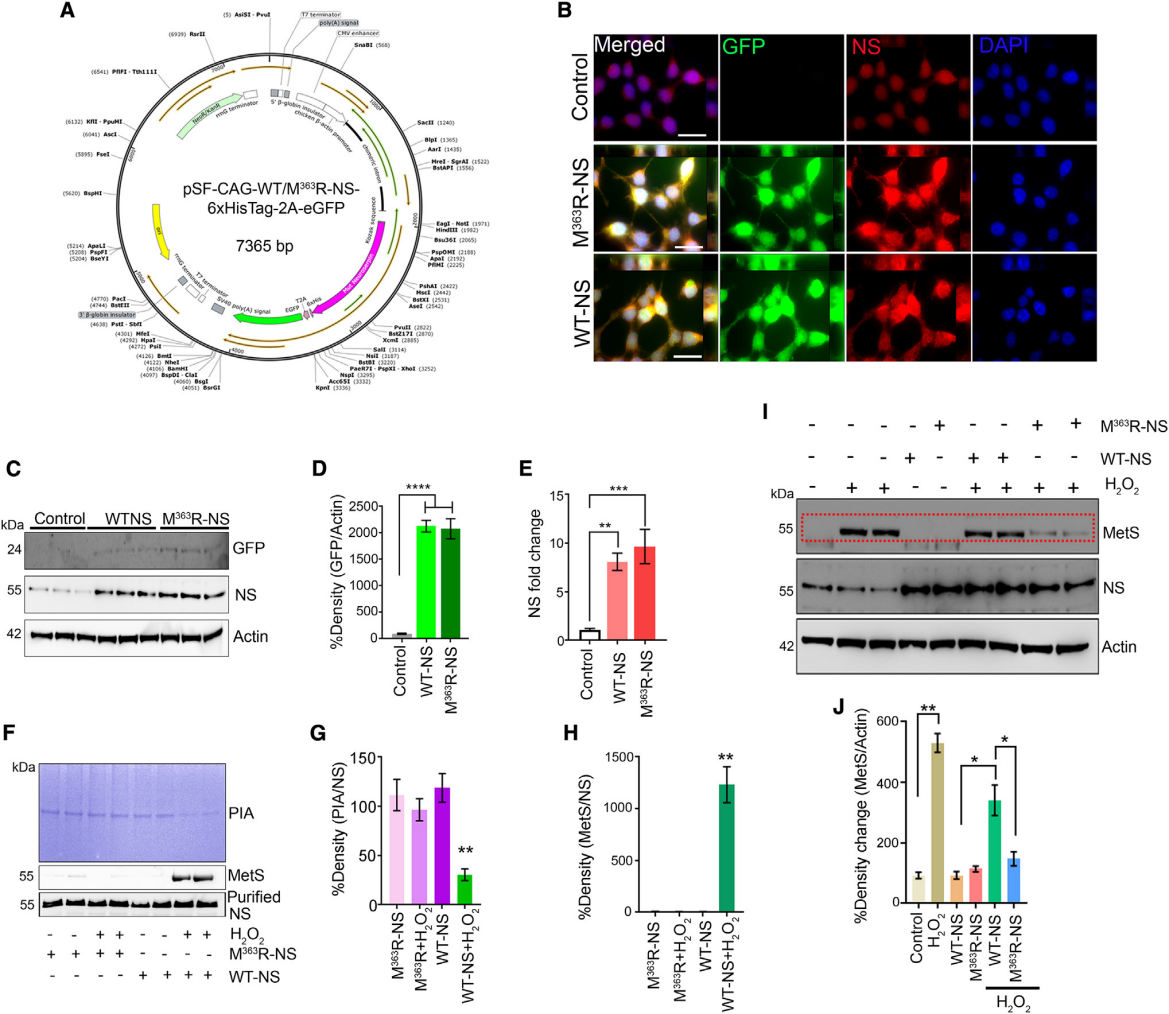
Effect of M^363^R mutagenesis on NS susceptibility to oxidation (A) Map of the pSF plasmid vector expressing EGFP, WT NS/M^363^R-NS linked with the His_6_ tag under the CAG hybrid promoter fused to the ampicillin-resistance gene. (B) Control and WT NS plasmid- and M^363^R-NS plasmid-transfected SH-SY5Y cells were subjected to IF analysis with anti-GFP (green) and anti- NS (red). DAPI, blue. (C) WB revealed expression of GFP and NS in pSF-transfected SH-SY5Y cells. β-Actin was used as a loading control. (D) Densitometric quantification of GFP in WT and M^363^R-NS-transfected cell lysates (p < 0.0001). (E) Densitometric quantification of NS expression in SH-SY5Y cell lysates compared with the control (p < 0.0001). (F) Purified NS (2 μg) from SHSY5Y cells was subjected to H_2_O_2_ treatment (10 μM, 1 h), and its PIA was assessed by gelatin gel zymography. Immunoblots were also probed for MetS and NS reactivity using specific antibodies. (G) PIA changes were analyzed with respect to total NS blotted in each case, and the densitometric data were quantified and plotted. Data indicated a significant decrease in PIA activity for WT NS subjected to H_2_O_2_-induced oxidation (p < 0.004). (H) Changes in MetS activity were compared with total NS blotted in each case. Densitometric data were quantified and indicated a significant increase in MetS reactivity for WT NS subjected to H_2_O_2_-induced oxidation (p < 0.009). (I) SH-SY5Y cells overexpressing WT NS or the M^363^R-NS plasmid with or without H_2_O_2_ treatment were lysed and subjected to WB. The blots were probed for MetS reactivity, and NS expression was analyzed. Actin was used as a loading control. (J) Densitometric evaluation of changes in MetS reactivity in WT and Mut NS-expressing cells exposed to H_2_O_2_ oxidative stress conditions indicated significantly less MetS reactivity in M^363^R-NS-expressing cells exposed to H_2_O_2_ (p < 0.03) compared with WT NS-expressing cells exposed to similar conditions. n = 3 independent experiments each. Scale bars, 5 μm).

The reactive-site loop of NS contains an exposed methionine residue that, like some other serpins, is highly susceptible to oxidation into methionine sulfoxide (MetS).^36^^,^^39^^,^^40^ The modified neuroserpin plasmid construct (pSF-CAG-WT/M^363^R-NS-His_6_Tag-2A-EGFP; Figure 7A) was transduced into SH-SY5Y cells WT NS and M^363^R-NS, and cell lysates were probed and quantified for GFP and NS expression (Figures 7B–7E). Subsequent oxidative stress using 10 μM H_2_O_2_ significantly reduced PIA in control (p < 0.005) and WT NS treated cells (p < 0.0008). In contrast, cells overexpressing M^363^R-NS did not show a reduction in PIA after H_2_O_2_ treatment (Figure S37).

Methionine oxidation in WT and mutated NS molecules and functional effects on PIA were examined by incubating purified WT NS and modified M^363^R-NS with H_2_O_2_, resulting in a significant decrease in PIA in just the WT NS molecule (Figures 7F and 7G) (p < 0.004) that correlated with increased MetS reactivity (p < 0.009) (Figures 7F and 7H). PIA and MetS reactivity were unchanged when M^363^R-NS was treated with H_2_O_2_ (Figures 7F–7H). In SH-SY5Y cells transfected with WT NS or M^363^R-NS and then stressed with H_2_O_2_, much higher MetS reactivity was observed for WT NS over M^363^R-NS-transfected cells (p < 0.003) (Figures 7I and 7J). Further, incubation of SH-SY5Y cells with H_2_O_2_ significantly reduced neurite outgrowth, whereas M^363^R-NS-overexpressing cells exhibited extended neurites under oxidative stress conditions (Figure S38).

To compare the effectiveness of WT NS and M^363^R-NS in protecting the retina against glaucomatous injury, animals were subjected to experimental glaucoma, where a comparable IOP increase was observed 8 weeks after microbead administration (control, 9.95 ± 0.34 mm Hg; glaucoma, 23.76 ± 2.53 mm Hg; glaucoma + WT NS, 23.34 ± 2.41 mm Hg; glaucoma + M^363^R-NS, 23.4 ± 2.36 mm Hg) (Figure S39A). Mouse retinas subjected to WT NS and M^363^R-NS administration under high-IOP conditions were examined for inner retinal functional changes using pSTR, H&E staining, and TUNEL. The data revealed that pSTR amplitudes were reduced under glaucomatous conditions (p < 0.0001) but protected in M^363^R-NS animals (p < 0.04) (Figures S40A and S40B). Whole retinal scotopic a- and b-wave ERG amplitudes remained relatively unaltered in control, glaucoma, glaucoma + WT NS, and glaucoma + M^363^R-NS treatment with glaucoma (Figures S39B–S39E). Correspondingly, GCL density in M^363^R-NS upregulation (Figures S40C and S40D) was significantly higher compared with glaucoma and glaucoma + WT NS (p < 0.008). These findings were also supported by TUNEL staining observations (p < 0.0001) (Figures S40E and S40F).

We next investigated whether WT NS or M^363^R-NS-administered groups exhibited changes in MetS reactivity under glaucomatous conditions. Enhanced MetS fluorescence was predominately localized to the inner retina in glaucoma and glaucoma + WT NS mice (Figures S41A–S41F). Densitometric quantification revealed significantly elevated MetS fluorescence with WT NS treatment under high-IOP conditions (p < 0.04) compared with M^363^R-NS-treated retinas (Figures S41G and S41H).

Biochemical analysis of ON sections demonstrated that pNFH levels in WT NS and M^363^R-NS-treated mice were not changed under healthy conditions but reduced in glaucoma and glaucoma + WT NS. In addition, pNFH levels were significantly increased in glaucomatous animals subjected to M^363^R-NS treatment (p < 0.0001) (Figures S42A and S42B). In contrast, IBA1 immunostaining was significantly elevated in glaucoma retinas compared with healthy, WT NS, and M^363^R-NS treated retinas (p < 0.0005). IBA1 immunoreactivity was relatively decreased (p < 0.003) in glaucomatous animals subjected to M^363^R-NS compared with WT NS treatment (Figures S42C and S42D). Retinal tissue PIA and NS activity were significantly elevated in M^363^R-NS mice compared with WT NS glaucomatous mice (p < 0.01) (Figures S43A–S43D). In contrast, MetS reactivity in glaucoma and glaucoma + WT NS overexpression was significantly higher compared with glaucoma + M^363^R-NS (p < 0.006) (Figures S43A and S43E). Retinal WB analysis showed that beclin 1 (p < 0.01) and *LC3B-II/I* (p < 0.007) were significantly reduced in WT NS and M^363^R-NS-treated retinas in the control IOP group. However, enhanced autophagy markers in glaucoma were significantly decreased in M^363^R-NS-treated mice compared with WT NS mice (p < 0.008 and p < 0.04, respectively) (Figures S44A–S44C). Assessment of synaptophysin and PSD95 showed modulation in response to WT NS and M^363^R-NS only under glaucomatous conditions but not in control IOP animals. Synaptophysin was decreased in the retina under glaucomatous conditions (p < 0.05), while levels were rescued in M^363^R-NS (p < 0.009) mice. Conversely, PSD95 was enhanced in glaucoma (p < 0.05) and significantly reduced in response to M^363^R-NS treatment (p < 0.05) (Figures S45A–S45C).

### M^363^R-NS administration rescues RGC degenerative glaucoma changes in *NS*^−/−^ mice

**Figure 8:**
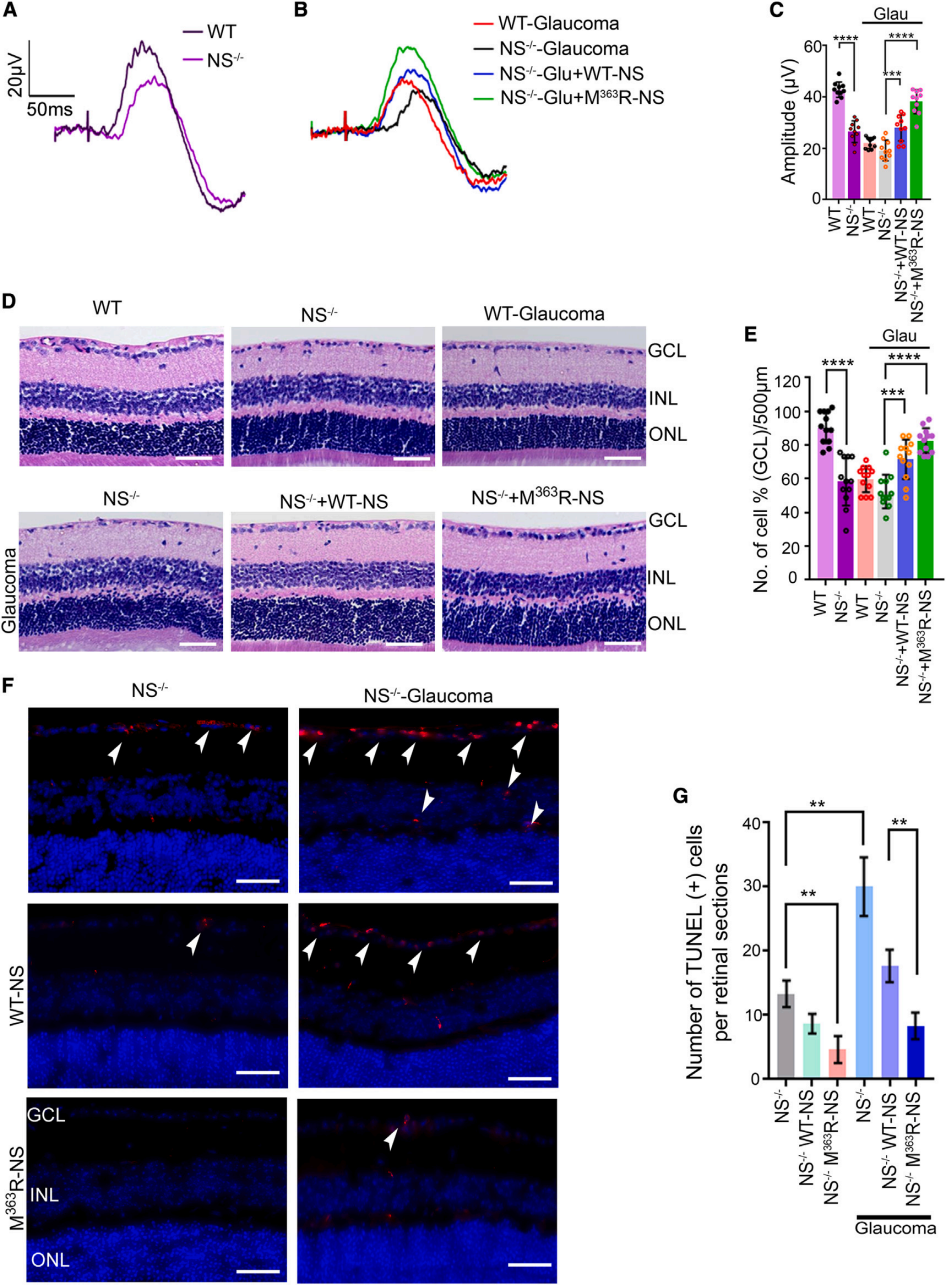
Modified M^363^R NS administration in *NS*^−/−^ mice protects against retinal deficits in chronic glaucoma (A and B) pSTR traces in WT and *NS*^−/−^ mice under control conditions (A) and (B) pSTR traces in WT and *NS*^−/−^-, *NS*^−/−^ + WT-NS-, and *NS*^−/−^ + M^363^R-NS-administered mice under experimental glaucoma conditions. (C) Quantification of pSTR amplitudes demonstrated significantly lower pSTR amplitudes in *NS*^−/−^ compared with WT mice at 3 months of age (p < 0.0001, n = 10 animals/group). Induction of experimental glaucoma reduced pSTR amplitudes in WT and *NS*^−/−^ mouse retinas. NS administration (10 μmol/L; volume, 2 μL; once weekly for 8 weeks) demonstrated enhanced pSTR amplitudes; however, protection with M^363^R-NS was much higher compared with WT NS treatment in experimental glaucoma (p < 0.031, n = 10 animals/group). (D) H&E analysis of retinal sections from WT and *NS*^−/−^ mice in control and glaucoma along with WT and M^363^R-NS-treated groups under glaucoma conditions. Arrows indicate the GCL. (E) There was a significant decrease in GCL density in *NS*^−/−^ compared with WT mouse retinas under the control condition (p < 0.0001). M^363^R-NS administration in experimental glaucoma significantly protected GCL density compared with the WT NS-administered group (p < 0.007, n = 4 animals, 3 sections/animal). (F and G) TUNEL apoptosis staining and its quantification revealed positive cells in *NS*^−/−^ mouse retinas, which was significantly enhanced in glaucoma (p < 0.0001). WT NS (p < 0.001 control, p < 0.0001 glaucoma) and M^363^R-NS (p < 0.008 control, p < 0.002 glaucoma) administration under control and glaucoma conditions led to a significant reduction in TUNEL^+^ staining in the GCL. TUNEL^+^ cells (red) are predominantly in the GCL layer. Reduced TUNEL^+^ cells were evident in animals subjected to M^363^R-NS administration compared with the WT NS-administered group. DAPI, blue. n = 3 animals/group. Scale bars, 50 μm. Graphs show means ± SEM, and p values were obtained using Student’s t test.

Because *NS*^−/−^ mice showed exacerbated inner retinal dysfunction and loss of RGCs during experimental glaucoma, we examined the effect of direct M^363^R-NS administration on that pathological phenotype. IOP was monitored weekly after microbead injection in WT and *NS*^−/−^ mice to ensure statistically significant, sustained IOP elevation (p < 0.05) (Figures S46A and S46B). pSTR was partially rescued in the WT NS and M^363^R-NS groups, which underwent exogenous NS administration following induction of experimental glaucoma. However, M^363^R-NS administration led to increased rescue of inner retinal function compared with the WT NS treated group (p < 0.031) (Figures 8A–8C). Full-field flash ERG responses in untreated controls (Figures S46B and S46C) and experimental glaucoma treated with WT NS and M^363^R-NS did not show any significant changes to a-wave or b-wave amplitudes (Figures S46D and S46E). H&E staining of *NS*^−/−^ mouse retinal sections and age-matched WT C57BL/6J sections showed gross morphological changes in the adult retina because of loss of NS (Figure 8D), including a statistically significant loss of GCL density in NS-ablated mice (p < 0.05) compared with WT controls (Figures 8D and 8E) (p < 0.0001). Zymography revealed a 90% PIA loss in healthy and glaucomatous *NS*^−/−^ mice (Figures S47A and S47B). Conversely, *NS*^−/−^ glaucoma mice injected with exogenous M^363^R-NS demonstrated statistically significant preservation of GCL and INL and significantly higher PIA activity than those who received WT NS injections (Figure 8D; cells/500 μm; p < 0.0089; Figure S47), and those findings were supported by significantly reduced TUNEL apoptosis staining in the RGCs layer (p < 0.0001) (Figures 8F and 8G).

pNFH levels were diminished in *NS*^−/−^ glaucoma mice with a 3- to 4-fold decrease compared with WT mice, indicating that ON axons in *NS*^−/−^ mice were more susceptible to glaucoma-induced damage (p < 0.0001) (Figures S48A and S48B). Administration of M^363^R-NS to experimental glaucoma *NS*^−/−^ mice led to 1.5- to 2-fold increased pNFH immunoreactivity compared with WT NS treatment in *NS*^−/−^ mice with high IOP (Figures S48A and S48B). In contrast, ON IBA1 staining was significantly elevated under healthy conditions (p < 0.0001), and a greater increase in IBA1 was observed in *NS*^−/−^ mice compared with WT mice with high IOP (p < 0.002) (Figures S48C and S48D). IBA1 immunostaining was significantly reduced in WT NS (p < 0.0001) treated mice compared with *NS*^−/−^ mice in glaucoma, but a greater decrease in IBA immunoreactivity was observed with M^363^R-NS treatment compared with WT NS treatment (p < 0.0005) (Figures S48C and S48D). M^363^R-NS treatment produced a 1.5- and 1.4-fold increase in synaptophysin expression under healthy and glaucomatous conditions in *NS*^−/−^ mice compared with WT NS mice (Figures S49A and S49B). Conversely, PSD95 levels were enhanced in *NS*^−/−^ glaucoma (p < 0.02) and significantly reduced in response to M^363^R-NS treatment (p < 0.04; p < 0.02) compared with WT NS (Figures S49A and S49C). WB analysis of retinal tissues showed that beclin 1 and *LC3B-II/I* were not altered in *NS*^−/−^ mice under normal IOP conditions, even after treatment with WT NS and M^363^R-NS retinal overexpression (Figures S50A–S50C). Moreover, induction of high IOP in *NS*^−/−^ mice significantly elevated beclin 1 (p < 0.005) and *LC3B-II/I* (p < 0.005). However, enhanced autophagy markers were significantly decreased in M^363^R-NS-treated compared with WT NS mice during glaucoma (p < 0.04 and p < 0.02, respectively) (Figures S50A–S50C).

## Discussion

In a previous human study, deficiency of NS was shown to be responsible for altered spine-synapse density and synaptic plasticity in the brain.^41^ This study highlights the potential novel roles of the serine protease inhibitor NS in preserving RGC integrity and limiting axonal loss during glaucoma. These changes were identified using an *NS*^−/−^ mouse model where loss of NS expression was exacerbated under experimental glaucoma pathology conditions. Consistent with this, *NS*^−/−^ mice showed exacerbated neuronal cell death.^13^ Given its key role in neuronal cells, we extended the analysis of NS function to RGCs, demonstrating strong expression of NS.^36^ We investigated NS expression in the retinas of WT, *NS*^−/−^, and *NS*^+/+ Tg^ mice.

Our studies confirmed loss of NS expression in *NS*^−/−^ and high expression levels in retinas of *NS*^+/+ Tg^ mice, respectively. Our results demonstrate an overall age-dependent decline in inner retinal structural and functional parameters after 3 months in *NS*^−/−^ mice. Retinal thickness and pSTR amplitudes were further reduced progressively in *NS*^−/−^ mice as they aged to 6 and 12 months, and this may be attributed to reduced RGC dendrites that formed a fibril network in the GCL+IPL layer. Corroborating these findings, reduced ON axonal density further established degenerative changes in RGCs of mice lacking the serine protease inhibitor NS. However, *NS*^+/+ Tg^ young and aged mice demonstrated significant functional protection in pSTR amplitudes and relative preservation of the GCL and axons count. Because NS’s primary function is to maintain synaptic plasticity under control conditions,^32^^,^^41^ this may explain its inclination toward maintaining the overall structural integrity of RGCs and their respective axons in the overexpression model. The overall data from *NS*^−/−^ and *NS*^+/+ Tg^ mice indicate that NS plays an essential role in preserving the retina and ON axons. Whole retinal full-field ERG amplitudes were not significantly affected in *NS*^−/−^ or *NS*^+/+ Tg^ mice, suggesting that NS modulation preferentially affects inner retinal function.

To understand the role of NS in glaucoma, we investigated the effects of NS ablation or neutralization in the retina using intravitreal administration of an NS-neutralizing antibody. Under glaucomatous conditions, this was found to be detrimental to the inner retina, with increased GCL thinning and ON axonal loss. In contrast, *NS*^+/+ Tg^ mice overexpressing NS s did not exhibit GCL degeneration and axonal loss under glaucomatous conditions. This confirms that increased NS levels in glaucoma can protect the retina against glaucoma injury. In an animal model of elevated IOP, serine proteases, particularly tPA upregulation, were observed to induce RGC loss, while inhibiting tPA activity imparted RGC protection.^42^^,^^43^ These findings confirmed that loss of NS or its neutralization in the retina may lead to degenerative changes in inner retinal laminar structure and function, exacerbated by pressure-induced damage to retinal neurons.

Numerous studies have shown that NS interacts with plasmin and its activators,^36^^,^^44^ and there is an increasing understanding of the association between tPA excitotoxicity and retinal degeneration.^42^^,^^43^ *tPA*^−/−^ mice are resistant to retinal damage in an excitotoxin-treated retinal model and an ischemic-perfusion model that is thought to be related to increased IOP.^45^ Equally, enhanced tPA and uPA have been shown to induce RGC loss in different animal models of RGC damage.^42^^,^^43^ An important observation made by Siao et al.^46^ is that tPA activity is responsible for microglial activation in neuronal cell cultures and may represent one mechanism of neurodegeneration. Reactive microglia have been reported in the ON and retina in an ON injury model.^47^^,^^48^ In human glaucoma postmortem tissue, abnormal microglial distribution and reactivity have been detected in the ON head.^49^^,^^50^ Our findings identify significant changes in the activation profile of microglial markers in the ON without NS. Various studies corroborate this and report that microglial activation may exert neurotoxic effects by releasing inflammatory cytokines, such as tumor necrosis factor alpha (TNF-α), interleukin-1β (IL-1β), IL-6, matrix metalloproteinases, Fas ligands, and reactive oxygen species.^51^^,^^52^ Other studies have shown that tPA can induce proinflammatory pathways.^13^^,^^53^ Tsirka et al.^54^ have reported previously that proteolytic activation of zymogen plg to the broad-spectrum protease plasmin by tPA is required for neurodegeneration.

Furthermore, dual involvement of tPA has been reported, first in converting plg into active plasmin and second by mediating microglial activation during excitotoxicity, leading to neuronal death.^55^ In an animal model of middle cerebral artery occlusion (MCAO), *NS*^−/−^ mice showed greater infarct size compared with WT mice, which correlates with an increase in activated TNF-α-producing microglia.^13^ Furthermore, exogenous administration of active NS significantly reduced the stroke size, suggesting that inhibition of proteinase activity, possibly tPA activity, was necessary for the neuroprotective effect of NS.^12^ To fit this hypothesis, we observed a significant increase in IBA1 immunoreactivity in ON tissue of 3- and 12-month-old *NS*^−/−^ mice. To confirm that the presence of NS in the retina is neuroprotective, our results using an NS-neutralizing antibody demonstrated microglia morphological changes, an increase in microglial cell activation, and its migration from the GCL to the entire retina. Induction of glaucoma in these animals showed a further significant increase in microglia activation. These data suggest that loss of NS can boost the effects observed after microglial activation. Hence, developing strategies that can preferentially inhibit plasmin and tPA and overexpress NS s may offer new strategic approaches that could modulate microglial activation, further protecting RGCs against glaucomatous damage.

Reumann et al.^41^ have reported previously that *NS*^−/−^ mice show a decline in synaptic density in the hippocampal CA1 region, with elevated expression of the postsynaptic protein PSD95. Reduced synaptic potentiation and decreased long-term potentiation (LTP) in *NS*^−/−^ mice led to shortfalls of cognitive and socializing ability in behavioral studies, which can be associated with neuropsychiatric disorders.^41^ Increased NS and tPA expression have also been reported in the developing stage of the visual cortex, during the essential phase in the synaptic linking of neurons.^11^^,^^56^ In this study, it is not clear whether the effect of NS on PSD95 expression occurs directly or indirectly. We observed no change in synaptophysin expression in *NS*^−/−^ or *NS*^+/+ Tg^ mice in the retina. However, in correlation with previous reports, NS loss increased PSD95 expression in young and older *NS*^−/−^ mouse retinas, and this could represent compensation for synaptic damage. Previous studies have also shown that overexpression of NS in the hippocampus results in decreased PSD95 expression in a rat model, with no alteration of learning and memory processes related to the hippocampus region.^57^ In this study, we did not observe changes in synaptophysin and PSD95 expression in the retinas of *NS*^+/+ Tg^ mice. This may explain the significant protection of RGCs in *NS*^+/+ Tg^ mice for up to 12 months. In glaucoma, an alteration in synapses and loss of dendritic branching is typically reported as an early feature preceding RGC cell death.^58^ Here, we show that the pre-synaptic marker synaptophysin is gradually decreased in the retina following 8 weeks of high IOP exposure. However, the post-synaptic marker PSD95 was increased after 8 weeks. The decrease in synaptophysin expression in the retina may be due to the significant loss of RGCs in glaucoma; however, increased expression of PSD95 may suggest a compensatory mechanism to restore synaptic connections between RGCs and bipolar cells that were lost because of apoptosis. Similar results were observed when NS was neutralized in the retina using anti-NS antibody administration, which showed downregulation of synaptophysin but higher levels of PSD95 in control and glaucomatous retinas. Further investigations are needed to elucidate the synaptic phenotype in the NS ablation mouse model.

In addition, we studied the neurofilament protein phosphorylation state in animal ON axons exposed to experimental glaucoma in WT, *NS*^−/−^, and *NS*^+/+ Tg^ mice because the phosphoform of neurofilament is a foster marker for axonal injury, loss, and degeneration.^59^ Phosphorylation of neurofilaments plays a key role in formation of neurofilament cross-bridges and is highly involved in axonal transport and plasticity.^60^ A decrease in pNFH and increased dephosphorylation of heavy-chain neurofilaments have been described in experimental models of glaucoma in mice and monkeys.^61^^,^^62^ Corroborating previous studies, we observed decreased pNFH levels in *NS*^−/−^ mice and animals treated with NS antibodies in normal and experimental glaucoma models. This suggests that the absence of NS may be involved in changes to the ON axon cytoskeleton and that induction of glaucoma may exacerbate the phosphorylated neurofilaments that are transported down into the axon, where they maintain and support the neuronal cytoskeleton.

Autophagy has been reported to be involved in several brain and retinal neurodegenerative disorders, including glaucoma.^63^ Autophagy has been shown to induce axonal degeneration of RGCs after an ON crush model.^64^ Increased LC3 immunoreactivity and accumulation of LC3II in the GCL have been demonstrated between 6 and 24 h after a transient IOP increase in rats.^65^^,^^66^^,^^67^ Our study indicates that NS does not directly inhibit accumulation of LC3-II/I ratio and beclin1 autophagy markers in the retina under normal conditions. In contrast, experimental glaucoma resulted in upregulation of autophagy, and neutralization of NS was associated with activation of autophagy networks. Similar findings of retinal neurodegeneration in mouse models of glaucoma have been reported, where chronic IOP elevation increased the level of autophagy markers to promote RGC damage and exacerbated axonal degeneration.^68^

Therapeutic gene therapy has been approved for correction of the genetic defect carried by patients with a retinal pigment epithelial 65-kDa protein (RPE65) mutation associated with retinal dystrophy.^69^^,^^70^^,^^71^ We have previously modulated Shp2 protein through AAVs in RGCs in a rodent model to study the Brain-derived neurotrophic factor/tropomyosin-related kinase receptor type B (BDNF/TrkB) signaling pathway and demonstrated that silencing Shp2 activity in high-IOP retinas is neuroprotective.^72^ At the cellular level, we investigated rescue by AAV-mediated NS overexpression in experimental glaucoma in WT and *NS*^−/−^ mouse retinas. We observed sustained overexpression of NS for over 2 months in the retina, utilizing EGFP as a marker. In animal models of stroke, overexpression of NS has been shown to reduce ischemic damage, including ECM degradation, microglia activation, and blood-brain barrier leakage *in vivo*, whereas *NS*^−/−^ mice have been reported to have worsened ischemic damage, attributed to tPA-mediated activation of microglia.^13^ Animal eyes treated with exogenous recombinant NS showed electroretinogram b-wave amplitude recovery 7 days post injury and attenuated numbers of TUNEL^+^ cells throughout the GCL and INL within 24 h of ischemic reperfusion injury.^16^ In this study, we determined that overexpression of NS protected against inner retinal laminar structural and functional deficits induced by experimental glaucoma in WT and *NS*^−/−^ mice. Consistent with previous findings, NS upregulation not only protected RGCs in WT glaucoma retinas but also rescued the phenotype in *NS*^−/−^ mice and ameliorated microglia activation and autophagy efflux.

The endogenous NS protein possesses a Met^363^ residue in its reactive site loop.^32^^,^^73^ Oxidative stress (e.g., by exposure to H_2_O_2_ or HOCl) can modify methionine to MetS, as shown previously with the alpha-1-proteinase inhibitor.^39^^,^^40^^,^^74^ We have demonstrated previously that NS is susceptible to oxidation at that methionine residue, which is associated with reduced PIA in glaucoma.^36^ As a result, we developed a modified M^363^R NS resistant to oxidation. The present study demonstrated that M^363^R is resistant to oxidative inactivation, as evidenced by significantly lower MetS reactivity *in vitro* and *in vivo*. This study also showed phenotypic rescue in *NS*^−/−^ control and glaucomatous mice by overexpressing M^363^R-modified NS in retinal neurons.

In conclusion, NS is a critical component for retinal health and development, and its presence and activity can either exacerbate or protect RGCs from damage in experimental glaucoma. By exposing animals to glaucoma, we highlight the potential contribution of NS oxidation in mediating glaucomatous damage to the retina and propose a modified gene therapy candidate that may represent a therapeutic option that results in neuroprotection during glaucoma.

## Material and methods

### Chemicals

The primary antibodies NS (ab33077), GFP (ab290 and ab1218), synaptophysin (ab32127), βIII-tubulin (ab7751 and ab215037), NeuN (ab104224 and ab104225), and β-actin were obtained from Abcam (VIC, Australia). Beclin1 (3495) and LC3B I/II (12741) antibodies were obtained from Cell Signaling Technology (USA), and anti-MetS (600160), anti-IBA1 (019-19741), anti-pNFH (801601), anti-PSD95 (516900), and anti-Brn3a (MAB1585) were from Cayman Chemical (USA), Novachem (Australia), BioLegend (USA), Thermo Fisher Scientific (USA), and Merck (Germany), respectively. Anti-rabbit horseradish peroxidase (HRP; HAF008), anti-mouse HRP (HAF018), and anti-goat IgG-HRP (HAF109) secondary antibodies for western blotting were obtained from R&D Systems (USA). Alexa Fluor 488-, 555-, and 594-labeled secondary antibodies were obtained from Jackson ImmunoResearch Laboratories/Life Technologies. The TUNEL kit was obtained from Promega and ProLong mounting medium with DAPI from Molecular Probes. The bicinchoninic acid assay (BCA) protein detection kit and Supersignal West Pico chemiluminescent substrate were from Pierce (Rockford, IL, USA). All other chemicals and reagents were purchased from Sigma or Invitrogen.

### Cell culture and viral transduction

The SH-SY5Y cells were obtained from the American Type Culture Collection (ATCC; VA, USA). The cells were grown and maintained as described previously^75^ in Dulbecco’s modified Eagle’s medium (DMEM) supplemented with 10% fetal bovine serum (FBS; Life Technologies), penicillin (100 U/mL), streptomycin (100 U/mL), and 2 mM L–glutamine at 37°C in a humidified atmosphere of air containing 5% CO_2_. Approximately 2.0 × 10^5^ SH-SY5Y cells were seeded in 6-well culture dishes 6–12 h before treatment and grown to 80% confluency prior to transduction. Cells were pre-differentiated with 10 μM all-*trans*-retinoic acid (Sigma) for 2 days. The medium was changed to retinoic acid medium without antibiotics, and viral transduction (1 μL of virus + 100 μL culture medium with retinoic acid) was carried out initially by incubating cells with either of two different viral constructs (AAV2-GFP and AAV2-NS) for 48 h. The transduction concentration of the virus was 10^9^ genome particles/well. After 48 h, the transfection medium was replaced with fresh retinoic acid medium.

### Plasmids and DNA

To assemble the NS CAG construct, a linear DNA fragment containing the CAG promoter, a poly(A) tail, the SV40 promoter, the neomycin gene, and a second poly(A) tail was synthesized (GenScript). The His-tagged human NS (WT) construct was cloned into pUC57 using the EcoRV cloning site. The primer used for WT NS (sense, 5′-AATGCTGTCTATTTCAAGGG-3′; anti-sense, 5′-TCGGTAGTGTTTAAGGGGT-3′). M^363^R modified neuroserpin of the full length was generated by site-directed mutagenesis (SDM). The primer used for SDM was M^363^R (sense, 5′-AGTAGGAGGGCTGTG-3′; antisense, 5′-TCATCCTCCCGACAC-3′). After sequencing, the WT and modified cDNAs were excised from the sequencing vector and cloned into the pSF-CAG-EGFP mammalian expression vector (Oxford Genetics, UK).

### Animals

WT, *NS*^−/−^, and *NS*^+/+ Tg^ mice (∼4 weeks, C57BL/6J background of either sex) were obtained from the animal facility of the University Medical Center Hamburg-Eppendorf and bred in the Macquarie University animal facility. Briefly, *NS*^−/−^ mice were generated by insertion of a neomycin cassette into the second coding exon.^10^^,^^31^ The NS transgenic mouse was generated by constructing a Thy-1 vector with 1,400-kb human NS cDNA as a gene of interest and injected into fertilized oocytes,^76^ and the genotype was screened by PCR using specific NS and neomycin gene primers as described previously.^31^ The *NS*^−/−^ and *NS*^+/+ Tg^ mice were bred with C57BL/6J mice for multiple generations, and animal colonies were established. Animals were housed at a constant temperature (21°C ± 2°C) on a 12-h light/12-h dark cycle and provided *ad libitum* access to regular lab chow and water throughout the experiments. All animal experiments were approved by the University Animal Ethics Committee (ARA 2018_011). Animal experimental procedures were performed in accordance with the Australian Code of Practice for the Care and Use of Animals for Scientific Purposes and the guidelines of The Association of Research in Vision and Opthalmology (ARVO) Statement for the Use of Animals in Ophthalmic and Vision Research. Animals were anesthetized through intraperitoneal (i.p.) injections of ketamine (50 mg/kg) and medetomidine (0.5 mg/kg) for procedures, including retinal electrophysiological recordings, and for acute ocular hypertension models.

### Chronic ocular hypertension animal model

A chronic model of RGC degeneration was generated by exposing mouse retinas to consistently enhanced IOP via intracameral injection of polystyrene microbeads (FluoSpheres polystyrene microspheres, 10 μm) as reported previously.^77^ Briefly, under isoflurane (2% v/v) anesthesia, mice received intraocular injections (2 μL containing approximately 5 × 10^3^ microbeads/mL) weekly for 8 weeks until a sustained increase in IOP was observed compared with contralateral control eyes. IOP was measured non-invasively weekly using an average of 4 consecutive readings using a hand-held rebound tonometer (Icare Tonovet, Helsinki, Finland) under isoflurane anesthesia. Animals were euthanized prior to tissue harvesting for further analysis.

### AAV construct design

AAV backbone serotype 2 (AAV2) vectors were produced commercially by Vector Laboratories (PA, USA). Briefly, the human NS cDNA (UniProt: BC018043) was placed under modified transcriptional control of cytomegalovirus (CMV) and chicken β-actin rabbit β-globin (CBA), known as a CMV early enhancer/chicken β-actin (CAG2) hybrid promoter. Shortened WPRE poly(A) was inserted into the AAV2 backbone with a green fluorescence protein (EGFP) vector (AAV2-CAG2-EGFP-WPRE) and NS overexpression (AAV2-CAG2-EGFP-T2A-hNS-WPRE or AAV2-NS). EGFP and NS gene sequences were driven by the CAG2 hybrid promoter and included a 2A linker region. The EGFP control vector was also expressed under control of the CAG promoter flanked by inverted terminal repeats (AAV-GFP) and used as a control for NS overexpression.

### Intravitreal AAV injections

Mice were anesthetized and eyes dilated using 1% tropicamide as reported previously.^38^ An NS antibody (anti-NS) or IgG antibody (positive control) (2 μL) was administered intravitreally weekly for 8 weeks in WT mice in negative control and microbead-injected eyes, and animals were monitored for 8 weeks. The AAV2 construct (final concentration, 1.8 × 10^12^ genome copies ((GC)/mL) was carefully administered (2 μL) through the sclera at a 45° angle into the vitreous toward the *ora serrata* and posterior to the temporal limbus, avoiding contact with the lens. The injection was performed using a 33G needle connected to a 5-μL Hamilton syringe, guided by a surgical microscope (Carl Zeiss) to facilitate accurate focusing. A period of 30 s was allowed before removing the needle to permit diffusion of the virus and prevent leakage from the injection track. Animals were monitored for 2 months after AAV2 administration. Purified protein of NS WT and mutated NS (Mut-NS) (dose, 10 μmol/L; volume, 2 μL)^16^ was administered intravitreally as described above in WT and NS^−/−^ mice under control and experimental glaucoma conditions.^16^

### ERG

ERG recordings were performed as described previously.^38^^,^^78^ Briefly, mice were dark adapted overnight and anesthetized with ketamine and medetomidine (75 and 0.5 mg/kg, respectively). Pupils were dilated using 1% tropicamide, and a topical anesthetic (1% alcaine) was applied to the cornea. Ground and reference electrodes were placed subcutaneously into the tail and forehead of the animal, respectively. A solid custom-made gold ring recording electrode (Roland Consult, Germany) was placed on each eye in contact with the cornea, and methylcellulose was applied to maintain contact between the cornea and the electrode. ERGs were recorded using a flash intensity of 3 log cd (candela)·s/m^2^ (Phoenix Technology, USA). For pSTRs, dim stimulation using flash intensities of −4.5 log cd·s/m^2^ (0.5 Hz) was delivered 30 times. pSTR amplitudes were measured from baseline to the positive peak observed around 120 ms. For all ERG recordings, the a-wave amplitude was measured from baseline to the a-wave trough; the b-wave was measured from the a-wave trough to the peak of the b-wave.^79^

### OCT and fundus imaging

The Phoenix Technology Micron IV was used for OCT and fundus imaging.^80^ Mice were anesthetized with ketamine and medetomidine as described previously, and pupils were dilated with 1% tropicamide, with mice placed on adjustable stages with heating pads to maintain warmth during procedures. OCT lubricant was applied on the eye to keep continuous contact between the eye and lens. The OCT software was set up as described in the manufacturer’s instructions (Micron IV), with OCT images obtained after alignment of 50 real-time frame captures. For retinal thickness quantification, 15 OCT images per eye were obtained for each group (n = 4 per group). StreamPix software for fundus imaging was used according to the manufacturer’s instructions. The image was captured at two different channels: bright light for normal imaging and a blue excitor/yellow barrier for fluorescent expression of GFP.

### Retinal GCL, ON histology, and axonal counting

Animals were euthanized with an overdose of pentobarbitone (100 mg/kg), followed by transcardial perfusion using 4% paraformaldehyde. Eyes were marked for orientation before harvesting and then fixed in 4% paraformaldehyde. Eye and ON tissue histology was performed using optimized methodology,^36^^,^^38^^,^^72^ where 5- to 7-μm-thick sagittal sections of eyes were obtained and subjected to H&E staining as described previously.^79^^,^^81^ GCL density was determined by manual cell count using light microscopy (Carl Zeiss). For GCL density, 6 sections from each eye (3 from the superior and 3 from the inferior retina), 500 μm from both sides of the optic disc, were subjected to histochemical staining and analyzed for quantification. For the ON, 2- to 5-μm-thick cross-sections were prepared and stained with toluidine blue (TB) as reported previously.^82^^,^^83^ Light microscopy images were captured at low (10×) and high (63×) magnification using a microscope (Carl Zeiss Axio Imager). The axons were counted across the entire cross-section photographed at high magnification (10^−2^ mm^2^). Six images for each ON were analyzed to compute axon counts per group (n = 4 animal ONs/group).

### SDS-PAGE, western blotting, and zymography

Eyes were enucleated, and the ON head (ONH) regions of the retina were surgically excised from retinas under a microscope and lysed (20 mM HEPES (4-(2-hydroxyethyl)-1-piperazineethanesulfonic acid) [pH 7.4], 1% Triton X-100, 1 mM EDTA) using PhosSTOP (Sigma) and a protease inhibitor cocktail (Sigma). Protein concentrations were measured using BCA.^84^ Proteins were resolved using 10% SDS-PAGE and transferred to polyvinylidene fluoride (PVDF) membranes (Invitrogen). Membranes were blocked in Tris-buffered saline (TBS) (20 mM Tris-HCl [pH 7.4], 100 mM NaCl, and 0.1% Tween 20) containing 5% skimmed milk^85^^,^^86^ and incubated overnight with one of the antibodies as indicated: anti-GFP (1:1,000), anti-NS (1:1,000), anti-synaptophysin (1:2,000), anti-beclin-1 (1:1,000), anti-LC3BII/I (1:1,000), anti-PSD95 (1:1,000), or anti-actin (1:5,000) overnight at 4°C (Table S1). Following primary antibody treatment, blots were incubated with HRP-linked secondary antibodies, and the signal was detected using the SuperSignal West Pico chemiluminescent substrate (Pierce). The protease inhibitory assay of NS was carried out by gelatin-embedded PAGE zymography. Briefly, retina or ONH lysate proteins were separated for zymography on precast 10% polyacrylamide gels containing 1% (w/v) gelatin (Life Technologies, NY, USA). After electrophoresis, gels were incubated at 37°C in 0.1 M sodium phosphate buffer (pH 7.4) containing plasmin (Sigma) for 1 h as described previously.^36^^,^^39^^,^^87^ The gel was subsequently incubated in 0.1% Coomassie blue (0.1% Coomassie blue dye in 40% ethanol [100%], 10% acetic acid [glacial], and 50% deionized water mixture) overnight at room temperature with continuous rocking. The gel was then de-stained with a de-staining solution (50% deionized water, 40% ethanol [100%], and 10% acetic acid [glacial]) until the background was clear. Dark blue bands against a light background after staining with Coomassie solution indicated serpin inhibitory activity. Bands were detected using an automated luminescent image analyzer (ImageQuant LAS 4000), and ImageJ (NIH, USA) was used to quantify band intensities.^88^

### IF analysis

Enucleated animal eyes and ON tissues were fixed for 2 h in 4% freshly prepared paraformaldehyde (PFA), washed three times with PBS to remove PFA, and incubated in 30% sucrose overnight for cryoprotection. Eyes were then embedded in tissue-Tek OCT cryostat embedding medium as described previously,^89^ flash frozen in liquid nitrogen, and stored at −80°C. Tissue sections 6–8 μm thick were prepared using a cryostat (Leica). Before immunostaining, slides were warmed at 37°C for 30 min and washed twice with PBS for 10 min. Using a Pep-Pen, the tissue area was circled and incubated in blocking buffer solution containing 5% goat serum, and sections were permeabilized with 0.3% Triton X-100 in 1× PBS^81^ for 60 min. The blocking buffer solution was aspirated, and sections were incubated with the indicated primary antibodies prepared in antibody dilution buffer (1× PBS/1% bovine serum albumin/0.3% Triton X-100) overnight at 4°C. The following antibody dilutions were used for immunohistochemistry: anti-NS (1:300), anti-GFP (1:300), anti-synaptophysin (1:500), anti-βIII-tubulin (1:300), anti-NeuN (1:250), anti-MetS (1:300), anti-Iba1 (1:250), anti-pNFH (1:300), and anti-Brn3a (1:250). After incubation, the slides were washed three times in PBS, and then sections were incubated with appropriately diluted secondary antibodies (Table S1) for 1 h in the dark at room temperature and again washed three times with PBS for 5 min. Sections were coverslipped with ProLong-DAPI mounting medium and kept overnight at room temperature for drying. To assess ON damage, the percentage of cross-section area covered by pNFH (anti-pNFH) and microglial activation (anti-IBA1) were measured in 10 cross-sections per ON and averaged for the total number of ON s in each mouse group. Imaging of stained sections was performed at the indicated wavelengths using a Carl Zeiss microscope. GFP^+^ total cells and NeuN ^+^ total cells were quantified manually using 10 sections from each animal eye (n = 4).

### TUNEL apoptosis assay

Cell apoptosis in animal retinas was detected using terminal deoxynucleotidyl transferase (TdT)-mediated TUNEL system (DeadEnd fluorometric TUNEL system, Promega) as previously described.^75^ PFA fixed frozen cryostat sections were warmed at 37°C for 30 min and washed 2 times with PBS for 5 min. Sections were permeabilized by adding 100 μL of a 20 μg/ml Proteinase K solution and allowed to incubate at room temperature for 10–15 min. The sections were then washed twice with PBS and re-fixed in 4% PFA for 5 min. Before addition of 100 μL equilibration buffer, slides were washed in PBS for 5 min. Labeling was performed by adding 50 μL of TdT reaction mix to the tissue. To prevent the tissue from drying and for even distribution of the mix, a plastic coverslip was placed over each slide, and they were incubated for 60 min at 37°C in a humidified chamber in the dark. Following incubation, the reaction was stopped by immersing the slides (without plastic coverslips) in 2× saline sodium citrate (SSC) buffer for 15 min. Slides were then washed with PBS, mounted with ProLong Antifade DAPI and directly analyzed for apoptotic cell staining using epi-fluorescence microscopy. The retinal sections of 5-7 μm thick each were used per experimental and control group (n = 3 animals/group), and data were quantified for TUNEL assay.

### Statistical analysis

Changes in ERG/STR amplitudes, retinal thickness, ON axonal density, western blot bands, gelatin zymography, histology, and TUNEL data were analyzed using GraphPad Prism (v.6.0) (GraphPad, San Diego, CA). All data are represented as the mean ± SEM. Statistical analysis was performed using Student’s t test for unpaired groups or ANOVA (one-way ANOVA) followed by Bonferroni’s post-hoc multiple comparisons test. All values are presented as mean ± SEM error bars for given n sizes. The significance value was set at p ≤ 0.05. All error bars indicate SEM in the figure legends.

## Data Availability

All data are available in the main text or the supplemental information.

## Supplemental information

Document S1. Figures S1–S50 and Table S1

Document S2. Article plus supplemental information
