# Supplementary material for: Neuroserpin gene therapy inhibits retinal ganglion cell apoptosis and promotes functional preservation in glaucoma
Source: Mol Ther. 2023 Mar 11;31(7):2056–76. doi: 10.1016/j.ymthe.2023.03.008 (PMC10362384; doi:10.1016/j.ymthe.2023.03.008)
Supplement: Document S1. Figures S1–S50 and Table S1 [file mmc1.pdf]

YMTHE, Volume *31*

Supplemental Information

Neuroserpin gene therapy inhibits retinal ganglion cell apoptosis and promotes functional preservation in glaucoma

Nitin Chitranshi, Rashi Rajput, Angela Godinez, Kanishka Pushpitha, Mehdi Mirzaei, Devaraj Basavarajappa, Veer Gupta, Samridhi Sharma, Yuyi You, Giovanna Galliciotti, Ghasem H. Salekdeh, Mark S. Baker, Stuart L. Graham, and Vivek K. Gupta

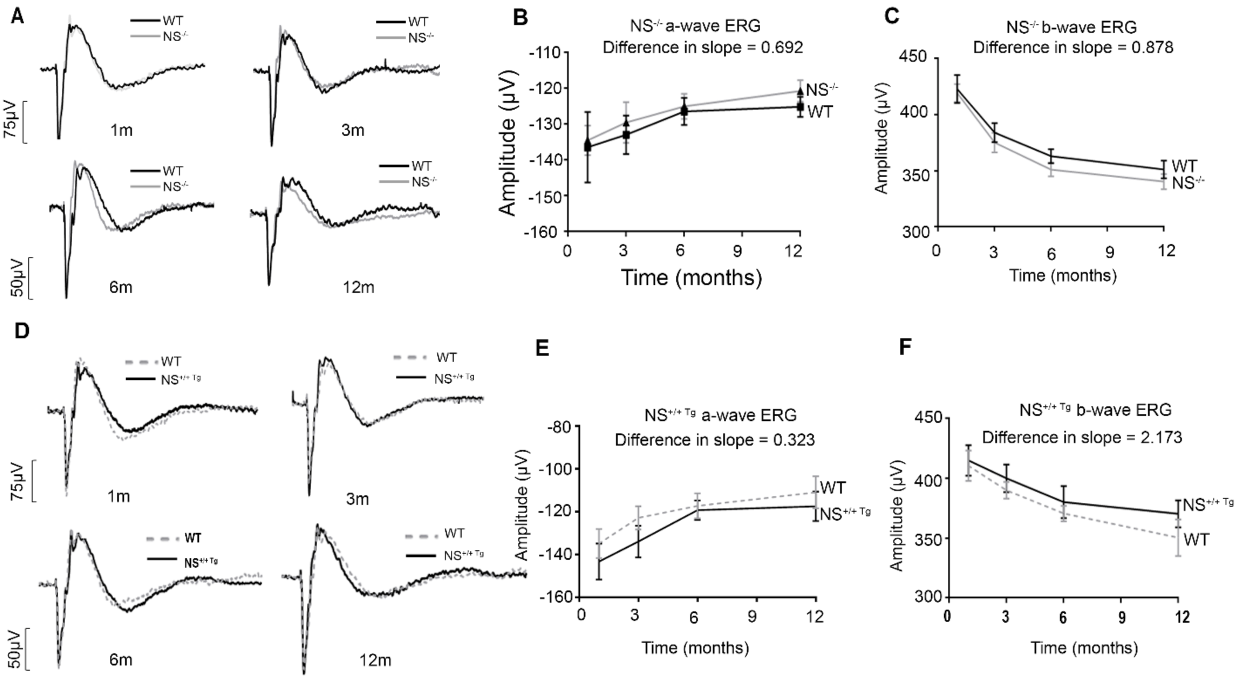

Figure S1 Electroretinogram recordings from $NS^{-/-}$ and $NS^{+/+\ Tg}$ mice. (A) Average ERG trace of WT (black) and $NS^{-/-}$ mice at different time points (1m, 3m, 6m and 12m). Data analyses of ERG (B) a-and (C) b-wave amplitudes revealed no significant differences between the slopes for one-year-old $NS^{-/-}$ mice compared to their age-matched WT. (D) Average ERG trace of WT (dotted grey) and $NS^{+/+\ Tg}$ mice at different time points (1m, 3m, 6m and 12m). Quantification of ERG (E) a-and (F) b-wave amplitudes revealed no significant differences between the slopes for one-year-old $NS^{+/+\ Tg}$ mice compared to their age-matched WT.

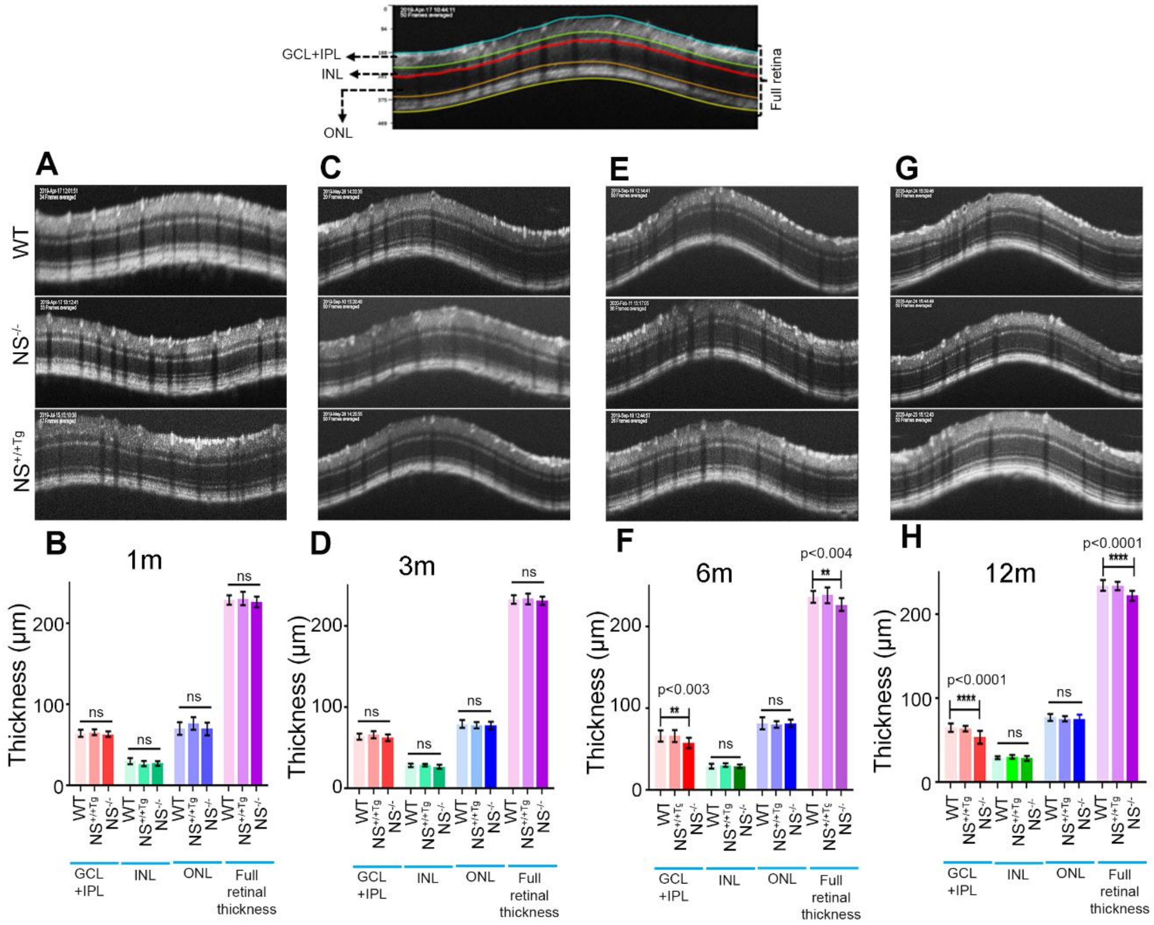

Figure S2 Three distinct layers of GCL+IPL, INL, ONL and whole retinal thickness were segmented, and quantified using Insight software. The averaged thickness of each group was used for comparison between WT, $NS^{-/-}$ and $NS^{+/+Tg}$ groups. (A, C) SD-OCT recording was acquired on a circular scan with a distance of about 0.3 around the optic nerve head in WT, NS-/- and NS+/+Tg mice at one month and three months old. Quantification of retinal layer thickness using Insight software (Phoenix). (B, D) No significant differences were observed in any animal groups in GCL+IPL, INL, ONL and whole retinal thickness at one month and three months. (E) SD-OCT recording was acquired on a circular scan with a distance of about 0.3 around the optic nerve head in WT, $NS^{-/-}$ and $NS^{+/+Tg}$ mice in six-month-old animals. (F) GCL+IPL and whole retinal thickness were significantly reduced in $NS^{-/-}$ mice in six months compared to age match WT and $NS^{+/+Tg}$ mice (n=10 animals in each group, $p<0.003$ and $p<0.004$). (G) SD-OCT recording was acquired on a circular scan with a distance of about 0.3 around the optic nerve head in WT, $NS^{-/-}$ and $NS^{+/+Tg}$ mice in twelve-month-old animals. (H) GCL+IPL and whole retinal thickness were significantly reduced in $NS^{-/-}$ mice in twelve months compared to age match WT and $NS^{+/+Tg}$ mice (n=10 animals in each group, $p<0.0001$ and $p<0.0001$). GCL, ganglion cell layer, IPL, inner plexiform layer; INL, inner nuclear layer; and ONL, outer nuclear layer.

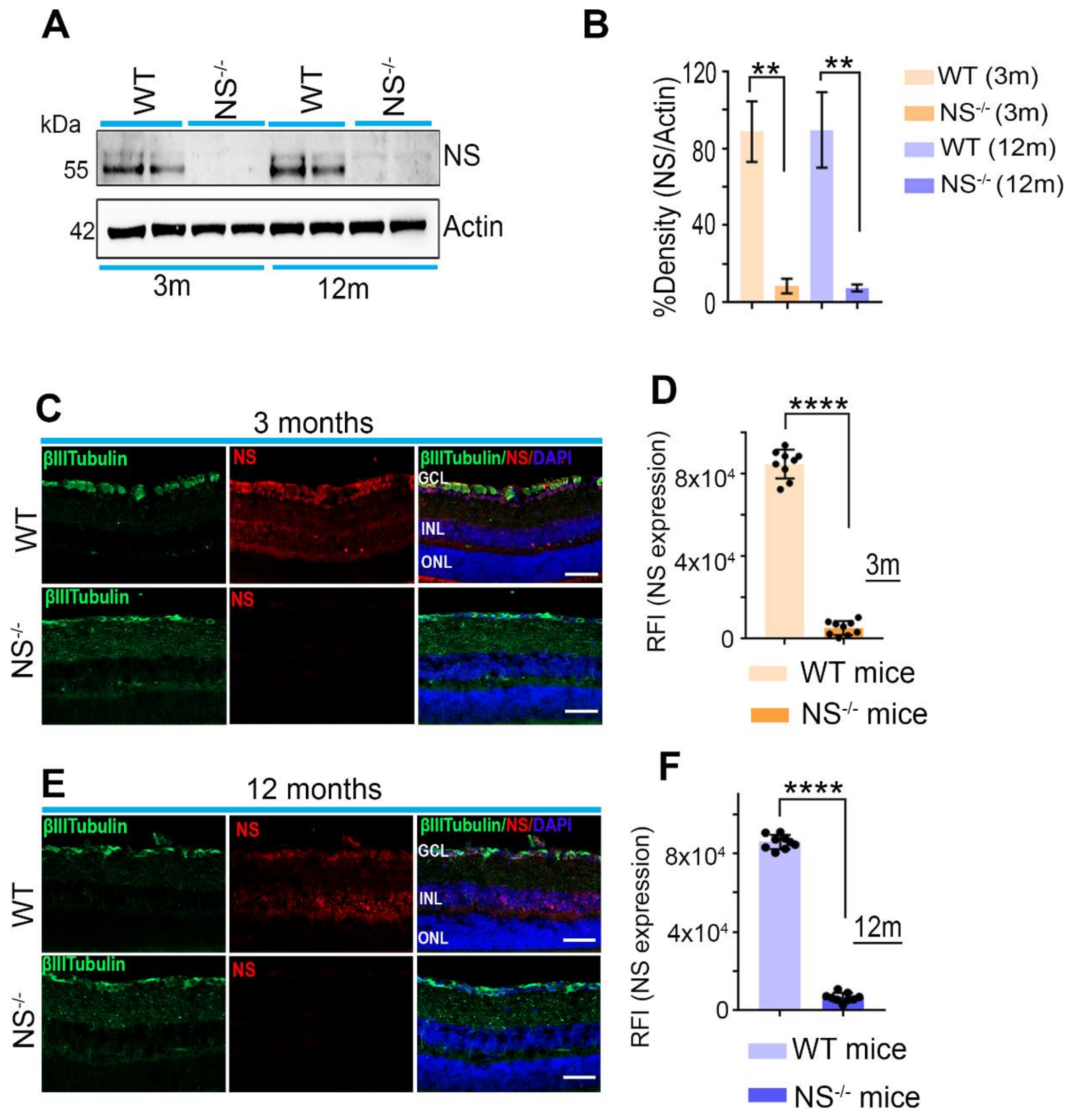

Figure S3 (A) Retinal sections from WT and $NS^{+/+Tg}$ mice stained with DAPI (blue), anti-NS (red), and anti-βIII-tubulin (green) to evaluate expression the expression of NS at young age (3m). (Scale bars=50 µm) (B) Relative fluorescence intensities of neuroserpin immunoreactivity in 3m old WT and $NS^{+/+Tg}$ mice retinal cross-sections were quantified using ImageJ programme in GCL and INL and plotted. (C) Retinal sections from WT and $NS^{+/+Tg}$ mice stained with DAPI (blue), anti-NS (red), and anti-βIII-tubulin (green) to evaluate expression of NS at the old age mice (12m). (Scale bars=50µm). (D) Relative fluorescence intensities of neuroserpin immunoreactivity in 12m old WT and $NS^{+/+Tg}$ mice retinal cross-sections were quantified using ImageJ programme in GCL and INL and plotted. (E) Western blot analysis of neuroserpin (NS) levels in the retinal tissue lysates from WT and $NS^{+/+Tg}$ mice in the young (3m) and old age (12m) in healthy condition (F) Densitometric quantification of the bands indicates significant higher NS expression level in $NS^{+/+Tg}$ mice retina lysates both at young and old age as compared to age matched WT retinas (n = 3 animal in each group; $p < 0.007$, $p<0.003$). Actin was used as loading control. Graphs show means ± SEM and p values obtained using Student's t test.

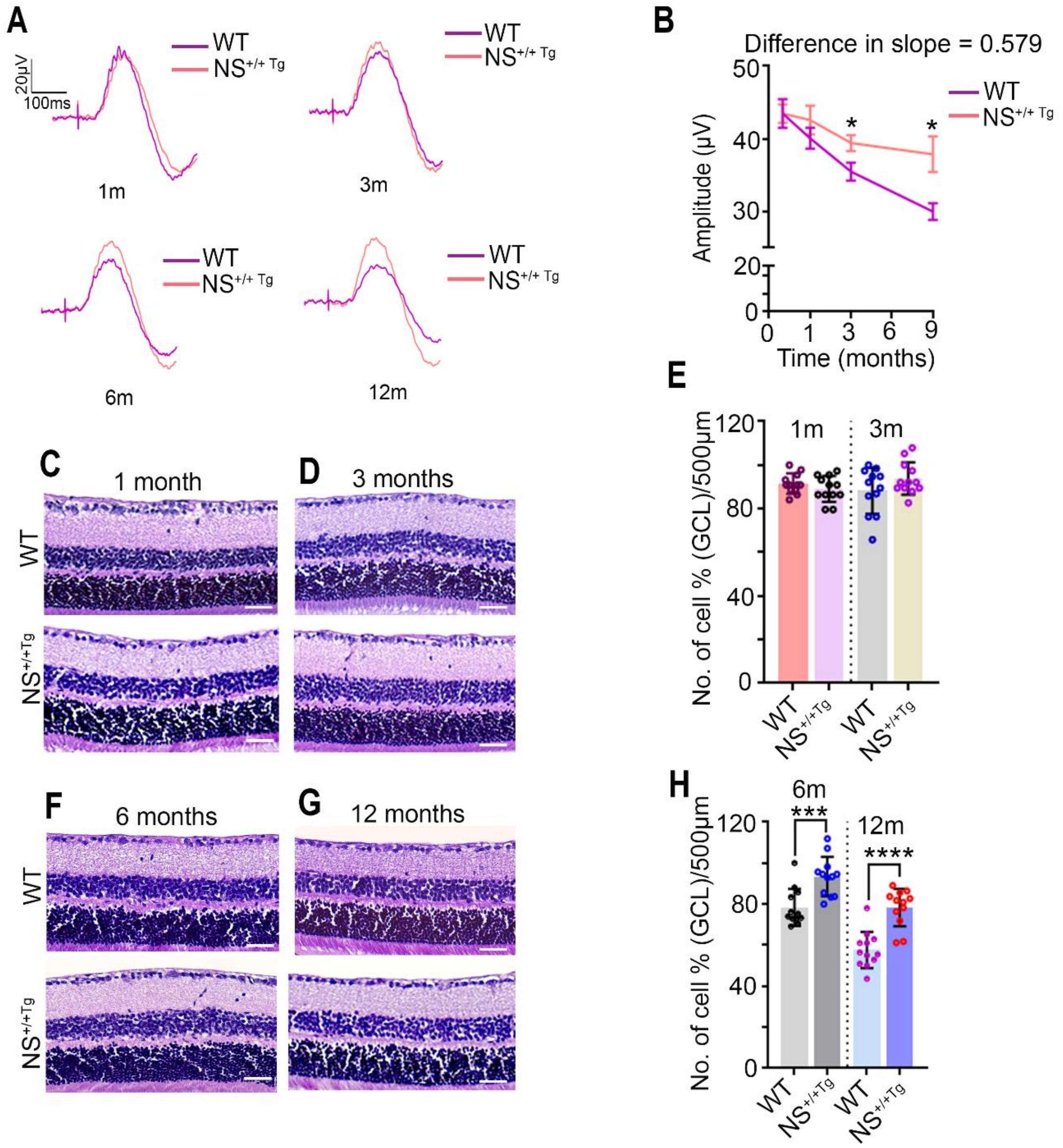

Figure S4 Neuroserpin overexpression protects retinal function and structure against age-related degenerative changes. Retinal structure and function preservation demonstrated in NS$^{+/+Tg}$ mice follow up study for 1 year. (A) Average trace of pSTR signal obtained from WT (magenta) and NS$^{+/+Tg}$ mice (light pink) at different age point at 1 month, 3 months, 6 months and 12 months (B) Slope analysis for pSTR functional parameter. A significant difference in the slope of preservation was only observed in pSTR, suggesting that this parameter alleviated in the NS$^{+/+Tg}$ mice-group starting from 6m and continued to one year (n=10 animals per group, per time-point. Hematoxylin–eosin-stained retinal sections of aged match WT and NS$^{+/+\ Tg}$ mice at (C) 1 month (D) 3 months. (Scale bar=50µm) (E) Quantitative analysis of GCL density has no significant change in NS$^{+/+\ Tg}$ mice following upto three months (n= 4 animal in each group, ns). (F, G) Hematoxylin–eosin-stained retinal sections of aged match WT and NS$^{+/+\ Tg}$ mice at 6 months and 12 months. (Scale bar=50µm) (H) Quantitative analysis of GCL density showed a significant higher GC density in NS$^{+/+\ Tg}$ mice following 6 and 12 months (n=4, p=0.0001 (6m) and p<0.0001 (12m).

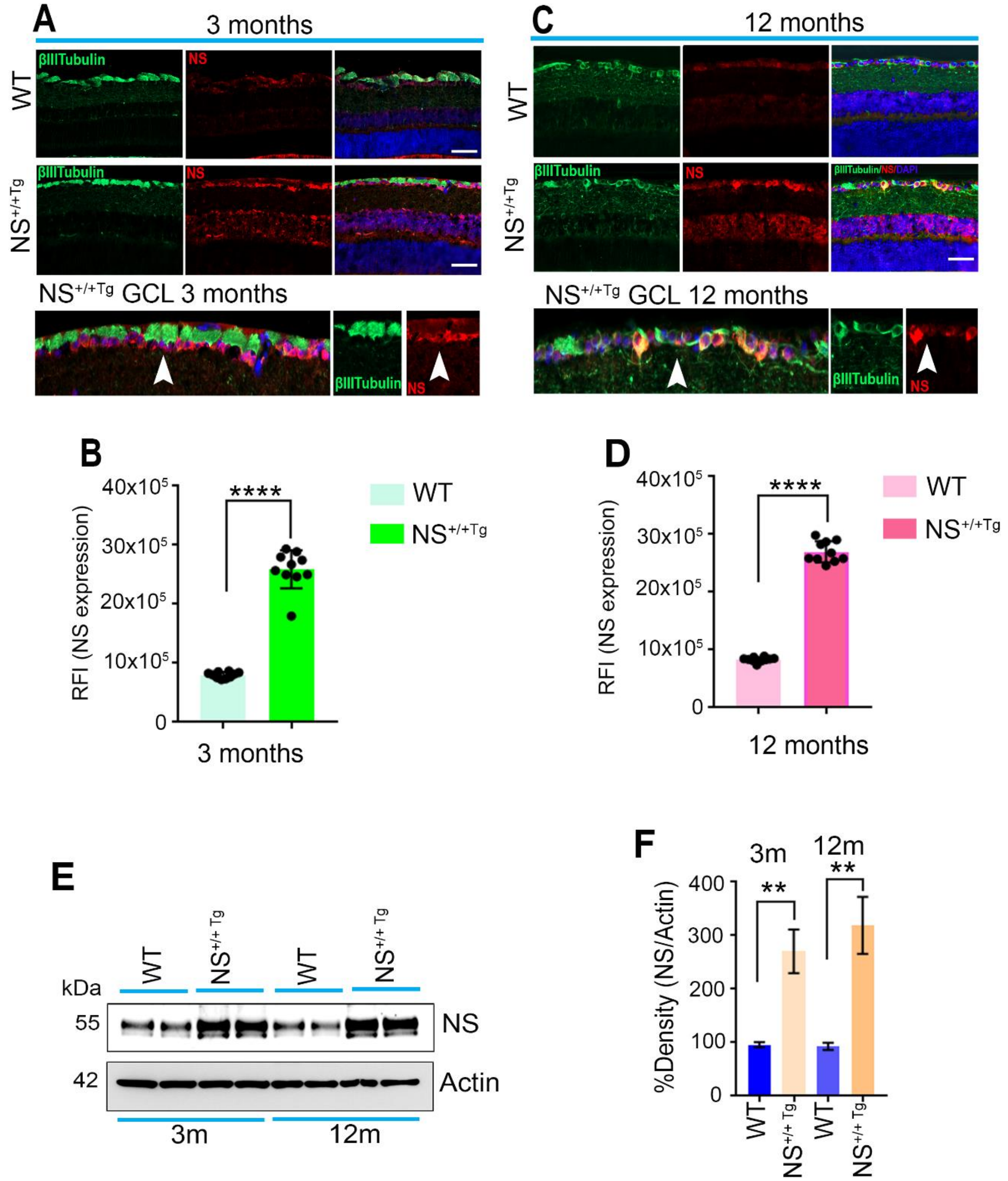

Figure S5 (A) Retinal sections from WT and $NS^{+/+Tg}$ mice stained with DAPI (blue), anti-NS (red), and anti-βIII-tubulin (green) to evaluate expression the expression of NS at young age (3m). (Scale bars=50 µm) (B) Relative fluorescence intensities of neuroserpin immunoreactivity in 3m old WT and $NS^{+/+Tg}$ mice retinal cross-sections were quantified using ImageJ programme in GCL and INL and plotted. (C) Retinal sections from WT and $NS^{+/+Tg}$ mice stained with DAPI (blue), anti-NS (red), and anti-βIII-tubulin (green) to evaluate expression of NS at the old age mice (12m). (Scale bars=50µm). (D) Relative fluorescence intensities of neuroserpin immunoreactivity in 12m old WT and $NS^{+/+Tg}$ mice retinal cross-sections were quantified using ImageJ programme in GCL and INL and plotted. (E) Western blot analysis of neuroserpin (NS) levels in the retinal tissue lysates from WT and $NS^{+/+Tg}$ mice

in the young (3m) and old age (12m) in healthy condition (F) Densitometric quantification of the bands indicates significant higher NS expression level in $NS^{+/+Tg}$ mice retina lysates both at young and old age as compared to age matched WT retinas (n = 3 animal in each group; $p < 0.007$, $p<0.003$). Actin was used as loading control. Graphs show means ± SEM and p values obtained using Student's t test.

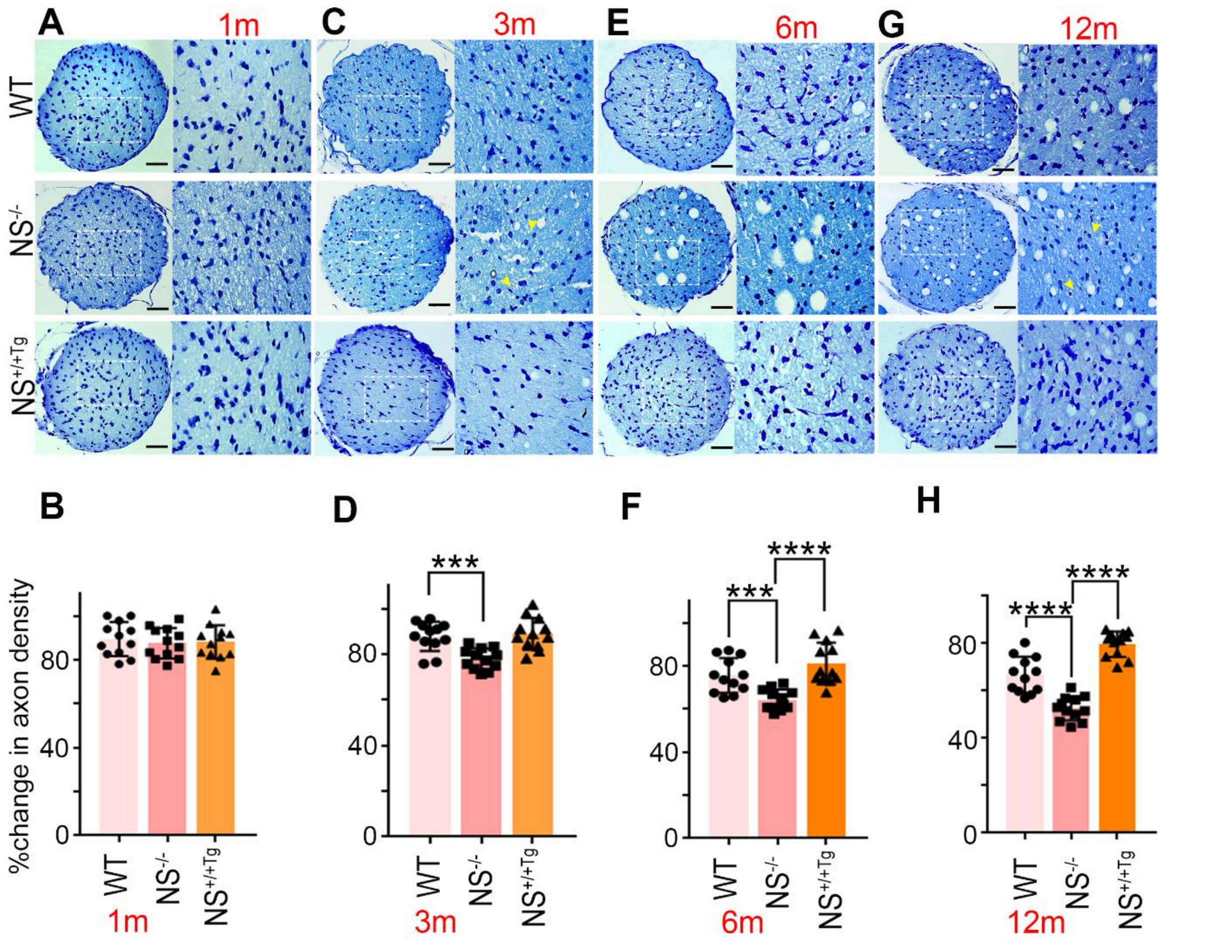

Figure S6 Neuroprotective function of neuroserpin against optic nerve degeneration. Optic nerve axonal appearance in WT, $NS^{-/-}$ and $NS^{+/+\,Tg}$ mice stained with toluidine blue (A) (L) Representative low magnification images of WT, $NS^{-/-}$ and $NS^{+/+Tg}$ mice optic nerve of one month old. (Scale bar=20μm) (R) High magnification of optic nerves corresponding to insets (white box) from the low magnification images (B) Quantification of axonal density revealed no significant axonal loss in any of WT, $NS^{-/-}$ and $NS^{+/+\,Tg}$ mice optic nerve at one month (n=3 animals, 3 sections/ animal) (C) (L) Representative low magnification images of WT, $NS^{-/-}$ and $NS^{+/+Tg}$ mice optic nerve of three-month-old. (Scale bar=20μm) (R) High magnification of optic nerves corresponding to insets (white box) from the low magnification images (D) Quantification of axonal density revealed significant axonal loss in $NS^{-/-}$ mice optic nerve at three months compared to age match WT and $NS^{+/+\,Tg}$ (n=3 animals, 3 sections/ animal, $p<0.0004$) (E) (L) Representative low magnification images of WT, $NS^{-/-}$ and $NS^{+/+\,Tg}$ mice optic nerve of six-month-old. (Scale bar=20μm) (R) High magnification of optic nerves corresponding to insets (white box) from the low magnification images (F) Quantification of axonal density revealed significant axonal loss in $NS^{-/-}$ mice optic nerve at six months compared to age match WT and $NS^{+/+\,Tg}$ (n=3 animals, 3 sections/ animal, $p<0.0005$ and $p<0.0001$). WT mice also demonstrated significant loss of axon density in six-month time when compared to age match $NS^{+/+\,Tg}$ mice optic nerve (n=3 animals, 3 sections/ animal, $p<0.005$) (G) (L) Representative low magnification images of WT, $NS^{-/-}$ and $NS^{+/+Tg}$ mice optic nerve of twelve-month-old. (Scale bar=20μm) (R) High magnification of optic nerves corresponding to insets (white box) from the low magnification images (H) Quantification of axonal density

revealed significant axonal loss in $NS^{-/-}$ mice optic nerve at twelve months compared to age match WT and $NS^{+/+ Tg}$ (n=3 animals, 3 sections/ animal, $p<0.0001$). Significant protection of axon density was observed $NS^{+/+Tg}$ mice optic nerve compared to age match WT mice (n=3 animals, 3 sections/ animal, $p<0.005$). Graphs show means ± SEM and p values obtained using Student's t test.

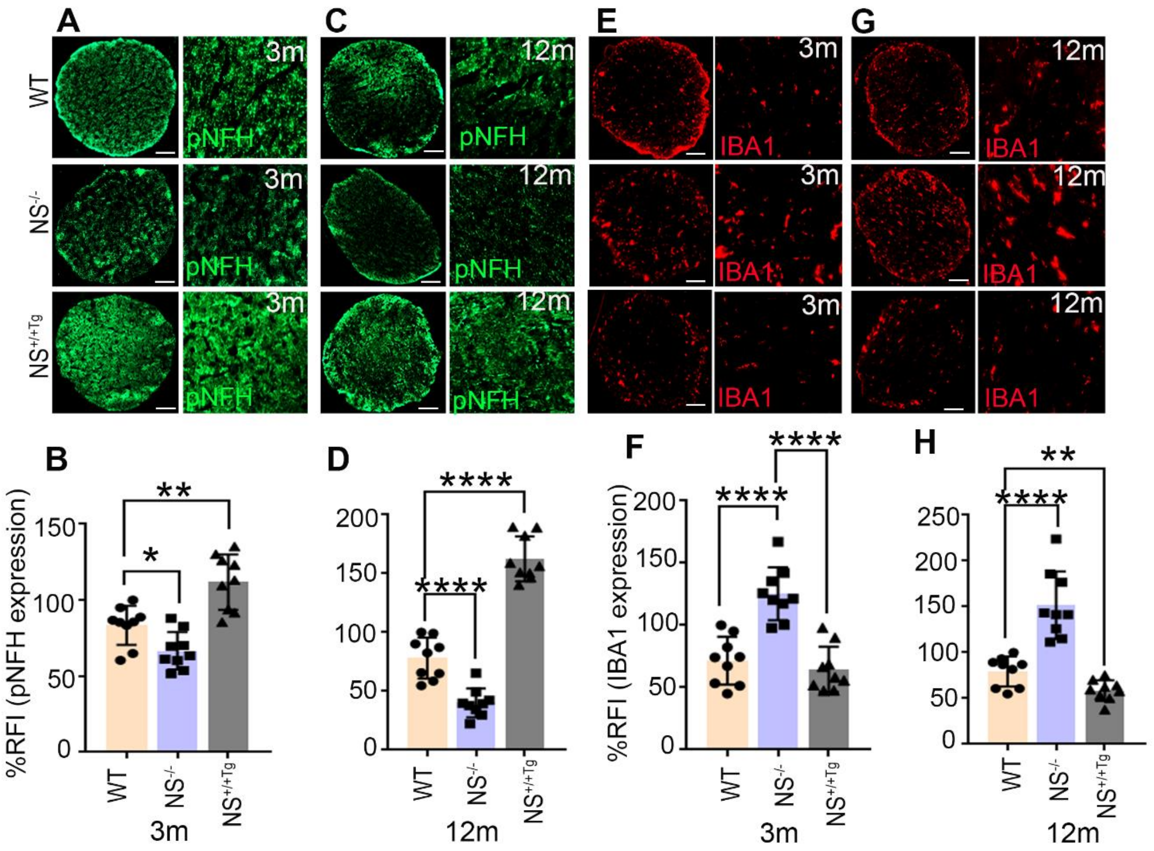

Figure S7 Neuroprotective effects of neuroserpin against optic nerve damage and associated microglial activation. (A) Phosphorylated neurofilament heavy-chain (pNFH, a marker for optic nerve damage) immunoreactivity in the WT, $NS^{-/-}$ and $NS^{+/+\ Tg}$ mice optic nerve section at young age (3m) left panel: representative photomicrographs showing pNFH immunostaining in transverse sections of the proximal portion of the optic nerve. Detailed images are shown in right side panels. Scale bar =20μm. (B) Quantification of the pNFH immunoreactivity in the proximal optic nerve showed significant decline in pNFH immunoreactivity in three-month-old NS-/- mice (n=3 animals, 3 sections/animal, p<0.0003). pNFH immunoreactivity was detectable in the WT control whereas significantly higher pNFH immunoreactivity was measured in three-month old $NS^{+/+\ Tg}$ mice optic nerve (n=3 animals, 3 sections/animal, p<0.0001) (C) Phosphorylated neurofilament heavy-chain (pNFH) immunoreactivity in the WT, $NS^{-/-}$ and $NS^{+/+\ Tg}$ mice optic nerve section at old age (3m) left panel: representative photomicrographs showing pNFH immunostaining in transverse sections of the proximal portion of the optic nerve. Detailed images are shown in right side panels. Scale bar =20μm. (D) Quantification of the pNFH immunoreactivity in the proximal optic nerve showed further significant decline in pNFH immunoreactivity in twelve-month-old $NS^{-/-}$ mice (n=3 animals, 3 sections/animal, p<0.0008). pNFH immunoreactivity was detectable in the WT control and was reduced as compared to three-month age. Higher pNFH immunoreactivity was measured in twelve-month old $NS^{+/+\ Tg}$ mice optic nerve compared to age match WT optic nerve (n=3 animals, 3 sections/animal, p<0.0001) (E) Microglia analysis in the proximal optic nerve portion. Representative photomicrographs showing ionized calcium-binding adaptor molecule 1 (IBA1) immunostaining patterns in cross-sections of optic nerves in the WT, $NS^{-/-}$ and $NS^{+/+\ Tg}$ mice optic nerve section at young age (3m) Scale bar = 20μm. Detailed images are shown

in right panels. (F) Analysis of IBA1 immunoreactivity at three-month time, a significant increase of IBA1 immunoreactivity was observed in $NS^{-/-}$ mice optic nerve compared to age match WT control (n=3 animals, 3 sections/animal, p<0.0001). Interestingly, IBA1 immunoreactivity was detected in $NS^{+/+\ Tg}$ mice optic nerve section but remain significantly low when compared to age match WT optic nerve (n=3 animals, 3 sections/animal, p<0.0001). (G) Representative photomicrographs showing ionized calcium-binding adaptor molecule 1 (IBA1) immunostaining patterns in cross-sections of optic nerves in the WT, $NS^{-/-}$ and $NS^{+/+\ Tg}$ mice optic nerve section at old age (12m) Scale bar = 20μm. Detailed images are shown in right panels. (H) Analysis of IBA1 immunoreactivity at twelve-month time, a significant increase of IBA1 immunoreactivity was observed in $NS^{-/-}$ mice optic nerve compared to age match WT control (n=3 animals, 3 sections/animal, p<0.0001). IBA1 immunoreactivity was detected in $NS^{+/+\ Tg}$ mice optic nerve section but remain significantly low when compared to age match WT optic nerve (n=3 animals, 3 sections/animal, p<0.0001). Graphs show means ± SEM and p values obtained using Student's t test.

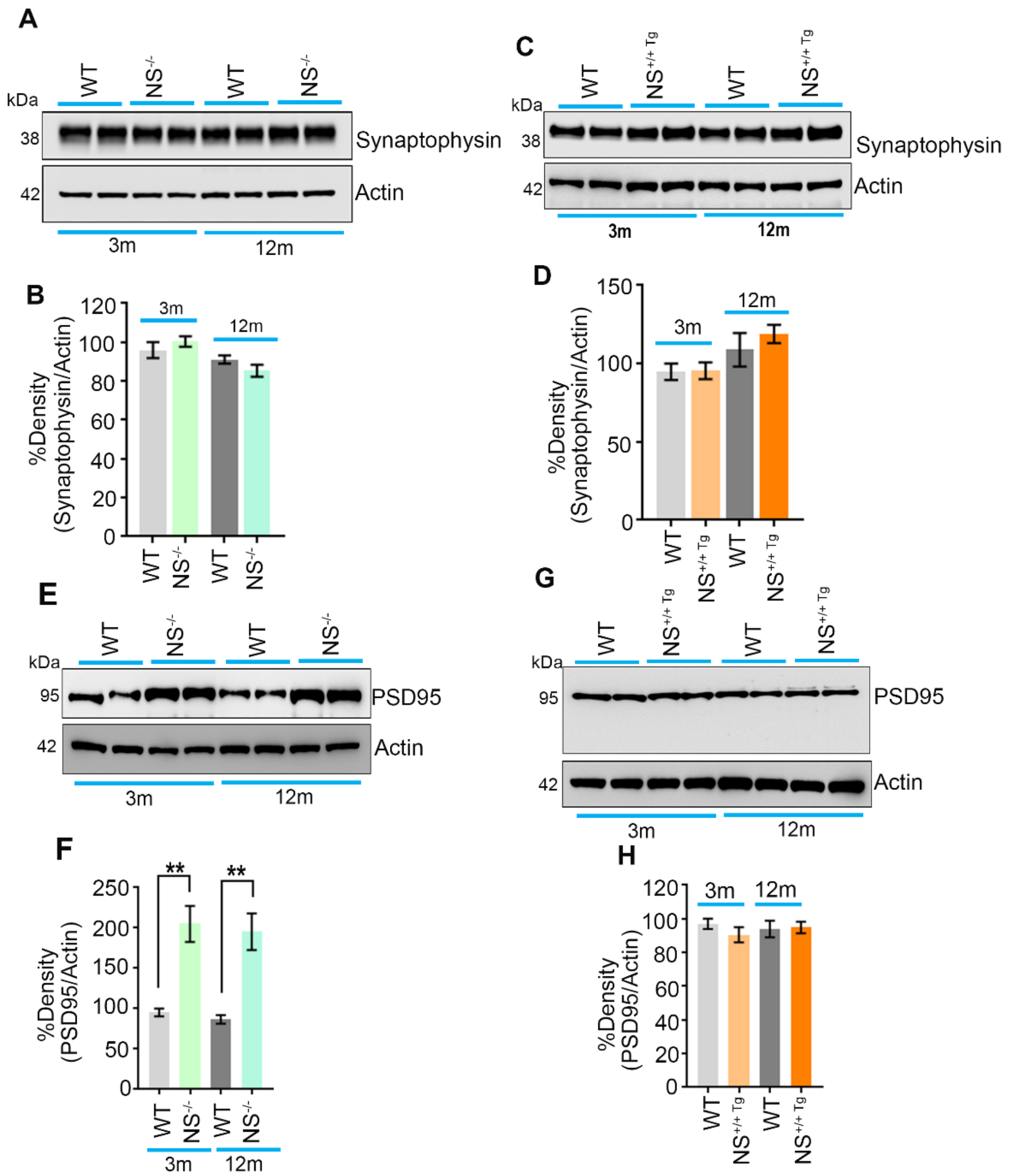

Figure S8 Increased expression of the synaptic protein PSD95 and unaltered expression of synaptophysin, beclin-1 and LC3 II/I in neuroserpin-deficient mice. (A) Representative Western blots of retinal extracts from WT and $NS^{-/-}$ animals at 3m and 12m separated by SDS-PAGE and analyzed with antibodies against synaptophysin (B) Quantification revealed no change in percentage density of synaptophysin (C) Representative Western blots of retinal extracts from WT and $NS^{+/+Tg}$ animals at 3m, and 12m separated by SDS-PAGE and analyzed with antibodies against synaptophysin (D) Quantification revealed no change in percentage density of synaptophysin (E) Representative Western blots of retinal extracts from WT and $NS^{-/-}$ animals at 3m and 12m separated by SDS-PAGE and analyzed with antibodies against PSD95 (F) Quantification revealed a significant increase in percentage density of PSD95 in 3m and 12m $NS^{-/-}$ mice retina ($p<0.0093$). Band intensity was normalized to β-actin expression. (G) Representative Western blots of retinal extracts from WT and $NS^{+/+Tg}$ animals at 3m and 12m separated by SDS-PAGE and analyzed with antibodies against PSD95 (H) Quantification revealed no significant change in PSD95 expression in 3m and 12m $NS^{+/+Tg}$ mice retina. Band intensity was normalized to β-actin expression.

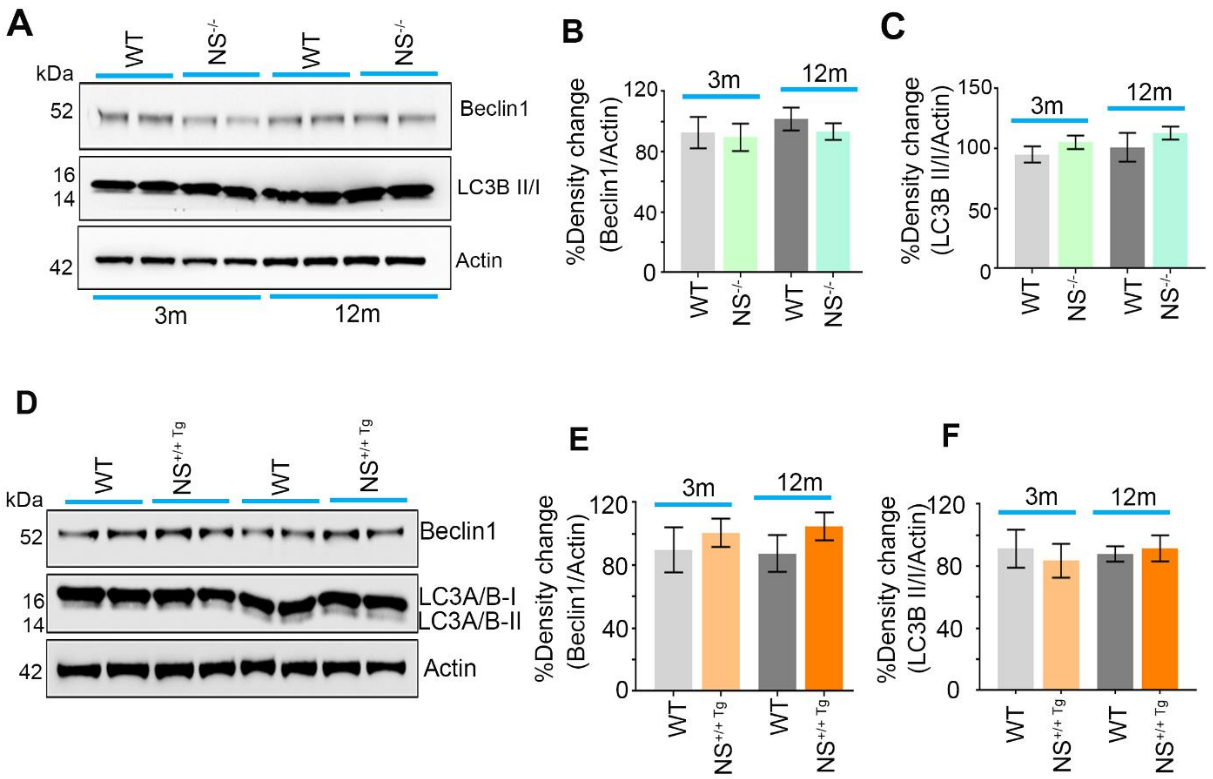

Figure S9 Expression of autophagy markers in young and old neuroserpin ablation ($NS^{-/-}$) and neuroserpin-overexpression ($NS^{+/+\ Tg}$) mice. (A and D) Representative Western blots of retinal extracts from different animals at 3m and 12m were separated by SDS-PAGE and analyzed with antibodies against Beclin1 and LC3B II/I. Band intensity was normalized to β-actin expression. (B, C and E, F) Quantification revealed no change in percentage density of synaptophysin, PSD95, Beclin1 and LC3B II/I at any time.

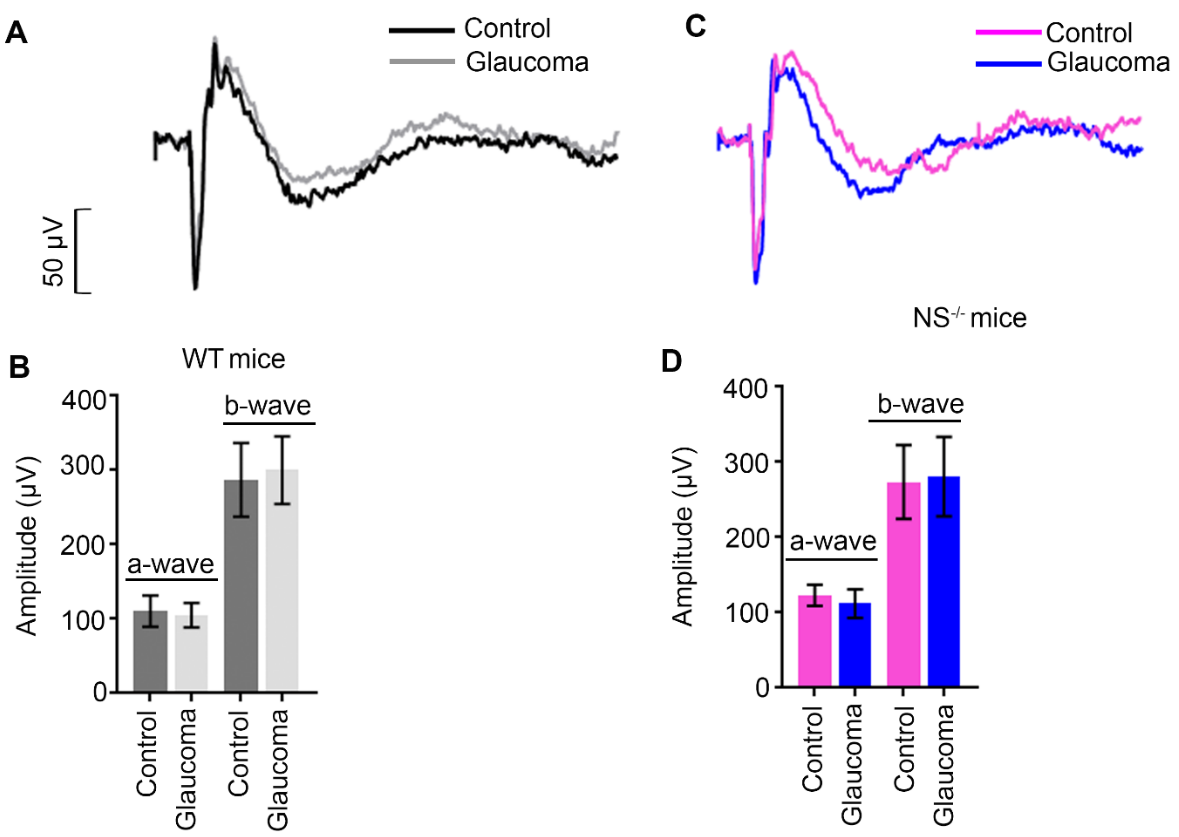

Figure S10 (A) Average ERG traces of WT mice in normal and glaucoma. (B) Data analyses of ERG a- and b-wave amplitudes revealed no significant differences between the amplitudes of normal and glaucoma conditions in WT mice. (C) Average ERG traces of $NS^{-/-}$ mice in normal and glaucoma. (D) Data analyses of ERG a- and b-wave amplitudes showed no significant differences between the control and glaucoma condition amplitudes in $NS^{-/-}$ mice.

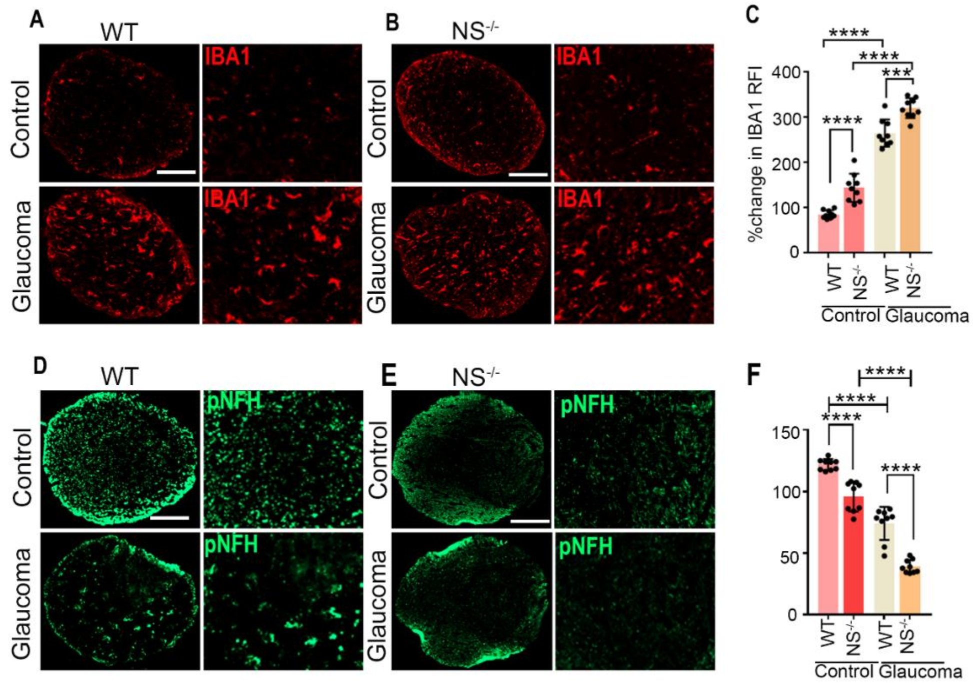

Figure S11 Neuroserpin deficiency increases optic nerve damage and microglial activation in glaucomatous conditions (A) Microglia analysis in the proximal optic nerve portion. Representative photomicrographs showing ionized calcium-binding adaptor molecule 1 (IBA1, marker for microglia) immunostaining patterns in cross-sections of optic nerves from non-treated control and microbead injected eyes in the WT mice. Scale bar =20μm. Detailed images are shown in right panels. (B) Representative photomicrographs showing IBA1 immunostaining patterns in cross-sections of optic nerves from non-treated control and microbead injected eyes in the NS$^{-/-}$ mice. Scale bar = 20μm. Detailed images are shown in right panels. (C) Analysis of IBA1 immunoreactivity at 8 weeks of ocular hypertension, a significant increase of IBA1 immunoreactivity was observed in microbead WT retinas (n=4 animals, 3 sections/ animal, p<0.0001). IBA1 immunoreactivity was detectable in the WT control, whereas NS$^{-/-}$ mice in normal and high IOP induced a significant increase in this parameter at 8 weeks (n=4 animals, 3 sections/ animal, p<0.0001 and p<0.009). (D) Phosphorylated neurofilament heavy-chain (pNFH) immunoreactivity in the WT mice in healthy and glaucoma condition, left panel: representative photomicrographs showing pNFH immunostaining in transverse sections of the proximal portion of the optic nerve. Detailed images are shown in right side panels. Scale bar = 20μm. (E) pNFH immunoreactivity in the NS$^{-/-}$ mice in normal and glaucoma condition, left panel: representative photomicrographs showing pNFH immunostaining in transverse sections of the proximal portion of the optic nerve. Detailed images are shown in right side panels. Scale bar = 20μm. (F) Quantification of the pNFH immunoreactivity in the proximal optic nerve, microbead injections following 8 weeks induced a significant decrease in pNFH immunoreactivity in WT glaucoma (n=4 animals, 3 sections/ animal, p<0.008). pNFH immunoreactivity was detectable in the WT control, whereas NS$^{-/-}$ mice in normal and high IOP induced a significant decrease in this parameter at 8 weeks (n=4 animals, 3 sections/ animal, p<0.0001, p<0.0009). Graphs show means ± SEM and p values obtained using Student's t test.

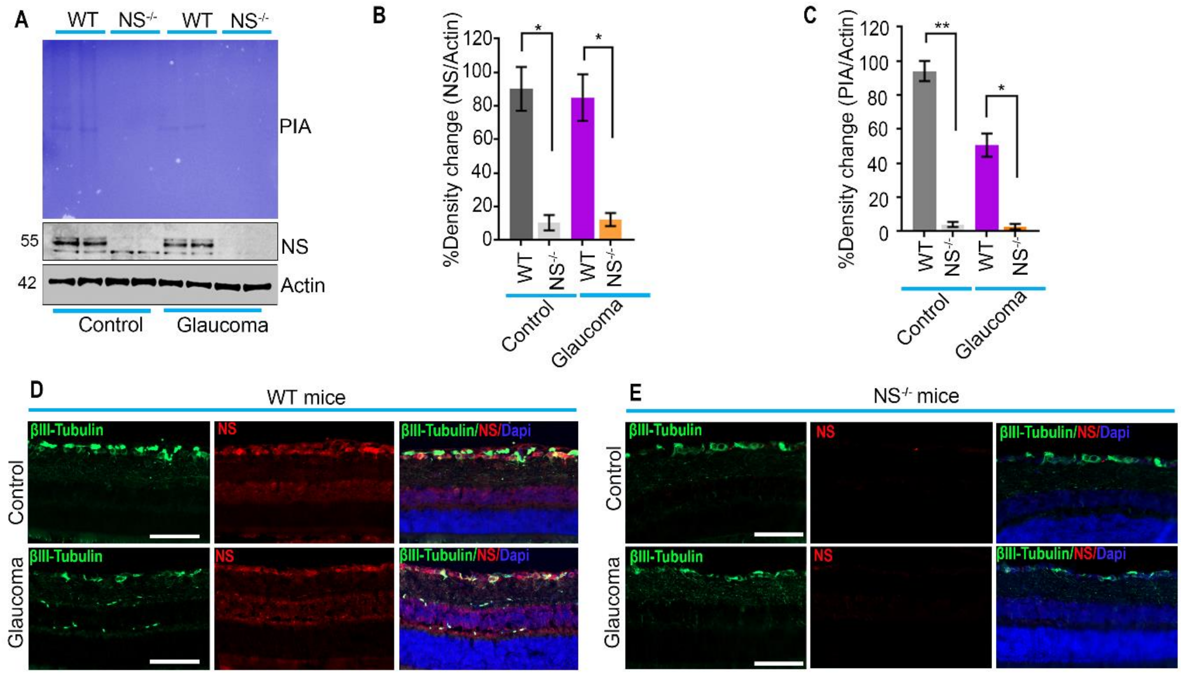

Figure S12 (A) Neuroserpin from WT and NS$^{-/-}$ mice in control and high IOP ONH were subjected to gelatin gel zymography to assess its plasmin inhibitory activity (PIA) (n = 3). Retina lysates were also loaded for western blotting and developed for neuroserpin immunoreactivity in each case. Blots were cropped to show the relevant band. Actin was used as a loading control (B) Densitometric Quantification of the neuroserpin band intensity was significantly lowered in NS$^{-/-}$ mice ONH compared to WT mice in both control and experimental glaucoma condition ($p<0.04$, $p<0.03$) (C) Relative band intensities were quantified, and data analysis indicated significantly decreased plasmin inhibitory activity in WT mice ONH subjected to high IOP ($p<0.05$), however significantly lower PIA activity was measured in NS$^{-/-}$ in control and glaucoma condition compared to the respective controls ($p<0.004$, $p<0.02$) (D, E) Retinal sections from WT and NS$^{-/-}$ mice were stained with DAPI (blue), anti-NS (red), and anti-βIII-tubulin (green) to evaluate the expression of NS and βIII-tubulin in WT mice (D) and NS$^{-/-}$ mice (E) in control and glaucoma condition. Scale bars, 50 μm.

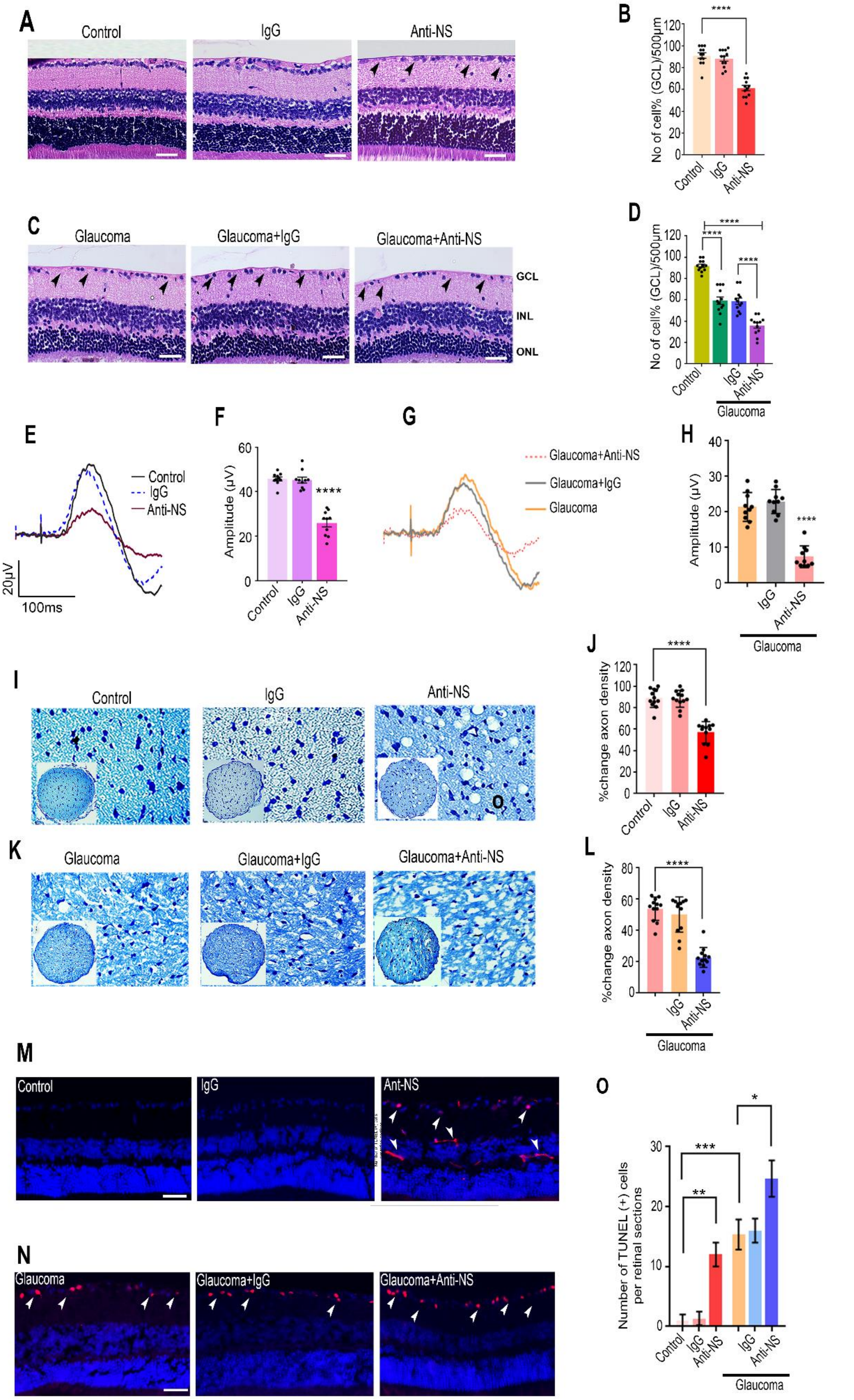
A
Control
IgG
Anti-NS
B
****
No of cell% (GCL)/500μm
0
20
40
60
80
100
120
Control
IgG
Anti-NS
C
Glaucoma
Glaucoma+IgG
Glaucoma+Anti-NS
GCL
INL
ONL
D
****
No of cell% (GCL)/500μm
0
20
40
60
80
100
120
Control
IgG
Anti-NS
Glaucoma
E
Control
IgG
Anti-NS
20μV
100ms
F
Amplitude (μV)
0
20
40
60
****
Control
IgG
Anti-NS
G
Glaucoma+Anti-NS
Glaucoma+IgG
Glaucoma
H
Amplitude (μV)
0
10
20
30
40
****
IgG
Anti-NS
Glaucoma
I
Control
IgG
Anti-NS
J
****
%change axon density
0
20
40
60
80
100
120
Control
IgG
Anti-NS
K
Glaucoma
Glaucoma+IgG
Glaucoma+Anti-NS
L
****
%change axon density
0
20
40
60
80
IgG
Anti-NS
Glaucoma
M
Control
IgG
Ant-NS
N
Glaucoma
Glaucoma+IgG
Glaucoma+Anti-NS
O
*
***
**
Number of TUNEL (+) cells per retinal sections
0
10
20
30
Control
IgG
Anti-NS
IgG
Anti-NS
Glaucoma

Figure S13 Neuroserpin neutralization in the eyes causes retinal and optic nerve damage in healthy and glaucomatous conditions. Analysis of retinal structural and functional damage in WT mice treated with anti-NS (A) H and E staining of the retinal sections indicating changes in the GCL density. (Scale bar=50μm) (B) Quantification (H and E) indicating significant differences in the GCL density in anti-NS treated mice compared to untreated and IgG controls (n= 4 animals, 3 sections/ animal; $p<0.0001$). (C) H and E staining of the retinal sections indicating changes in the GCL density in glaucoma, glaucoma+IgG and glaucoma+anti-NS treated mice retinas. White arrowhead in GCL layer demonstrate ganglion cell degeneration. Scale=50μm (D) Quantification (H and E) indicating significant differences was observed in the GCL density in experimental induce high IOP mice glaucoma (n= 4 animals, 3 sections/ animal; $p<0.0006$, $p<0.0001$) (E) Average trace of pSTR signal obtained from control (black), IgG (blue) and anti-NS (red) mice (F) Quantification indicates a significantly lowered amplitude of pSTR in the anti-NS injected mice compared to the IgG and control ones (n=10 animals in each group; $p<0.0001$) (G) Average trace of pSTR signal obtained high IOP (orange), glaucoma+IgG (grey), and glaucoma+anti-NS (red dotted) mice (H) Quantification indicates a significantly lowered amplitude of pSTR in the high IOP+anti-NS treatment compared to IgG control counterpart (n=10 animals in each group; $p<0.0001$) (I) Cross sections of optic nerve from control, IgG and anti-NS treated animals counterstained with toluidine blue. (Scale bars=20μm) (J) Quantification (toluidine blue) indicating significant decline in the axonal density in anti-NS mice compared to control and IgG (n= 4 animals, 3 sections/ animal; $p<0.001$) (K) Cross sections of optic nerve with or without high IOP, IgG and ant-NS treatment counterstained with toluidine blue. (Scale bars=20μm) (L) Quantification (toluidine blue) indicating significant differences was observed in the axonal density in glaucoma compared to control (n= 4 animals, 3 sections/ animal; $p<0.0001$) and glaucoma+anti-NS compared to glaucoma+IgG (n= 4 animals, 3 sections/ animal, $p<0.0001$). (M) Increased TUNEL-positive staining (red) was observed in retinal sections treated with anti-NS and (N) exposed to microbead injections in WT mice in the inner retinal layers (white arrows). DAPI-stained cell nuclei (blue). (Scale bars=50μm) (O) Quantification of TUNEL-positive cells showing significantly increased number in retinas exposed to anti-NS (n=3 animals in each group, $p<0.002$). Experimental glaucoma significantly increases the TUNEL-positive cells in IgG and anti-NS treatment (n=3 animals in each group, $p<0.0002$, $p<0.0003$). Graphs show means ± SEM and p values obtained using Student's t test.

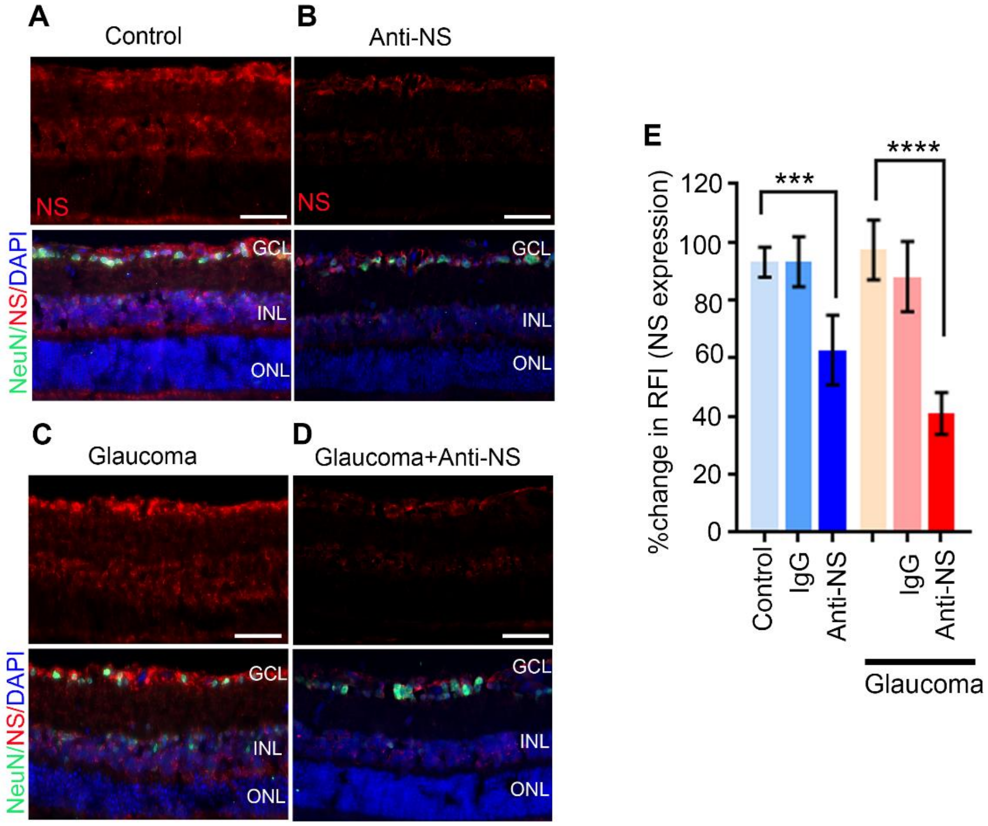

Figure S14 Immunofluorescence retinal images stained with anti-NeuN (green), anti-NS (red) and DAPI (blue) from (A) control (B) anti-NS (C) glaucoma and (D) glaucoma+anti-NS treated retinal sections and compared with non-treated control. Scale bar=50µm (E) Relative fluorescence intensity (RFI) showed a significant decline in neuroserpin immunoreactivity under the neuroserpin neutralization paradigm in healthy and glaucoma conditions. However, no change in neuroserpin RFI was noted in control, IgG, glaucoma and glaucoma+IgG treated with retinas. RFI was quantified and plotted using the ImageJ programme.

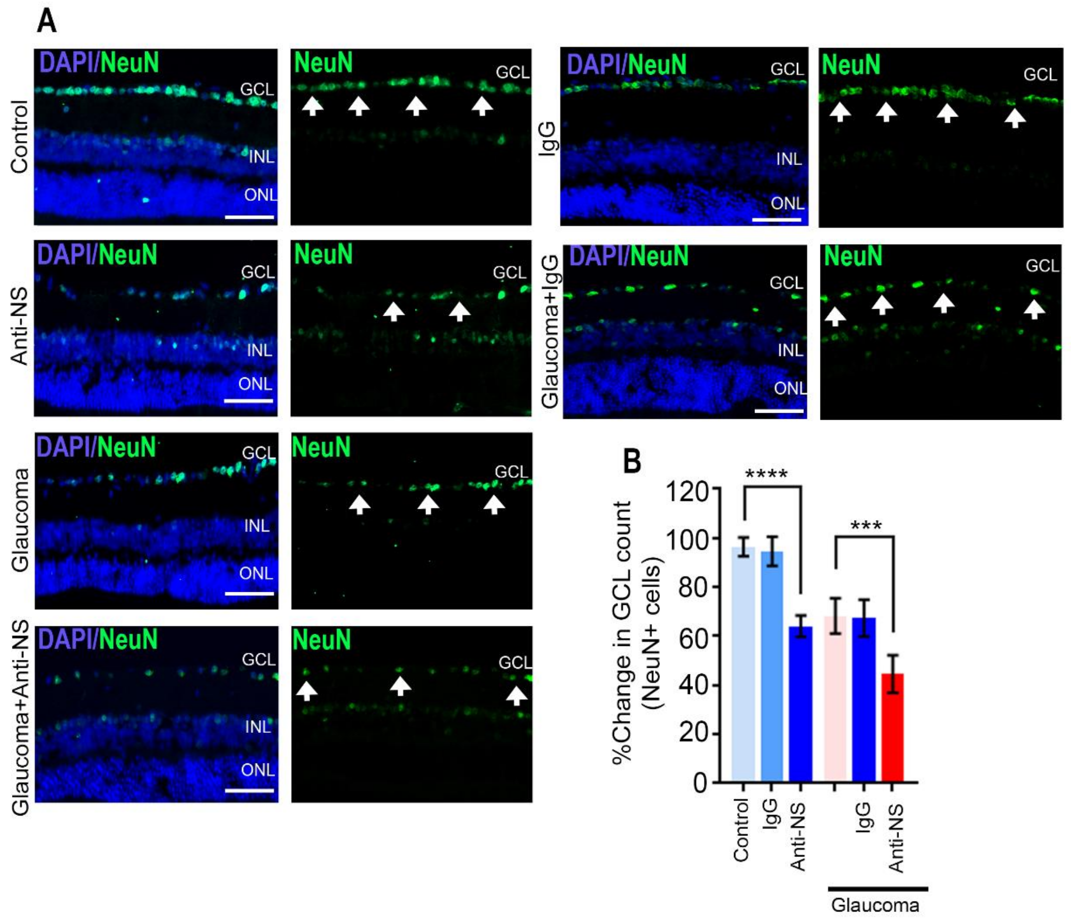

Figure S15 (A) Immunofluorescence images representating control, IgG, anti-NS, glaucoma, glaucoma+IgG and glaucoma+anti-NS sections stained with anti-NeuN (green). Nuclei were counterstained with DAPI (blue). Scale bar=50 µm. (B) Bar graph illustrating the number of NeuN positive (+) cells in the GCL and IPL retina of control, IgG, anti-NS, glaucoma, glaucoma+IgG and glaucoma+anti-NS retinas after two months of either antibody intravitreal injection or microbead alone/ microbead+antibody injections (n=4 animals in each group, $p<0.002$, and $p<0.003$; n=3). GCL, ganglion cell layer; IPL, inner plexiform layer; INL, inner nuclear layer; ONL, outer nuclear layer.

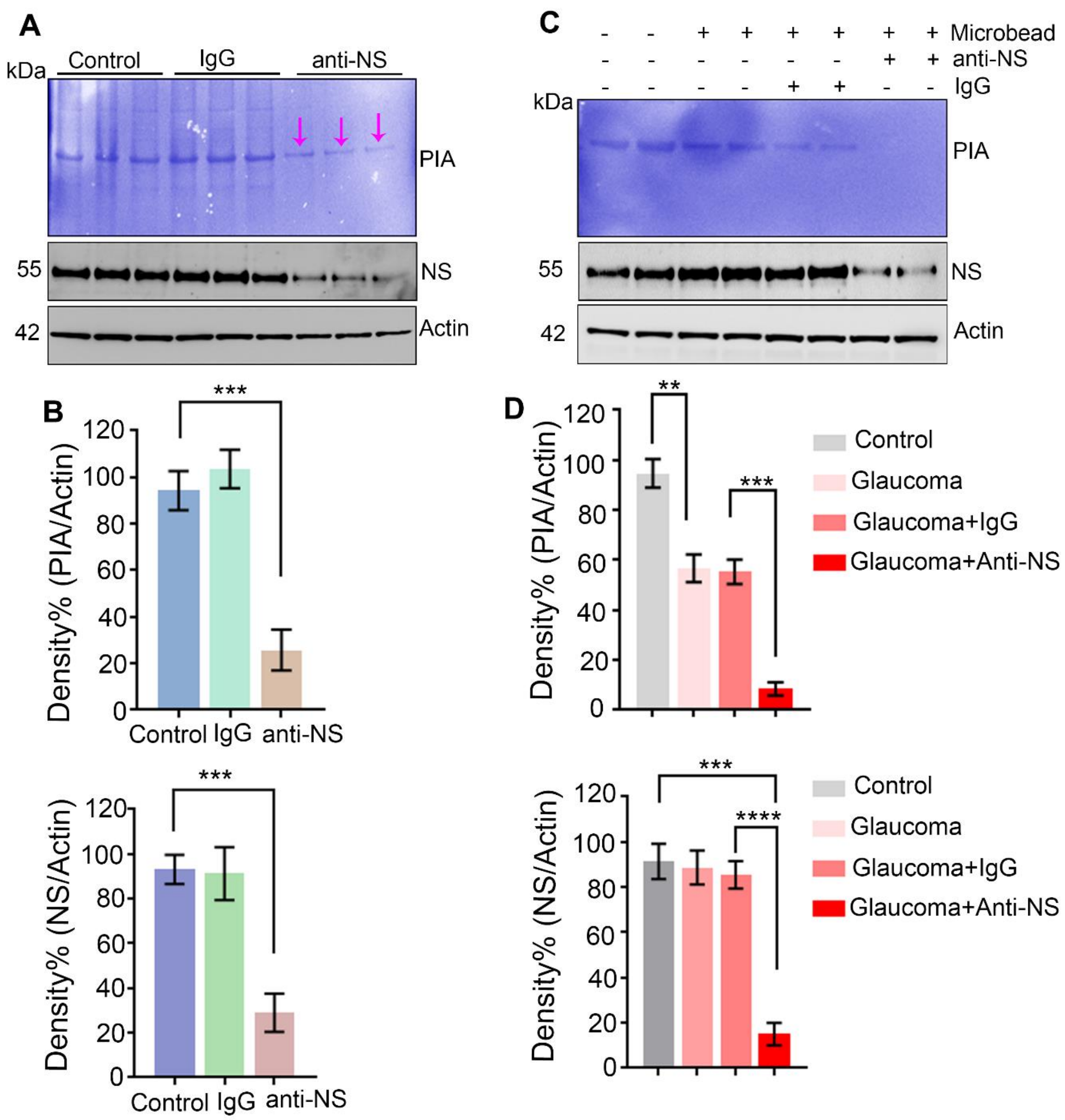

Figure S16 Loss of plasmin inhibitory activity upon neuroserpin neutralization (A) Retinal lysates from control, IgG and anti-neuroserpin antibody were subjected to gelatin zymography to evaluate the inhibitory activity of the neuroserpin and also loaded for western blotting and developed for neuroserpin immunoreactivity in each case (n = 3 each). Actin was used as a loading control (B) Relative band intensities were quantified, and data analysis indicated significantly decreased plasmin inhibitory activity in animal retina subjected to anti-NS treatment compared to IgG and non-treated control ($p<0.0007$, n=3 animals in each group). Neuroserpin expression also significantly decreased upon neuroserpin neutralization ($p < 0.0006$; n=3 animals in each group). (C) retinal lysates from control, microbead, microbead+IgG and microbead+anti-neuroserpin antibody were subjected to gelatin zymography to evaluate the inhibitory activity of the neuroserpin and also loaded for western blotting and developed for neuroserpin immunoreactivity in each case (n = 3 each). Actin was used as a loading control (D) Relative band intensities were quantified, and data analysis indicated significantly decreased plasmin inhibitory activity in both microbead and

microbead+IgG samples and further loss of PIA in microbead+anti-NS retina samples compared to the respective controls ($p<0.002$, $p<0.005$; n=3 animals in each group). No change in neuroserpin expression was observed under microbead or microbead+IgG retinal samples. However, a significant decline in neuroserpin expression was noted upon neuroserpin neutralization in mice retina induced to high IOP ($p<0.0001$; n=3 animals in each group).

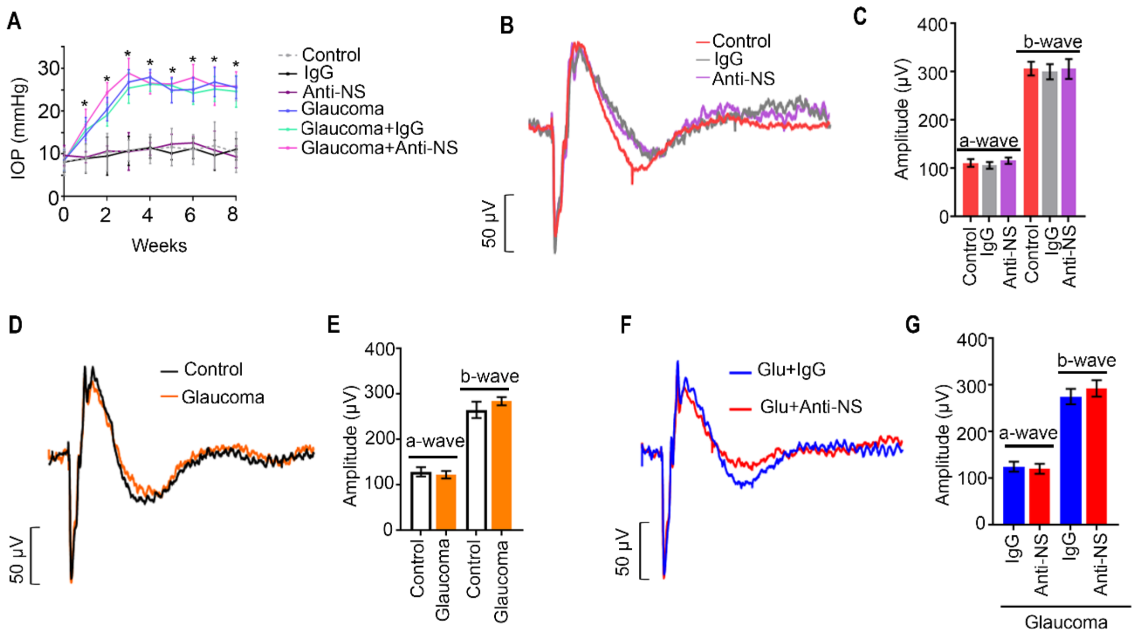

Figure S17 (A) Control, IgG and anti-NS injected mice IOP measurements (10.49±0.40, 10.09±0.38, 10.7±0.40, 22.28±2.21, 21.64±2.04, 23.43±2.20) (n=10/ group). Weekly injections of microbeads, microbeads+IgG and microbeads+anti-NS induced an elevation of IOP and were maintained for eight weeks. (B) Average ERG trace of control (red), IgG (grey), and anti-NS (magenta) treated eyes (C) Data analyses of ERG a- and b-wave amplitudes revealed no significant differences between the groups (D) Average ERG traces of control (black) and glaucoma (orange) (E) Data analyses of ERG a- and b-wave amplitudes revealed no significant differences between the two groups (F) Average ERG traces of glaucoma+IgG (blue) and glaucoma+anti-NS (red) (G) Quantification of ERG a- and b-wave amplitudes revealed no significant differences between the IgG and anti-NS treated groups in experimental glaucoma conditions.

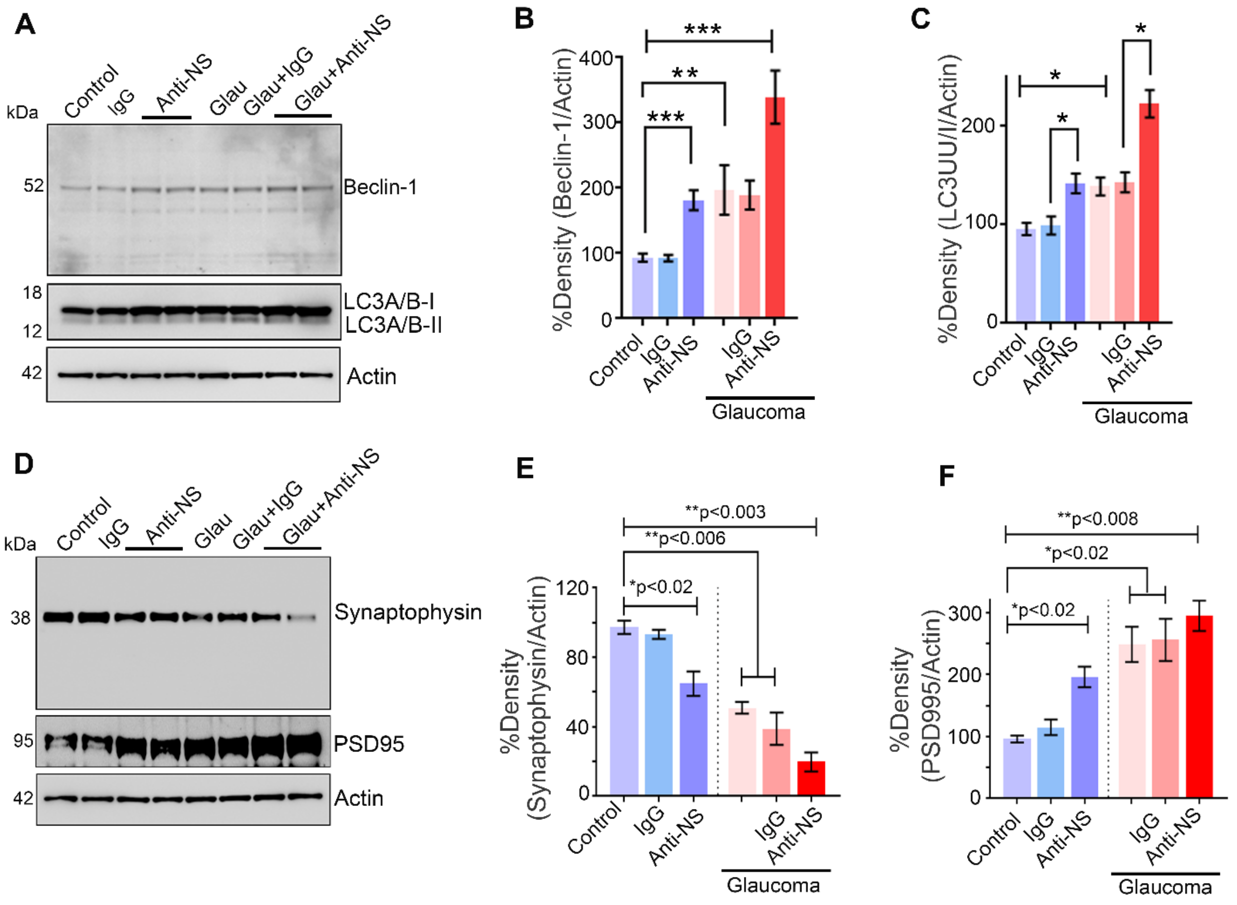

Figure S18 Effects of neuroserpin neutralization on autophagy and synaptic marker changes in the control and high IOP retinas (A) Western blot analysis of Beclin-1 and LC3B-II/LC3B-I levels. β-actin was used as a control, and changes in the microbead, microbead +IgG and microbead + anti-NS retinas relative to control were evaluated. (B) Immunoreactivity of Beclin-1 showed a significant increase in retinas treated with anti-NS, and it was further upregulated when glaucomatous eyes were subjected to NS neutralization ($p<0.02$, $p<0.03$ and $p<0.014$; n=3 each group) (C) LC3-II/ -I levels showed a significant increase in anti-NS treated mice when compared to control conditions (n=3 animals /group, $p<0.03$ and $p<0.007$) (D) Synaptophysin and PSD95 synaptic markers from control, IgG, anti-NS, microbead, microbead+IgG and microbead+anti-NS treated mice retinas relative to control were evaluated. Actin was used as a loading control. (E) A significant decrease in synaptophysin expression levels was observed upon NS neutralization in both control and experimental glaucoma conditions ($p<0.02$, $p<0.006$ and $p<0.003$; n=3 / group) (F) PSD95 showed a significant increase in retinas treated with anti-NS and these levels were further elevated in eyes subjected to a glaucomatous injury ($p<0.02$, and $p<0.008$; n=3 / group).

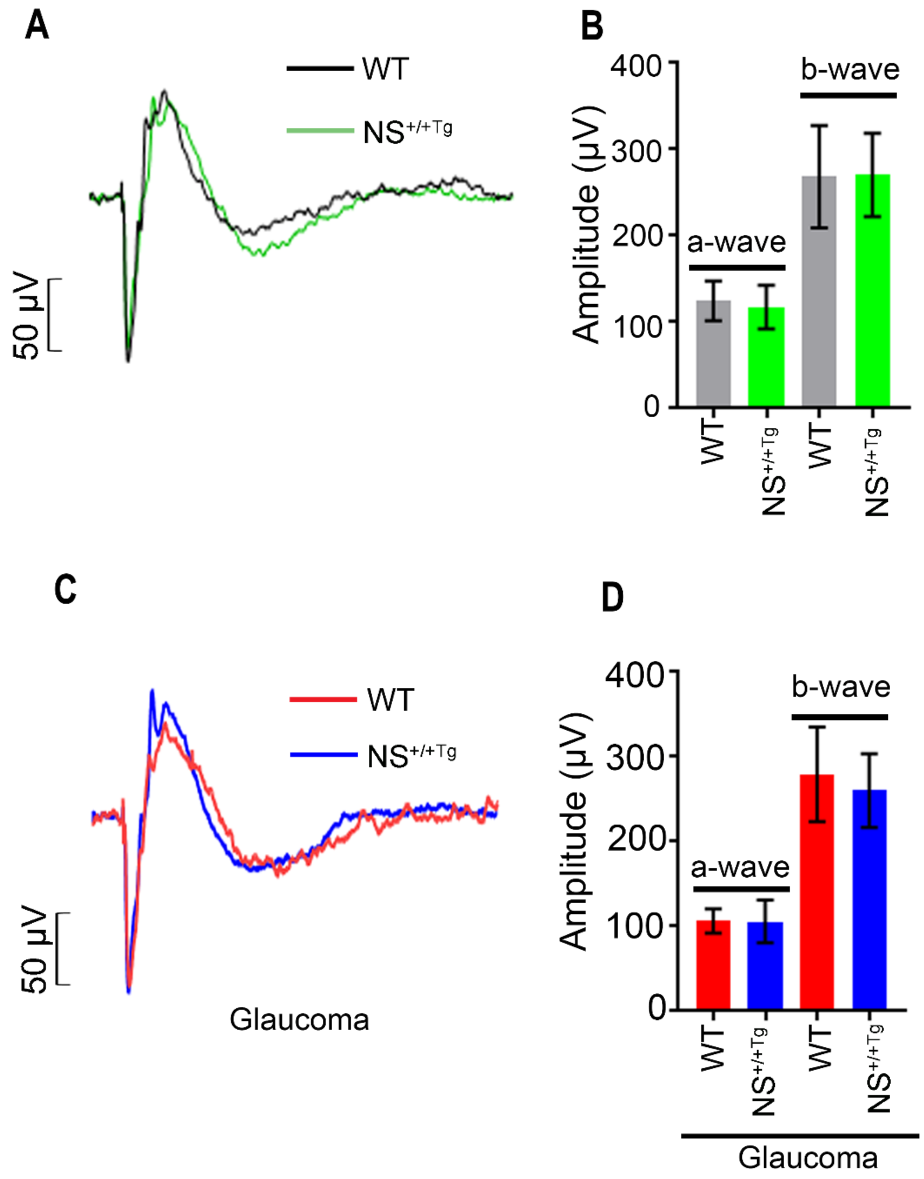

Figure S19 (A) Average ERG traces of WT and $NS^{+/+Tg}$ mice in control IOP conditions. (B) Data analyses of ERG a- and b-wave amplitudes revealed no significant differences between the amplitudes of WT and $NS^{+/+Tg}$ mice. (C) Average ERG traces of WT and $NS^{+/+Tg}$ mice in high IOP condition. (D) Data analyses of ERG a- and b-wave amplitudes showed no significant differences between the amplitudes in glaucoma conditions in WT and $NS^{+/+Tg}$ mice.

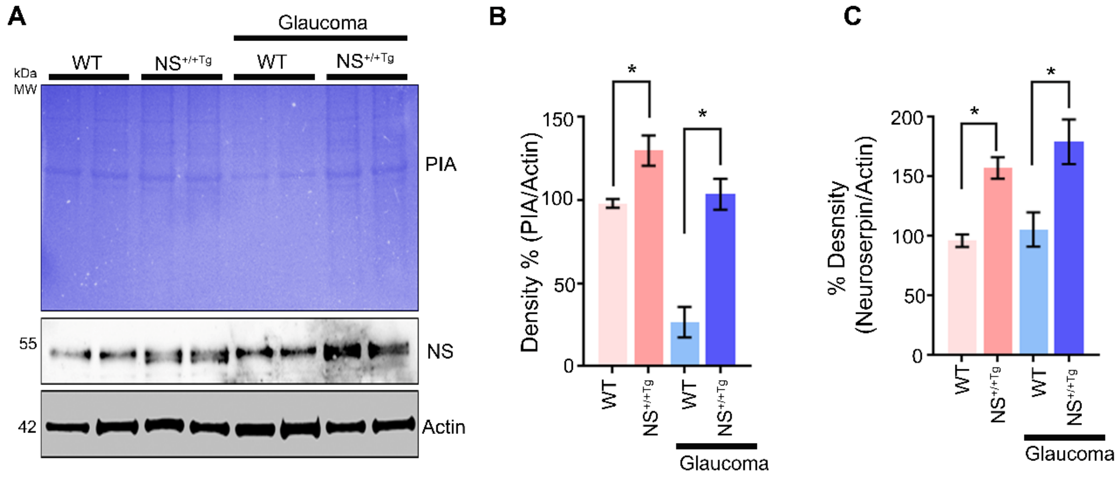

Figure S20 Enhanced plasmin inhibitory activity (PIA) in NS$^{+/+Tg}$ mice in experimental glaucoma (A) retinal lysates from WT and NS$^{+/+Tg}$ mice in control and experimental glaucoma were subjected to gelatin gel zymography to evaluate PIA and neuroserpin immunoreactivity (n = 3 each). Actin was used as a loading control (B) Relative band intensities were quantified, and data analysis indicated significantly decreased plasmin inhibitory activity in WT animal retina subjected to high IOP compared to WT control ($p<0.009$, n=3 animals in each group). NS$^{+/+Tg}$ mice demonstrate significant higher PIA in both control and high IOP condition as compared to their respective WT controls ($p<0.04$ and $p<0.02$, n=3 animals/group) (C) Higher neuroserpin levels were observed in NS$^{+/+Tg}$ mice retinas compared to WT controls ($p<0.01$, $p<0.05$, n=3 animals/group).

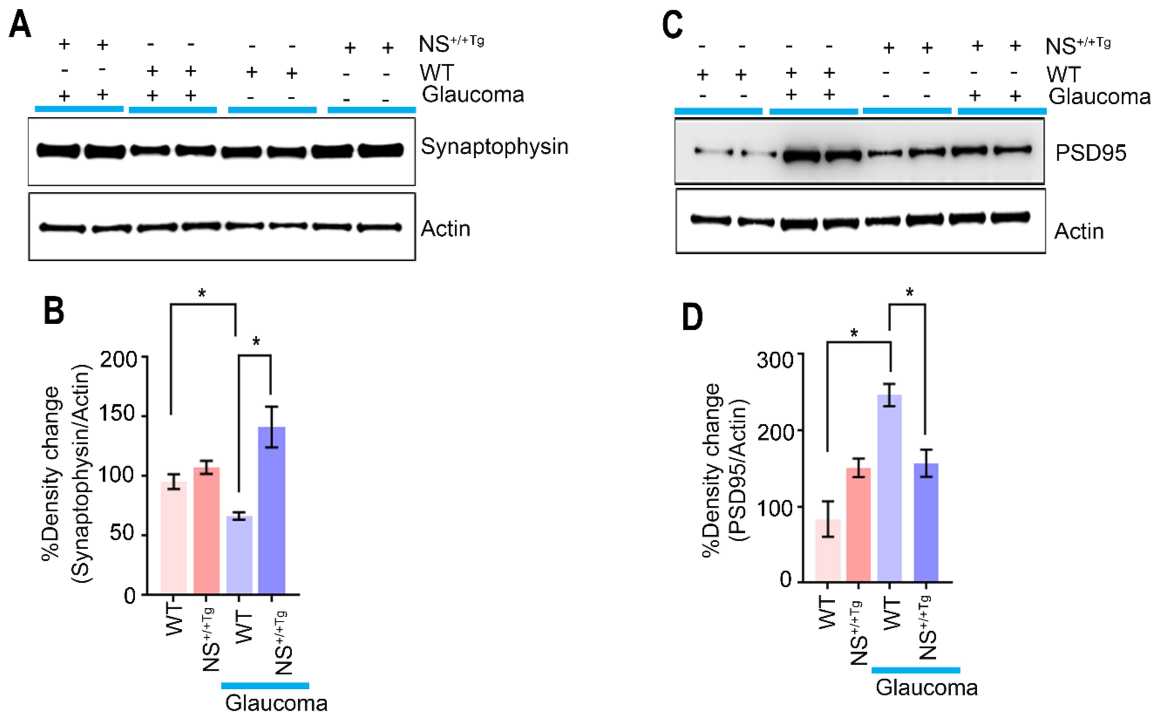

Figure S21 (A) Expression of synaptophysin in retina lysates in WT and NS$^{+/+Tg}$ in normal and glaucoma conditions. (B) Quantification revealed significantly higher synaptophysin levels in NS$^{+/+Tg}$ mice in glaucoma compared to WT control (n=3 animals/group; $p<0.04$ and $p<0.02$). (C) Expression of PSD95 in the retina lysates in WT and NS$^{+/+Tg}$ in normal and glaucoma conditions. (D) Quantification revealed a significant increase in PSD95 expression level in WT in high IOP, but the expression level of PSD95 remained relatively unaltered in NS$^{+/+Tg}$ mice in both healthy and glaucoma conditions (n=3 animals/group; $p<0.02$)

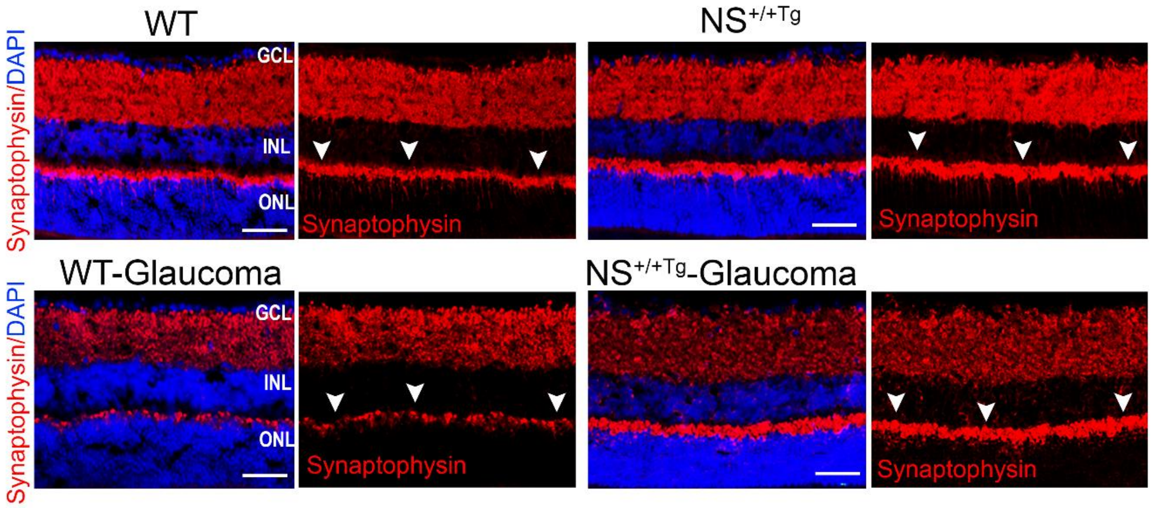

Figure S22 Synaptophysin immunolabeling in WT and $NS^{+/+Tg}$ retina in control and experimental glaucoma. Intense labelling (red) in the inner plexiform layer seen in $NS^{+/+Tg}$ retina in both normal and glaucoma conditions (white arrow). Experimental glaucoma led to reduced synaptophysin labelling in WT mice. DAPI (blue). GCL, ganglion cell layer; INL, inner nuclear layer; PNL, outer nuclear layer. Scale bars=50 μm

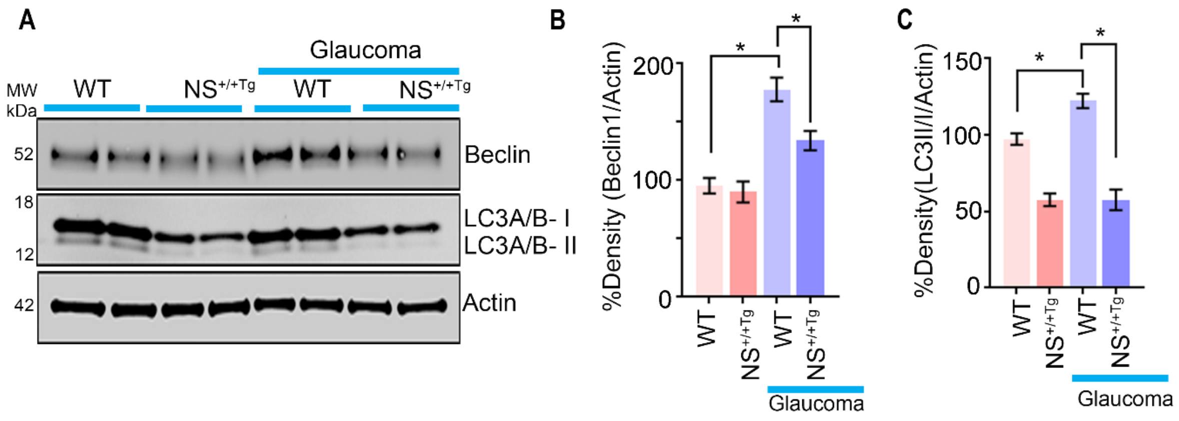

Figure S23 Alterations in autophagy markers in $NS^{+/+\ Tg}$ mice in the control and high IOP retinas (A) Western blot analysis of Beclin-1 and LC3B-II/LC3B-I in WT and $NS^{+/+Tg}$ mice in control and glaucoma conditions. β-actin immunoreactivity was used as a control. (B) Beclin-1 showed a significant increase in WT retinas in experimental glaucoma, while in $NS^{+/+Tg}$ relatively lesser degree of Beclin-1 expression increase was observed in high IOP conditions ($p<0.05$; n=3 animals/group) (C) LC3B-II/LC3B-I revealed a significantly decreased expression in $NS^{+/+Tg}$ compared to WT mice in control and glaucoma conditions ($p<0.03$; n=3 animals/ group). Induction of experimental glaucoma resulted in a significant increase in the expression of LC3B-II/LC3B-I in the WT but not in $NS^{+/+Tg}$ mice under experimental glaucoma conditions ($p<0.03$; n=3 animals/group).

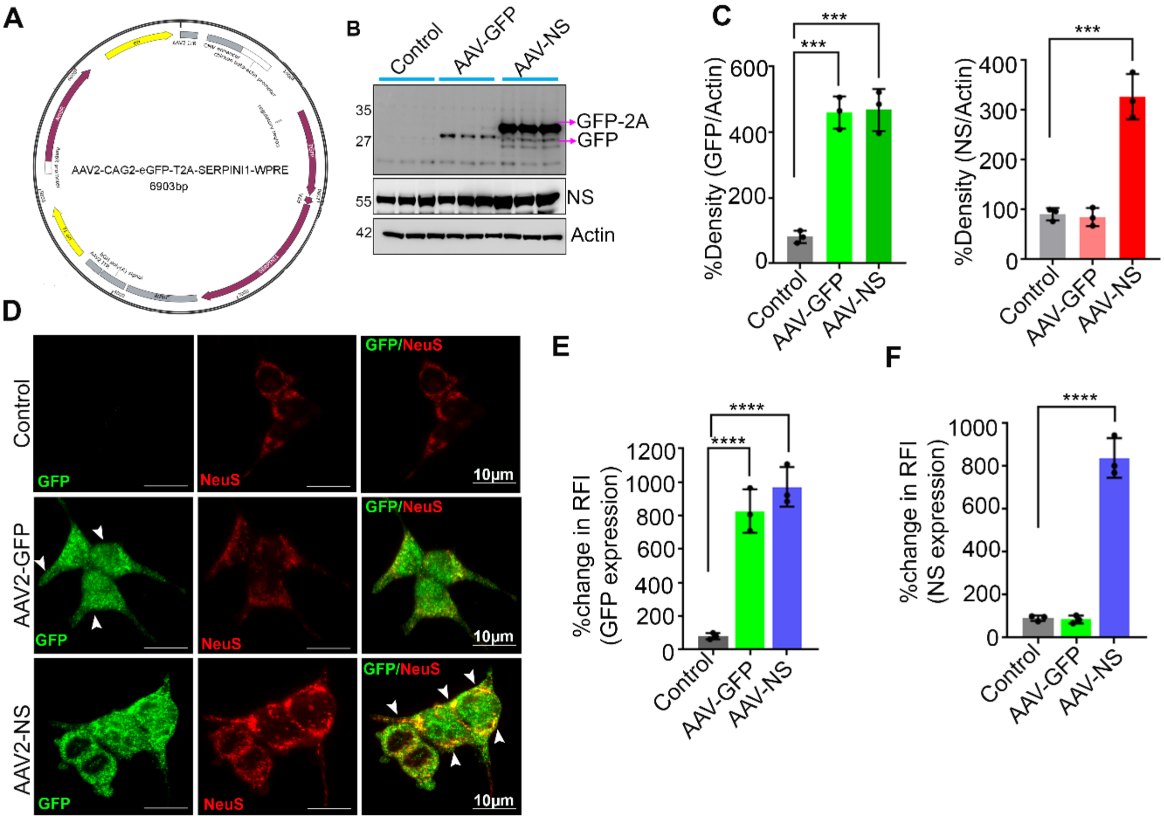

Figure S24 AAV mediated expression of GFP and NS in SH-SY5Y neuronal cells (A) Map of AAV2 plasmid vector expressing human NS with GFP (hNS). hNS sequence was cloned in AAV2 viral vector plasmid fused to the ampicillin-resistance gene. For protein co-expression, a T2A self-cleaving peptide sequence was only used in the AAV-NS construct. (B) WB revealed expression of GFP in AAV-treated SH-SY5Y cells. Neuroserpin protein expression was upregulated in AAV2-hNS treated cells. β-actin was used as a loading control (C) Densitometric Quantification of GFP and NS showing fold changes relative to control SH-SY5Y cells. Data are shown as mean ± SEM (n=3, $p<0.0001$, ANOVA) (D) IF analysis showing control and AAV-transduced SH-SY5Y cells with GFP (green) and NS (red). Cells were transduced either with AAV overexpressing GFP alone or NS (indicated by arrows). Scale 10 µm. Relative fluorescence intensity changes in (E) GFP and (F) neuroserpin expression. ($p<0.0002$, n=3).

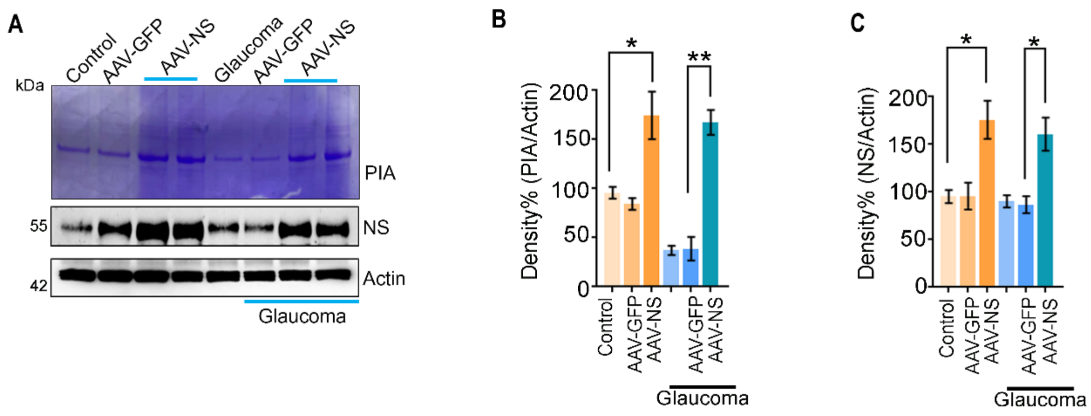

Figure S25 Change in plasmin inhibitory activity upon neuroserpin modulation in control and glaucoma. (A) retina lysates from WT mice treated with AAV-NS and GFP were subjected to gelatin zymography to evaluate the neuroserpin plasmin inhibitory activity (PIA). Actin was used as a loading control (B) Relative band intensities were quantified, and data plotted ($p<0.04$). PIA was significantly reduced in animals subjected to high IOP and AAV-GFP in high IOP conditions ($p<0.009$); however, neuroserpin overexpression in elevated IOP increased PIA compared to glaucoma control ($p<0.001$). (C) Densitometric Quantification shows increased neuroserpin expression in AAV-NS treated mice compared to controls ($p<0.01$). ($n=3$ animals in each group).

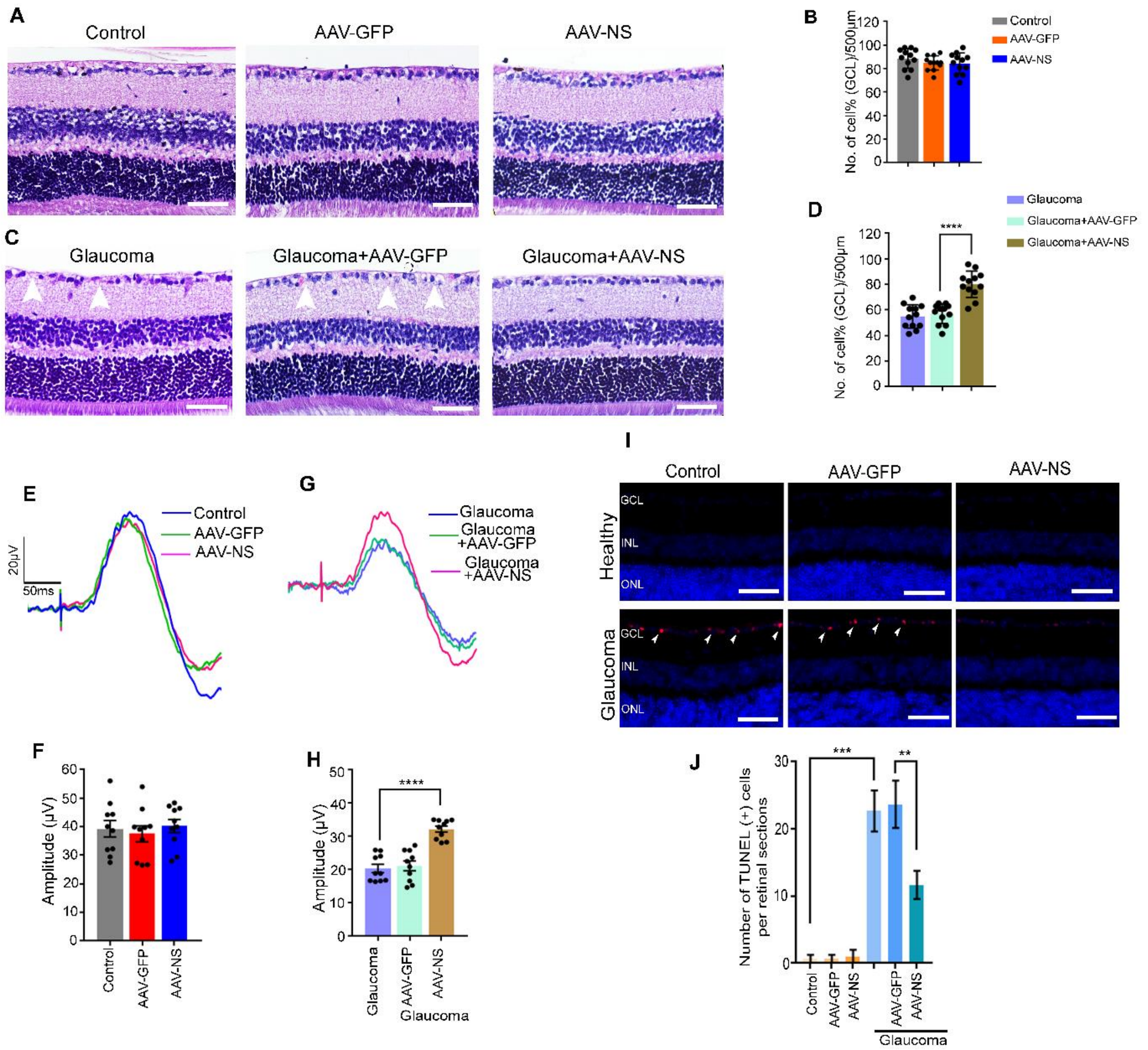

Figure S26 AAV mediated overexpression of neuroserpin protects inner retinal function and structure in chronic glaucoma (A) Histological analysis of paraffin-embedded retinal sections from control, AAV-GFP and AAV-NS treated retinas. Scale bar = 50µm (B) No change in GCL density was observed upon viral vector treatment in control IOP mice (n=4 animals, 3 sections/ animal) (C) Representaive images of retinal section from glaucoma, glaucoma+AAV-GFP and glaucoma+AAV-NS treated retinas stained with H and E. Arrows indicating GCL in the figures Scale bar = 50µm (D) There was a significant decrease in GCL number in WT mice subjected to chronic elevation of IOP and AAV-GFP treated retinas ($p < 0.0001$; $n = 4$ animals, 3 sections/ animal) when compared with AAV-NS treated mice subjected to high IOP (E) pSTR responses in control (blue), AAV-GFP treatment (green), and AAV-NS treatment (pink) in normal IOP condition (F) No change in the pSTR amplitude was observed upon AAV-GFP and AAV-NS treatment compared to control (n=10 animals in each group) (G) pSTR responses glaucoma (blue), glaucoma+AAV-GFP treatment (green), and glaucoma+ AAV-NS treatment in (pink) in high IOP condition (H) Significant lower pSTR amplitude was observed in mice exposed to high IOP and GFP expressing eyes under high IOP condition. Overexpression of neuroserpin significantly protected the RGCs function in glaucomatous eyes ($p<0.0004$; n=10 animals in each group). (I) Increased TUNEL-positive staining (red, white arrows in GCL) was observed

in WT mice retinal sections exposed to high IOP and high IOP+AAV-GFP compared to non-treated, AAV-GFP and AAV-NS treated alone. DAPI-stained cell nuclei (blue). Scale bar, 50 μm. (J) Quantification of TUNEL-positive cells in WT mice retinas exposed to microbead and microbead+AAV-GFP (n=3 animals in each group, $p<0.0001$). AAV-NS overexpression imparted significant protection against apoptosis in experimental glaucoma group (n=4 animals in each group, $p<0.0003$). Graphs show means ± SEM and p values obtained using Student's t test.

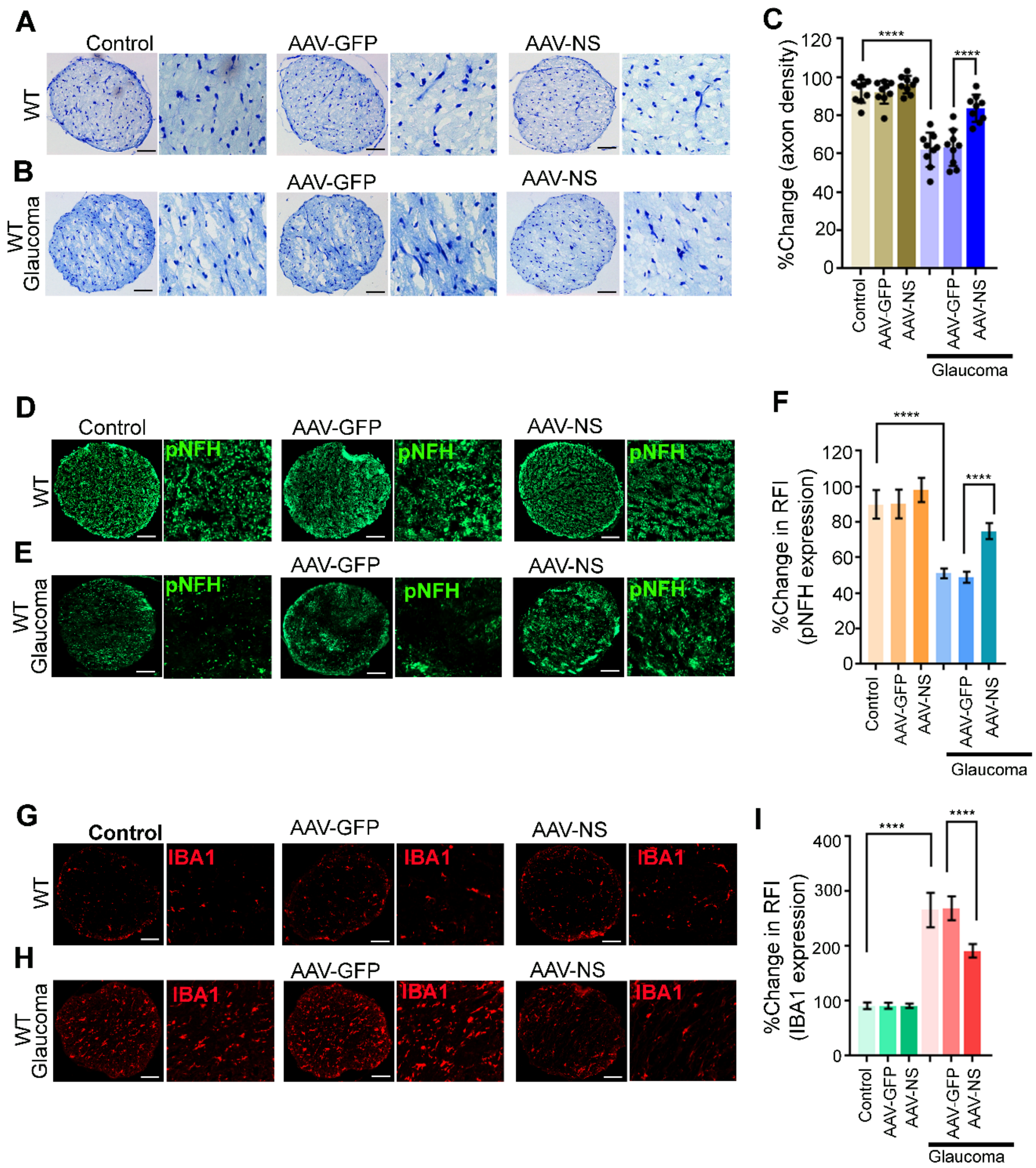

Figure S27 AAV mediated overexpression of neuroserpin in RGCs protects optic nerve against high IOP-induced damage. (A) Cross-sections of WT optic nerve from mice subjected to AAV-GFP and AAV-NS treatment and stained with toluidine blue. (B) Optic nerve sections of glaucoma mice were subjected to AAV treatment and stained with toluidine blue. (C) Overexpression of neuroserpin significantly protected axon density in experimental glaucoma (n= 4 in each group; p<0.003) (D) left-photomicrographs showing pNFH immunostaining in transverse sections of the proximal portion of the optic nerve from eyes injected with AAV2-GFP or AAV2-NS. Enlarged images are shown on the right-side panels. (E) pNFH immunostaining in transverse sections of the proximal portion of the optic nerve from eyes injected with AAV2-GFP or AAV2-NS in high IOP condition. Enlarged images are shown on the right-side panels. (F) A significant decline in pNFH immunoreactivity was noted in experimental glaucoma and glaucoma+AAV-GFP compared to controls and normal IOP

conditions (n= 4 in each group; $p<0.008$). Overexpression of neuroserpin significantly increased the pNFH immunoreactivity in experimental glaucoma (n= 4 in each group; $p<0.003$) (G) photomicrographs showing IBA1 immunostaining patterns in optic nerves from eyes injected with AAV2-GFP or AAV2-NS with enlarged images shown in right panels. (H) photomicrographs showing IBA1 immunostaining patterns in cross-sections of optic nerves from eyes injected with AAV2-GFP or AAV2-NS in glaucoma with enlarged images are shown in the right panels. (I) Significant increase in IBA1 immunoreactivity was observed in experimental glaucoma and glaucoma+AAV-GFP compared to control in normal IOP conditions (n= 4 in each group; $p<0.0001$). Overexpression of neuroserpin significantly reduced the IBA1 immunoreactivity in experimental glaucoma (n= 4; $p<0.0001$). Scale bar = 50μm.

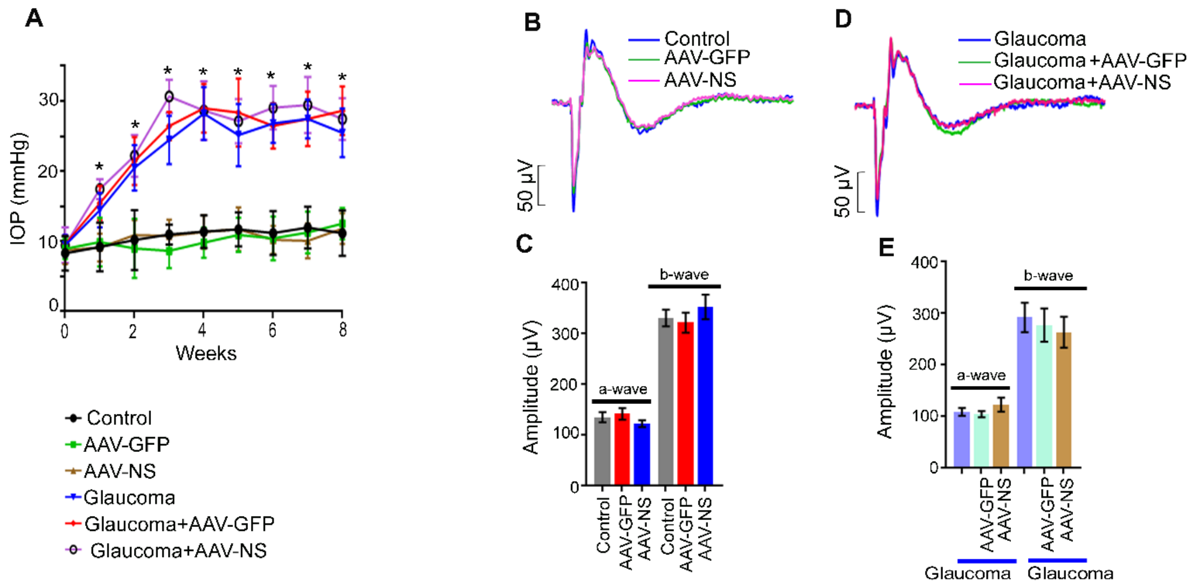

Figure S28 (A) Graph showing IOP changes in WT animal eyes for eight weeks following microbead injections with or without AAV-GFP or AAV-NS treatment (B) Average ERG traces of control, AAV-GFP and AAV-NS treated eyes in the control condition. (C) Data analysis of ERG waves revealed no significant differences in a-wave and b-wave amplitudes upon viral vector treatment compared to control in normal IOP conditions (D) Average ERG traces of glaucoma, glaucoma+AAV-GFP and glaucoma+AAV-NS treated eyes in high IOP condition. (E) Data analysis of ERG waves showed no significant differences in a-wave and b-wave amplitudes upon viral vector treatment in glaucoma.

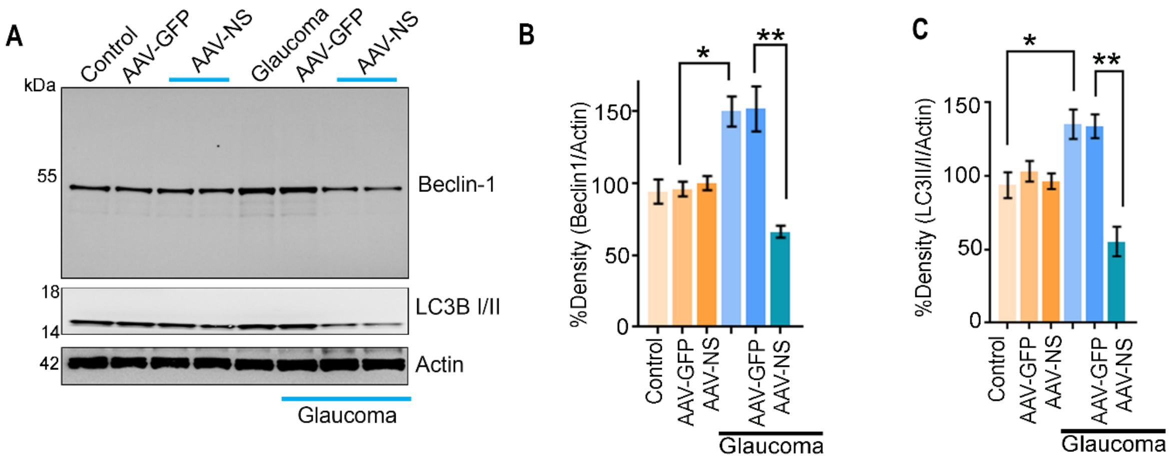

Figure S29 Expression of autophagy markers upon neuroserpin overexpression in the control and high IOP retinas (A) WB of Beclin-1 and LC3B-II/LC3B-I in WT mice retina lysates in control and glaucoma condition. β-actin immunoreactivity was measured as an endogenous control. (B) Densitometric Quantification revealed a significant increase of Beclin1 expression in both experimental glaucoma and glaucoma+AAV-GFP treated mice eyes; however, overexpression of neuroserpin significantly reduced Beclin-1 expression in glaucomatous retina ($p<0.007$) (C) Induction of experimental glaucoma significantly increased the expression of LC3B-II/LC3B-I ratio in microbead and microbead+AAV-GFP mice ($p<0.03$), however, LC3B-II/LC3B-I ratio remain significantly lower in mice overexpressing neuroserpin in glaucoma condition ($p<0.05$). n=3 animals in each group.

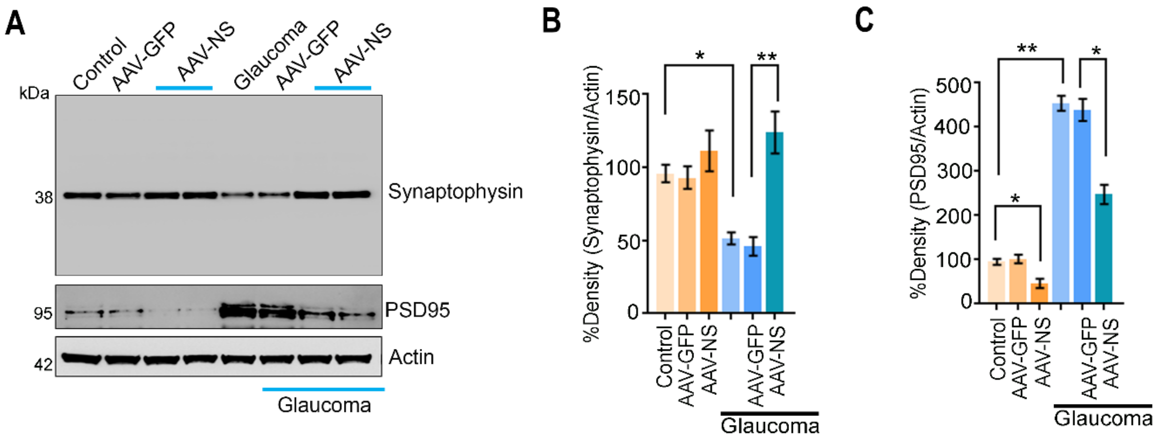

Figure S30 (A) Expression of synaptophysin and PSD95 protein of retina lysates from control, AAV-GFP, and AAV-NS in control and glaucoma. Actin was used as a loading control. (B) Immunoreactivity of synaptophysin showed a significant decrease in retinas subjected to experimental glaucoma. However, the AAV-NS treatment group showed an enhanced synaptophysin immunoreactivity ($p<0.006$) (C) Immunoreactivity of PSD95 showed a significant increase in retinas subjected to experimental glaucoma. However, the AAV-NS treatment group showed a reduced PSD95 expression ($p<0.004$). n=3 animals in each group.

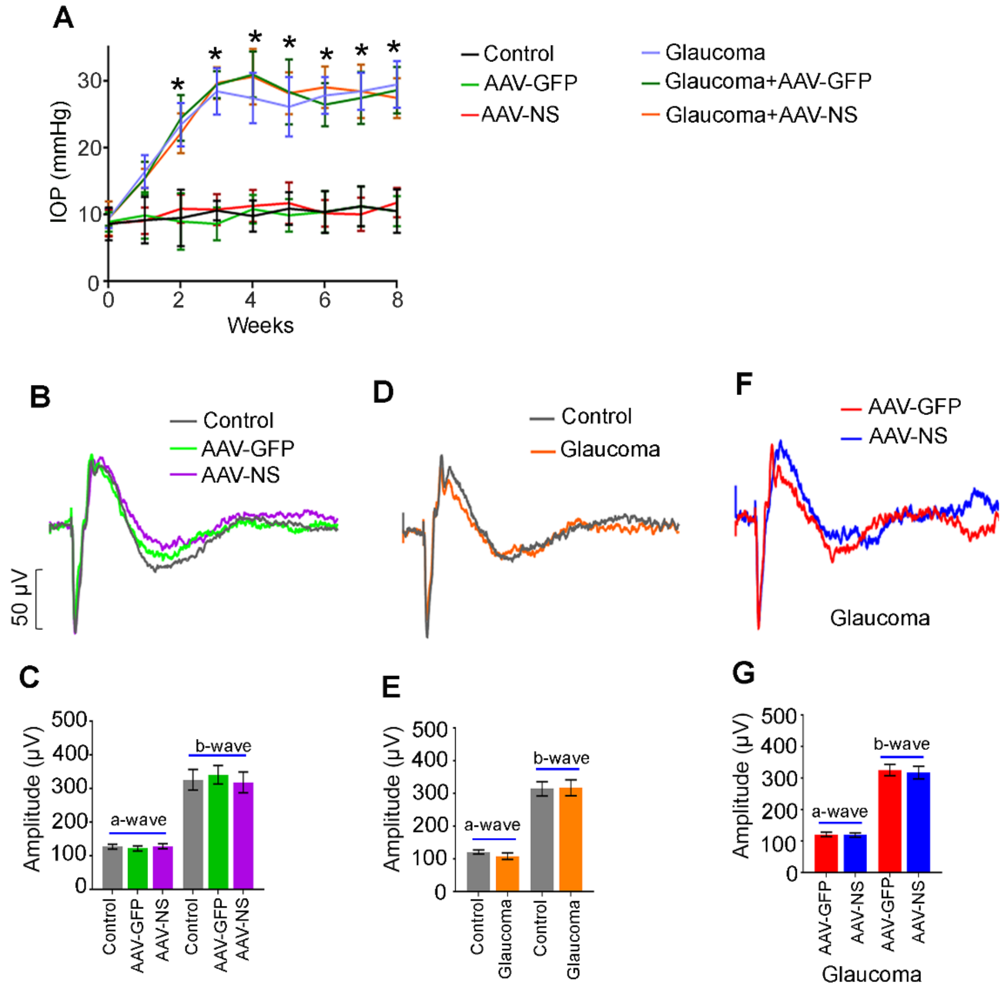

Figure S31 (A) IOP changes in Non injected $NS^{-/-}$ mice (control), AAV-GFP and AAV-NS injected mice during the 8week experiment. Weekly injections of microbeads alone, microbeads+AAV-GFP and microbeads+anti-NS, induced an elevation of IOP (n=10 / group). (B) Average ERG trace of $NS^{-/-}$ control (grey), AAV-GFP (green) and AAV-NS (magenta) treated eyes (C) Data analyses and Quantification of ERG a- and b-wave amplitudes revealed no significant differences between the groups (D) Average ERG traces of $NS^{-/-}$ control (grey) and glaucoma (orange) (E) Quantification of ERG a- and b-wave amplitudes revealed no significant differences between the two groups (F) Average ERG traces of $NS^{-/-}$ glaucoma+AAV-GFP (red) and $NS^{-/-}$ glaucoma+AAV-NS (blue) (G) Quantification of ERG a- and b-wave amplitudes revealed no significant differences between the AAV-GFP and AAV-NS treated groups in experimental glaucoma condition. (n=10 animals in each group).

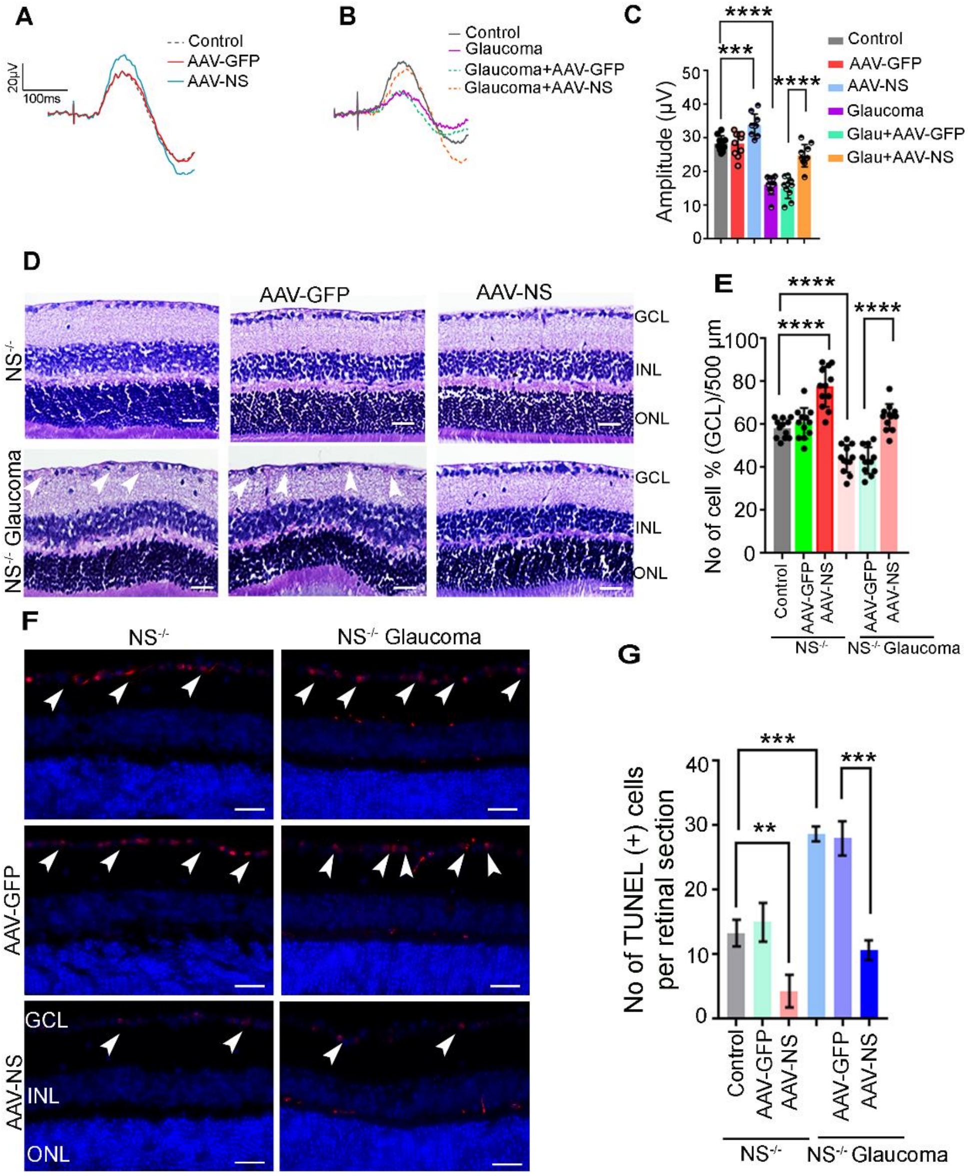

Figure S32 Overexpression of neuroserpin in $NS^{-/-}$ mice retina rescued retinal degeneration phenotype in control and glaucoma condition (A) pSTR response in $NS^{-/-}$ mice retinas in control (dotted black), AAV-GFP (red) and AAV-NS (cyan) treated mice. (B) pSTR traces in control (grey), glaucoma (pink), glaucoma+AAV-GFP (dotted cyan) and glaucoma+AAV-NS (dotted orange) treated $NS^{-/-}$ mice retinas (C) Quantification revealed that AAV-NS treatment in NS ablation mice led to significantly higher pSTR amplitude in normal IOP condition ($p<0.0008$, n=10 animals/ group). Induction of experimental glaucoma significantly lowered pSTR amplitude in $NS^{-/-}$ mice in microbead and microbead+AAV-GFP $NS^{-/-}$ mice retina compared to control ($p<0.0001$, n=10 animals/ group). AAV-NS treatment rescued inner retinal function in in NS ablated mice subjected to high IOP ($p<0.0001$, n=10 animals/ group). (D) H and E analysis of retinal sections from $NS^{-/-}$ retina treated with AAV- GFP or AAV-GFP-NS in normal and high IOP conditions. (Scale bar = 50μm) (E) Quantification revealed $NS^{-/-}$ mice having lower GCL density. Upon neuroserpin upregulation in mice retinas a significant protection of GCL density in $NS^{-/-}$ mice was observed ($p<0.0001$, n=4 animals, 3 sections/

animal). $NS^{-/-}$ mice retinas demonstrated significant decline in GCL density in experimental glaucoma and glaucoma+AAV-GFP compared to control $NS^{-/-}$ mice ($p<0.0001$, n=4 animals, 3 sections/animal). (F) $NS^{-/-}$ and $NS^{-/-}$ overexpressing GFP showed increased TUNEL-positive staining (red) whereas $NS^{-/-}$ mice subjected to AAV-NS treatment had fewer TUNEL positive cells. Induction of experimental glaucoma led to increased TUNEL staining which was reduced in $NS^{-/-}$ mice treated with AAV-NS. DAPI (blue). (Scale bars=50 μm) (G) Quantification of TUNEL-positive cells showed significantly reduced apoptosis in $NS^{-/-}$ mice retinas overexpressing neuroserpin compared to $NS^{-/-}$ control and $NS^{-/-}$+AAV-GFP groups (n=3 animals /group, $p<0.0002$). AAV-NS overexpression in $NS^{-/-}$ mice retina led to reduced TUNEL staining in experimental glaucoma compared to $NS^{-/-}$ mice retinas in glaucoma and glaucoma+AAV-GFP groups (n=3 animals /group, $p<0.0001$). Graphs show means ± SEM and p values obtained using Student's t test.

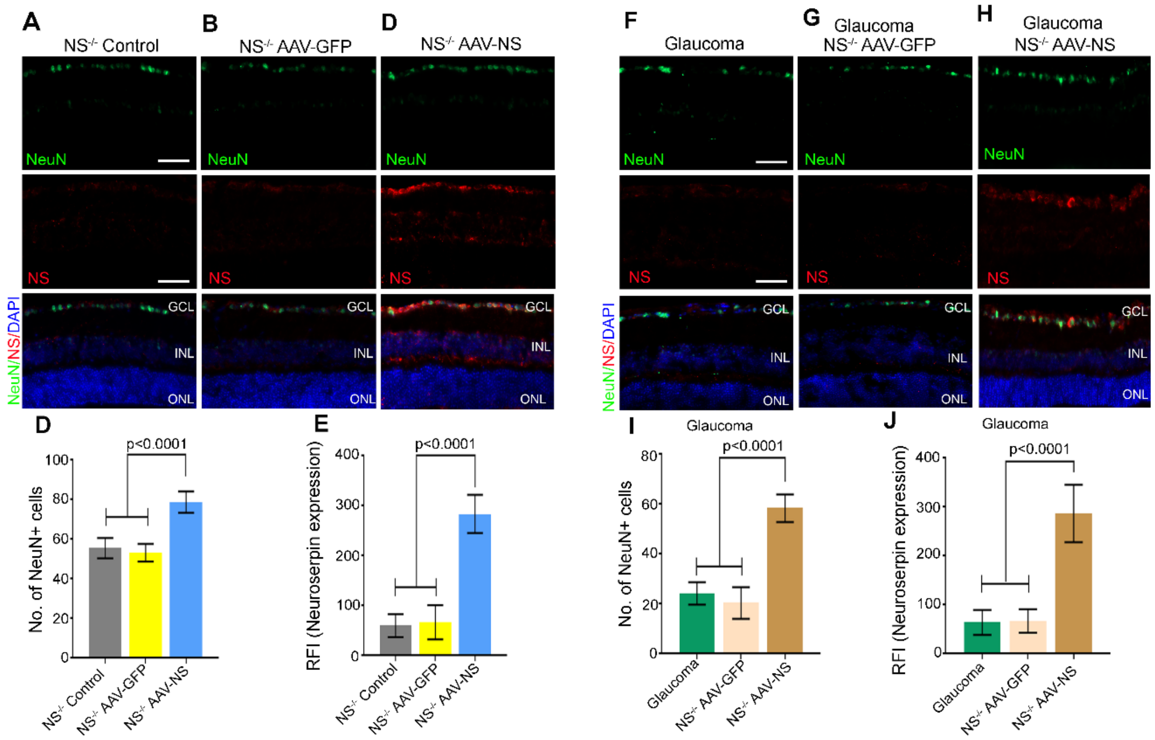

Figure S33 Immunofluorescence images of retinal sections representing (A) NS$^{-/-}$ control, (B) NS$^{-/-}$ AAV-GFP and (C) NS$^{-/-}$ AAV-NS stained with anti-NeuN (green) and anti-neuroserpin (red). (D) Bar graph illustrating the number of NeuN positive (+) cells in the GCL and IPL retina of NS$^{-/-}$ control, NS$^{-/-}$AAV-GFP and NS$^{-/-}$ AAV-NS retinas two-months post AAV treatment (p<0.0001). (E) Bar graph illustrating RFI of neuroserpin expression in the retinas of NS$^{-/-}$ control, NS$^{-/-}$AAV-GFP and NS$^{-/-}$ AAV-NS retinas two-months post AAV treatment (p<0.0001). (F) NS$^{-/-}$ glaucoma, (G) NS$^{-/-}$ glaucoma+AAV-GFP and (H) NS$^{-/-}$ glaucoma+AAV-NS retinal sections stained with anti-NeuN (green) and anti-neuroserpin (red). (I) Bar graph illustrating the number of NeuN positive (+) cells in the GCL and IPL retina of NS$^{-/-}$ glaucoma, NS$^{-/-}$glaucoma+AAV-GFP and NS$^{-/-}$ glaucoma+AAV-NS retinas following two-months of high IOP (p<0.0001). (J) Bar graph illustrating RFI of neuroserpin expression in the retinas of NS$^{-/-}$ glaucoma, NS$^{-/-}$ glaucoma+AAV-GFP and NS$^{-/-}$ glaucoma+AAV-NS retinas following two months of high IOP (p<0.0001). DAPI (blue). GCL, ganglion cell layer; IPL, inner plexiform layer; INL, inner nuclear layer; ONL, outer nuclear layer. n=4 animals / group. Scale bar=50 µm.

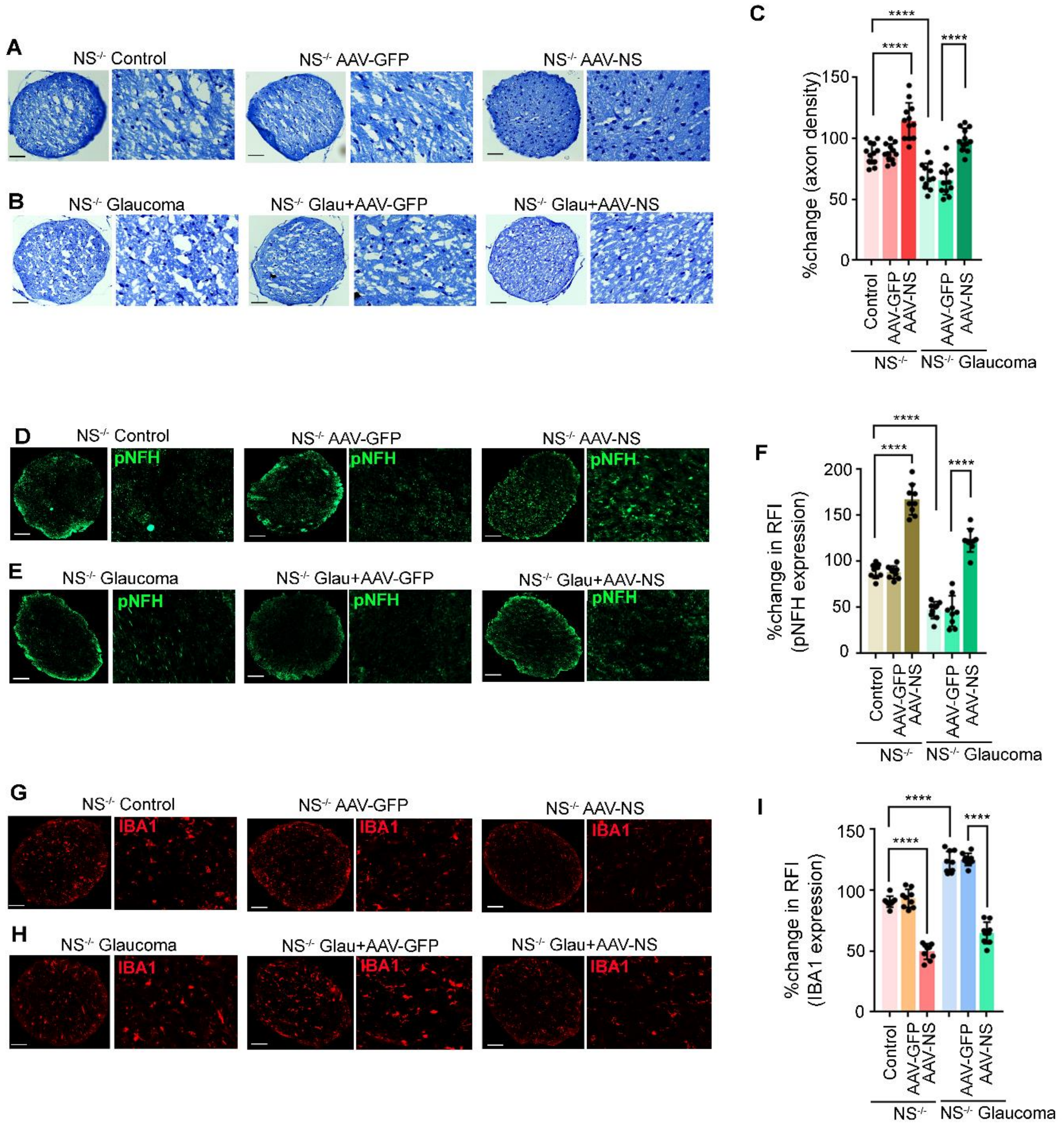

Figure S34 AAV mediated overexpression of neuroserpin in RGCs protects against optic nerve axonal loss in NS$^{-/-}$ mice against glaucomatous damage. Cross sections of optic nerve treated with or without AAV-GFP and AAV-NS and stained with toluidine blue in (A) control and (B) experimental glaucoma condition. (C) Quantification indicating significantly higher axon density in neuroserpin overexpressing NS$^{-/-}$ mice as compared to control condition ($p<0.0004$). Induction of experimental glaucoma in NS$^{-/-}$ mice and NS$^{-/-}$+AAV-GFP mice further reduce the axonal density significantly compared to control ($p<0.002$). Overexpression of neuroserpin significantly protected axonal density in experimental glaucoma ($p<0.003$) (n=4 animals, 3 sections/ animal) (D) Photomicrographs showing pNFH immunostaining in transverse sections of the proximal portion of the optic nerve from eyes injected with AAV2-GFP or AAV2-NS in NS$^{-/-}$ mice. Enlarged images are shown in right side panels. (E) photomicrographs showing pNFH immunostaining in transverse sections of the proximal portion of the optic nerve from eyes injected with AAV2-GFP or AAV2-NS in high IOP condition. Enlarged images are shown

in right side panels. (F) Quantification of pNFH IF intensity in optic nerve sections showing significantly increased expression in AAV-NS expressing control ($p<0.003$) and experimental glaucoma ($p<0.0004$) (n=3 animals, 3 sections/ animal) (G) Photomicrographs showing ionized calcium-binding adaptor molecule 1 (IBA1) immunostaining $NS^{-/-}$ mice optic nerve sections from eyes injected with AAV2-GFP or AAV2-NS in control condition. (H) Photomicrographs showing IBA1 immunostaining in optic nerves from $NS^{-/-}$ mice eyes injected with AAV2-GFP or AAV2-NS in glaucoma condition. Enlarged images are shown in right panels. (I) Quantification of IBA1 fluorescence intensity showing reduced IBA1 expression in AAV-NS treated $NS^{-/-}$ mice in control ($p<0.0001$) and glaucoma conditions ($p<0.0001$). n= 3 animals, 3 sections/ animals. (Scale bar = 50µm). Graphs show means ± SEM and p values obtained using Student's t test.

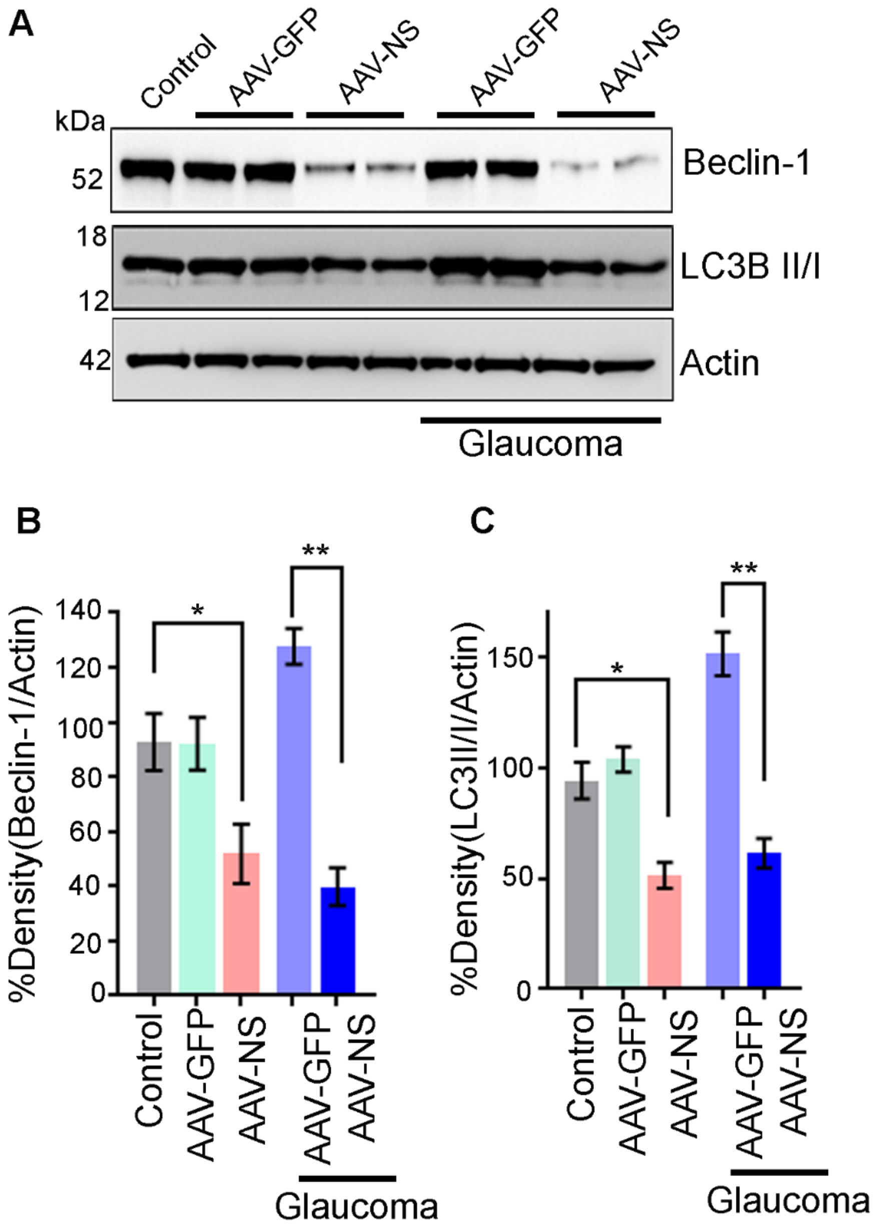

Figure S35 Autophagy changes upon neuroserpin modulation in the $NS^{-/-}$ mice in control and high IOP subjected retinas (A) WB analysis of Beclin-1 and LC3B-II/LC3B-I. β-actin immunoreactivity was measured as an endogenous control. The changes in the AAV-GFP, AAV-NS and microbead, microbeads+AAV-GFP and mirobeads+AAV-NS retinas relative to control were evaluated. (B) Immunoreactivity of Beclin-1 showed a significant decrease in retinas overexpressing neuroserpin in $NS^{-/-}$ mice compared to control ($p<0.05$). Induction of experimental glaucoma induced elevation of Beclin-1 immunoreactivity; however, neuroserpin overexpression in glaucoma further downregulates Beclin-1 expression ($p<0.006$). (C) Densitometric Quantification of LC3B-II/LC3B-I ratio showed a significant decrease in AAV-NS treated $NS^{-/-}$ group in control IOP ($p<0.03$). After high IOP elevation in $NS^{-/-}$ mice, a significant increase in the expression of LC3B-II/LC3B-I ratio was evident: however, overexpression of neuroserpin in glaucoma further downregulated LC3B-II/LC3B-I ratio ($p<0.009$). n=3 animals in each group.

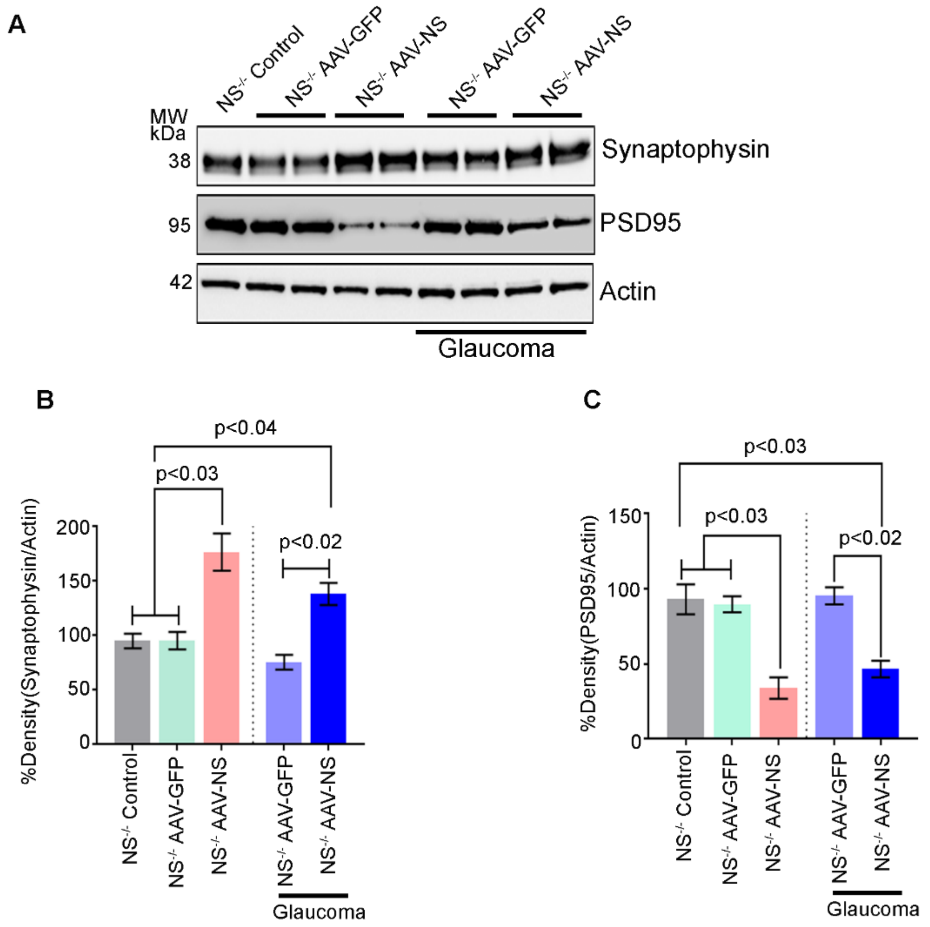

Figure S36 WB is showing changes in pre-and post-synaptic markers following AAV-NS treatment in the NS$^{-/-}$ mice control and high IOP retinas (A) Expression of synaptophysin and PSD95 in ONH lysates from NS$^{-/-}$ mice control, NS$^{-/-}$ AAV-GFP, NS$^{-/-}$ AAV-NS, NS$^{-/-}$ microbead, NS$^{-/-}$ microbead+AAV-GFP and NS$^{-/-}$ microbead+AAV-NS treated mice retina. Actin was used as a loading control. (B) Immunoreactivity of synaptophysin showed a significant increase in retinas overexpressing neuroserpin in both controls ($p<0.03$) and glaucoma conditions ($p<0.02$). (C) Immunoreactivity of PSD95 showed a significant decrease in retinas treated with AAV-NS in control ($p<0.03$); however, overexpression of neuroserpin in glaucoma downregulated the PSD95 expression ($p<0.02$). n=3 animals in each group.

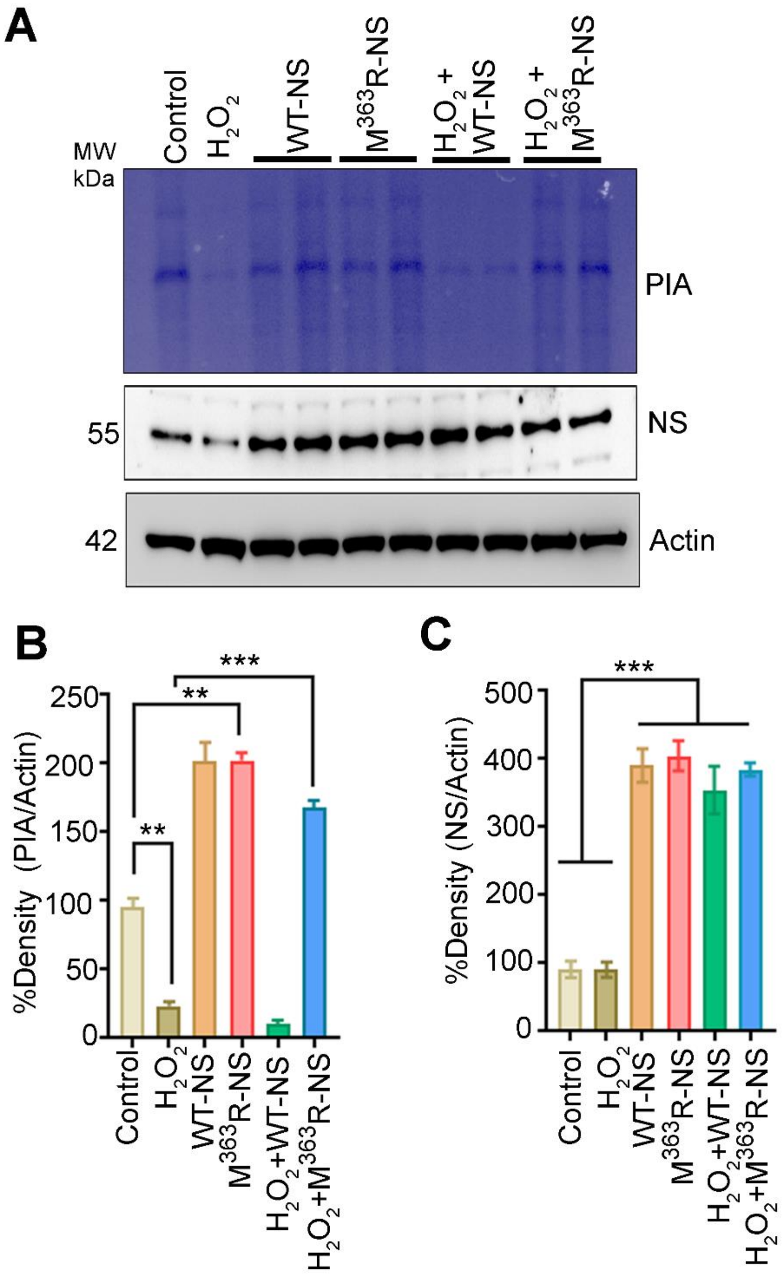

Figure S37 SH-SY5Y cells subjected to WT and M^{363}R-NS plasmid (pSF-CAG-WT/M363R-NS-6xHisTag-2A-eGFP) expression were subjected to H_2O_2 oxidative stress. (A) Plasmin inhibitory gelatin gel zymography and NS expression in WB from control, H_2O_2, WT-NS and M^{363}R -NS treated cells were assessed. Actin was used as a loading control (B) Relative band intensities were quantified, and data analysis indicated significantly lower PIA in H_2O_2 treated conditions ($p<0.005$). WT-NS and M^{363}R -NS expressing SH-SY5Y cells showed significantly higher PIA activity compared to control ($p<0.02$ and $p<0.003$). M^{363}R -NS expressing cells in H_2O_2 oxidative stress condition led to significantly higher PIA reactivity as compared to WT-NS treated cells ($p<0.0008$) (C) Significantly higher expression of neuroserpin was noted in cells treated with WT-NS and M^{363}R -NS plasmid (pSF-CAG-WT/M363R-NS-6xHisTag-2A-eGFP) 24 hrs after plasmid transfection compared to the empty vector-transfected cells ($p<0.002$, n=3 in each group)

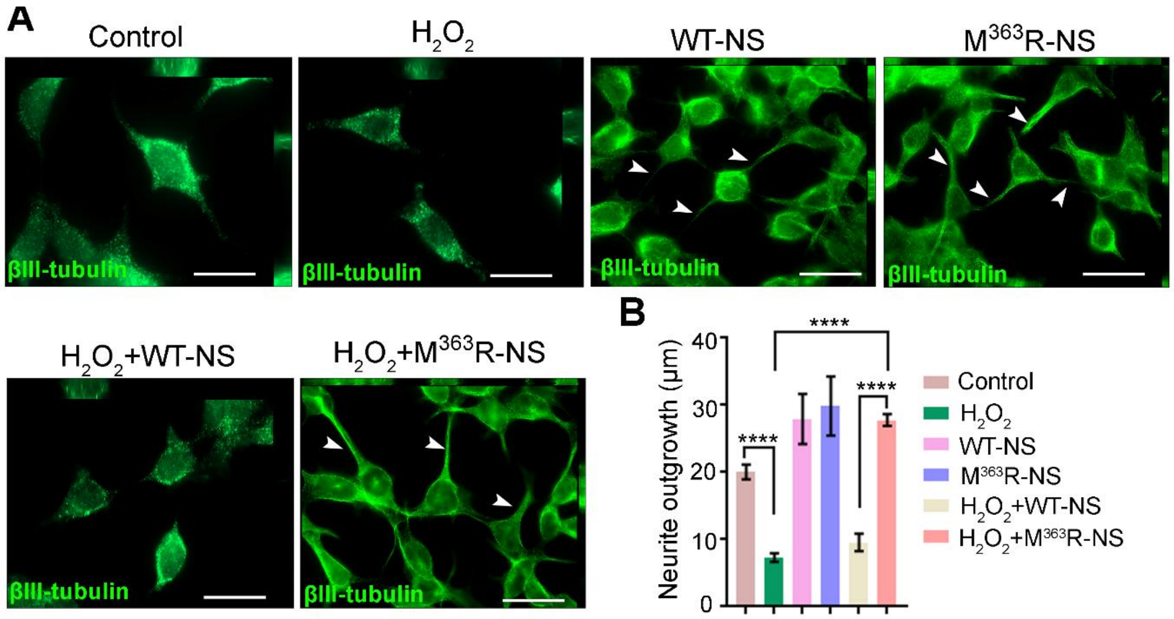

Figure S38 Modified neuroserpin overexpression induced neuritogenesis in SH-SY5Y cells (A) Neuronal cells subjected to WT-NS and M^{363}R-NS plasmid transfection were stained for βIII tubulin using IF analysis and neurite lengths assessed with and without of H_2O_2 treatment (B) A significant reduction in neurite growth ($p<0.0001$) was observed in H_2O_2 stress condition while an increase was observed in M^{363}R-NS ($p<0.0001$) overexpression conditions ($p<0.03$ and $p<0.002$; $n = 6$). M^{363}R-NS has significantly higher neurite outgrowth than WT-NS in oxidative stress ($p<0.0005$, $n=3$ samples). Scale bar=50μm.

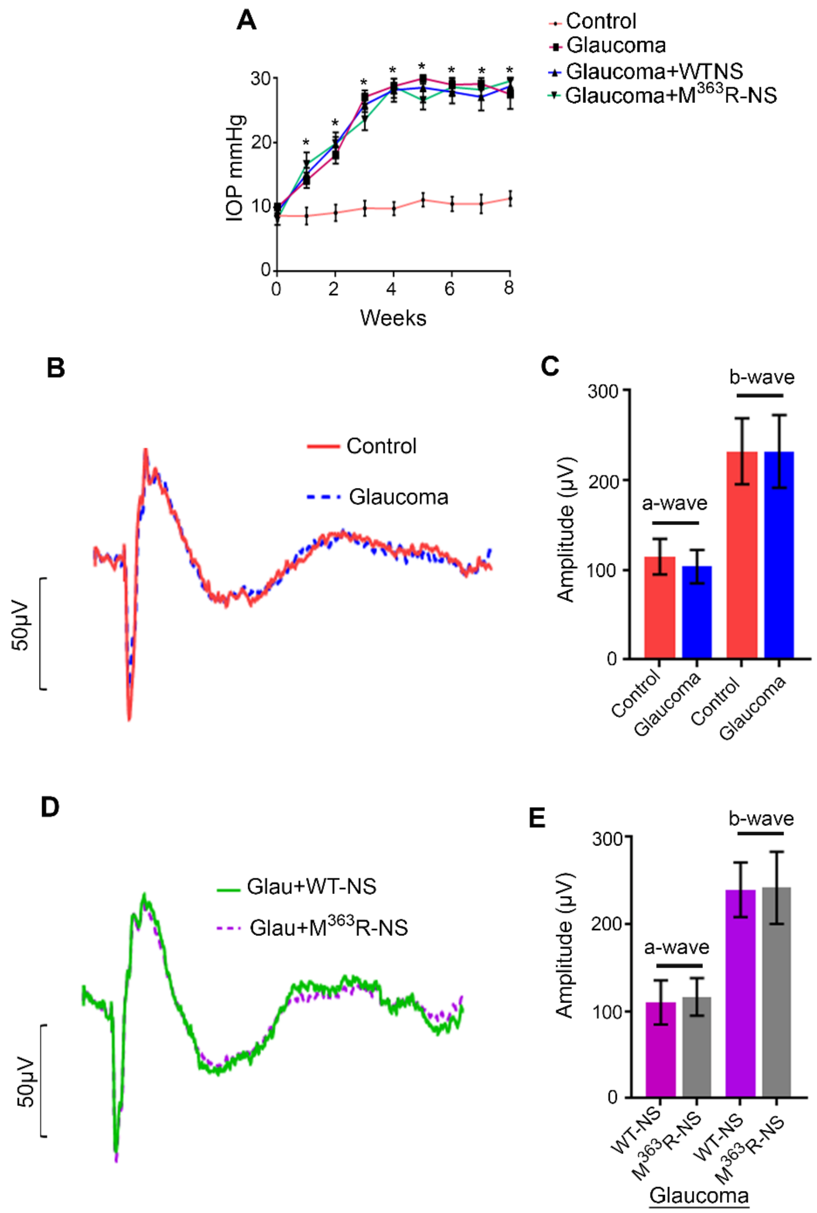

Figure S39 (A) Control mice showed an average value of 9.95±2.1 mmHg during the experimental period of 8 weeks. Weekly injections of microbeads resulted in the elevation of IOP (average value of 26.76±2.5 mm Hg at eight weeks) (B) Average ERG trace of WT control (red) and microbead administered (blue) eyes (C) Data analyses of ERG a- and b-wave amplitudes revealed no significant differences between the control and glaucoma groups (D) Average ERG traces of microbeads+WT-NS (green) and microbeads+M^{363}R-NS (magenta) administered eyes (E) No significant differences were observed between the WT-NS and M^{363}R -NS administered groups in glaucoma conditions. n=10/ group.

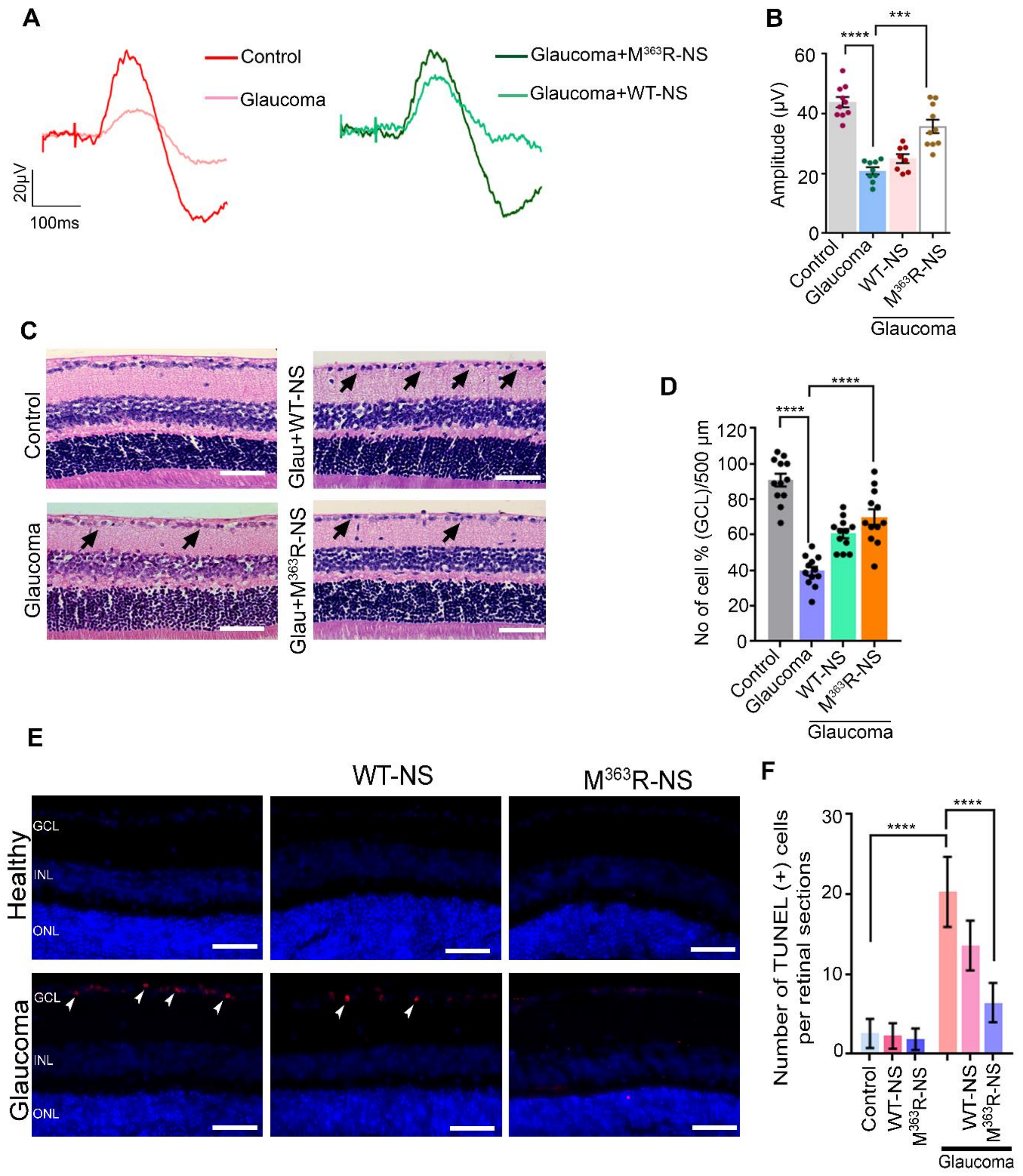

Figure S40 M^{363}R neuroserpin administration protects inner retinal function and structure in chronic glaucoma. (A) pSTR traces from control (red), chronic glaucoma models of WT (pink) and WT-NS (dark green) and M^{363}R-NS (light green) protein treated animals (weekly intravitreal administration for 8 weeks, 10 µmol/L, vol 2µl) in chronic glaucoma (B) Intravitreal administration of WT-NS and Mut-NS protein significantly protected the pSTR amplitude in experimental glaucoma. Higher protection of pSTR amplitude was noted in M^{363}R -NS protein administration in high IOP compared to WT-NS treatment ($p<0.04$, n=10 animals/ group) (C) H and E staining of retinal sections from control, glaucoma, glaucoma+WT-NS and glaucoma+ M^{363}R -NS treated retinas. (D) There was a significant decrease in GCL density in WT mice under chronic elevation of IOP ($p < 0.0001$; n=4 animals, 3 sections/ animal) when

compared with control. WT-NS administration in experimental glaucoma significantly protected against GCL loss compared to glaucoma ($p<0.001$). Further protection was evident in animals administered Mut-NS in high IOP compared to one overexpressing WT-NS protein ($p<0.008$). (E) Experimental glaucoma group showed increased TUNEL positive cells (red) in the GCL layer. WT-NS administration led to reduced TUNEL staining in glaucoma whereas $M^{363}R$ -NS treatment had much fewer TUNEL positive cells. DAPI (blue). (F) Quantification revealed significantly increased TUNEL-positive cell number in WT mice retinas exposed to high IOP ($p<0.0001$). WT-NS ($p<0.006$) and $M^{363}R$ -NS ($p<0.0001$) administered retina was significantly protected in experimental glaucoma compared to only high IOP exposed mice (n=3 animals / group). Scale bar = 50μm. Graphs show means ± SEM and p values obtained using Student's t test.

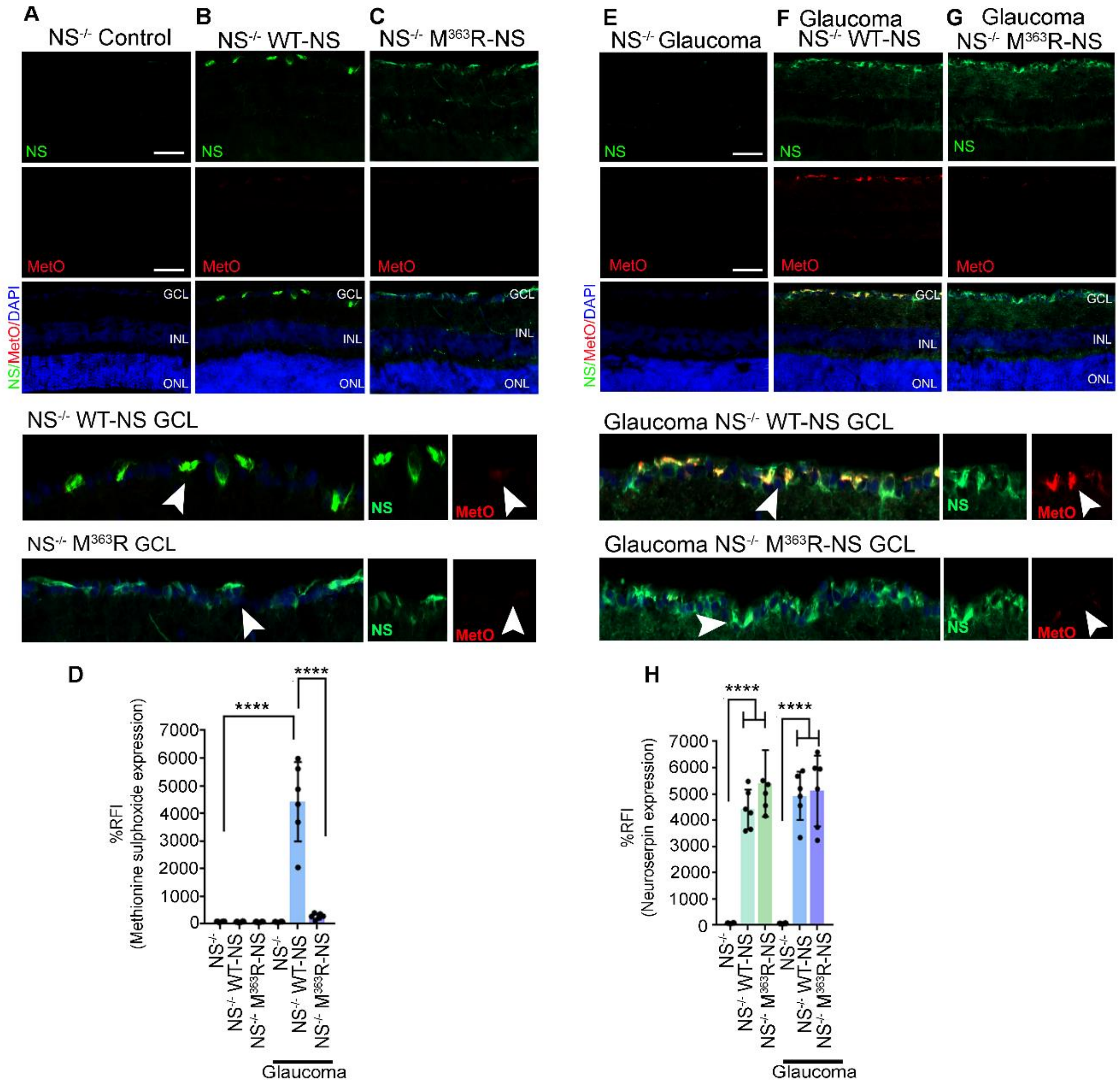

Figure S41 M^{363}R neuroserpin is resistant to oxidation in glaucoma. WT mice were treated with either AAV-WT-NS or AAV-Mut-NS. Retinal sections were stained against neuroserpin (green), methionine sulfoxide (red) and DAPI (blue) (A) control (B) WT-NS and (C) M^{363}R - NS. Magnified GCL with neuroserpin and methionine sulfoxide immunostaining in WT-NS and M^{363}R -NS treated WT mice retina is shown at the bottom. (D) The retinal section was stained with neuroserpin (green), methionine sulfoxide (red) and DAPI (blue) in experimental glaucoma (E) WT mice treated with WT-NS and (F) WT mice treated with M^{363}R -NS and their retinal section were stained with neuroserpin (green), methionine sulfoxide (red) and DAPI (blue) in experimental glaucoma. Magnified GCL with neuroserpin and methionine sulfoxide immunostaining in WT-NS and M^{363}R -NS treated WT mice retina. (G) Quantifying the MetS immunoreactivity in GCL of WT mice treated with either WT-NS or M^{363}R -NS in the control and experimental glaucoma conditions. MetS reactivity in WT-NS treatment retina is significantly higher compared to the M^{363}R -NS treated in experimental glaucoma ($p<0.0001$). (H) WT-NS and M^{363}R -NS administration in control ($p<0.001$ and $p<0.0004$ respectively) and glaucoma ($p<0.0001$) led to significantly higher expression of neuroserpin compared to control retinas. n=4 animals in each group.

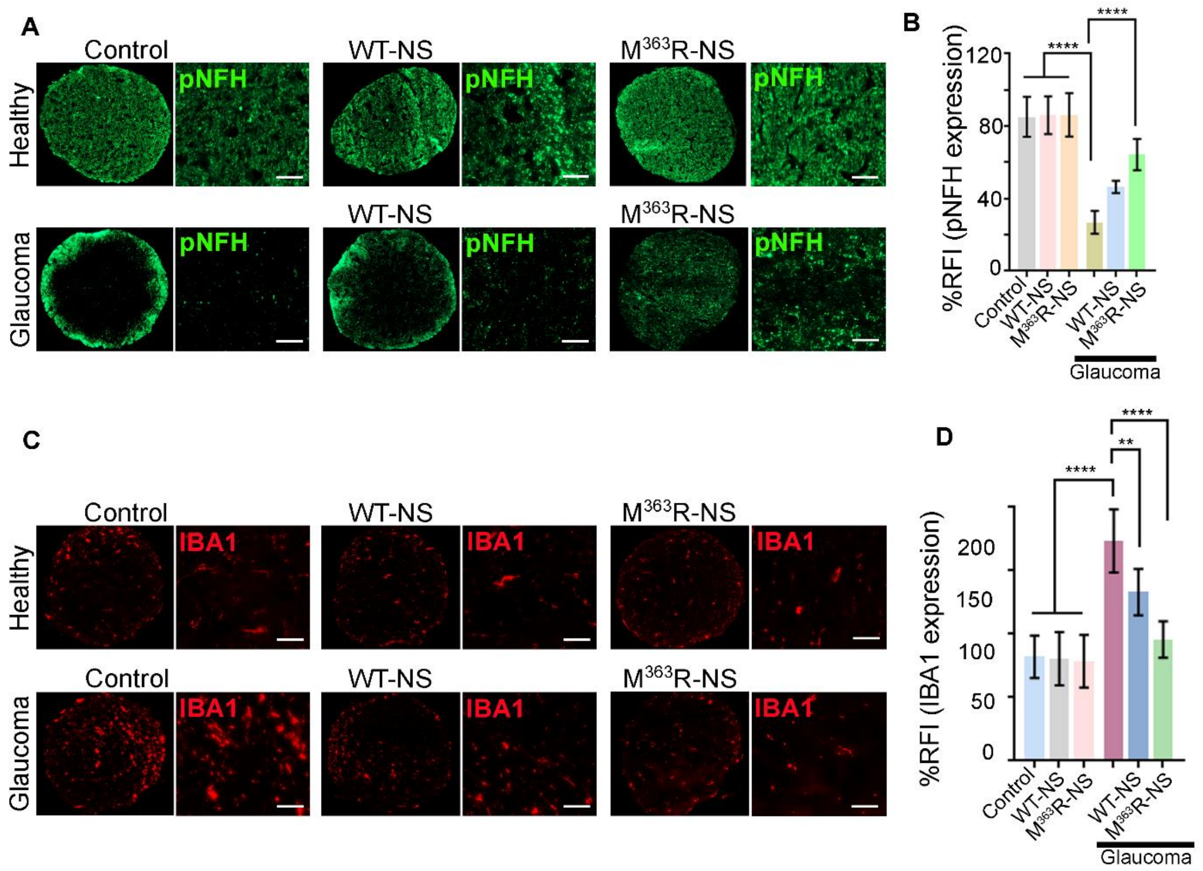

Figure S42 $M^{363}R$ NS administration in WT mice RGCs protects optic nerve against glaucomatous damage. (A) Phosphorylated neurofilament heavy-chain (pNFH) immunoreactivity in control and glaucoma conditions treated with either WT-NS or $M^{363}R$ NS protein. Enlarged images are shown on the right side. (B) Intravitreal administration of WT and $M^{363}R$ NS protein has no significant changes in pNFH immunoreactivity in the control condition. Induction of glaucoma led to a significant reduction in pNFH immunoreactivity, which was reduced in tissues from mice treated with WT-NS and ($p<0.0001$) $M^{363}R$ NS ($p<0.0004$) protein. (C) Photomicrographs showing ionized calcium-binding adaptor molecule 1 (IBA1) immunostaining patterns in WT mice optic nerves cross-sections from eyes injected with or without WT-NS or Mut-NS protein in control and glaucoma conditions. Enlarged images are shown towards the right (D) Control, WT-NS, and $M^{363}R$ NS protein injected WT mice had no significant change in IBA1 immunoreactivity in the control condition. Experimental glaucoma significantly increased IBA1 immunoreactivity compared to control ($p<0.0005$). Administration of WT-NS ($p<0.01$) and $M^{363}R$ NS ($p<0.0001$) significantly lowered IBA1 immunoreactivity in glaucoma; however, Mut-NS protein showed a greater decline in IBA1 immunoreactivity compared to WT-NS protein ($p<0.003$) administered mice. Scale bar = 10µm. n=4 animals in each group.

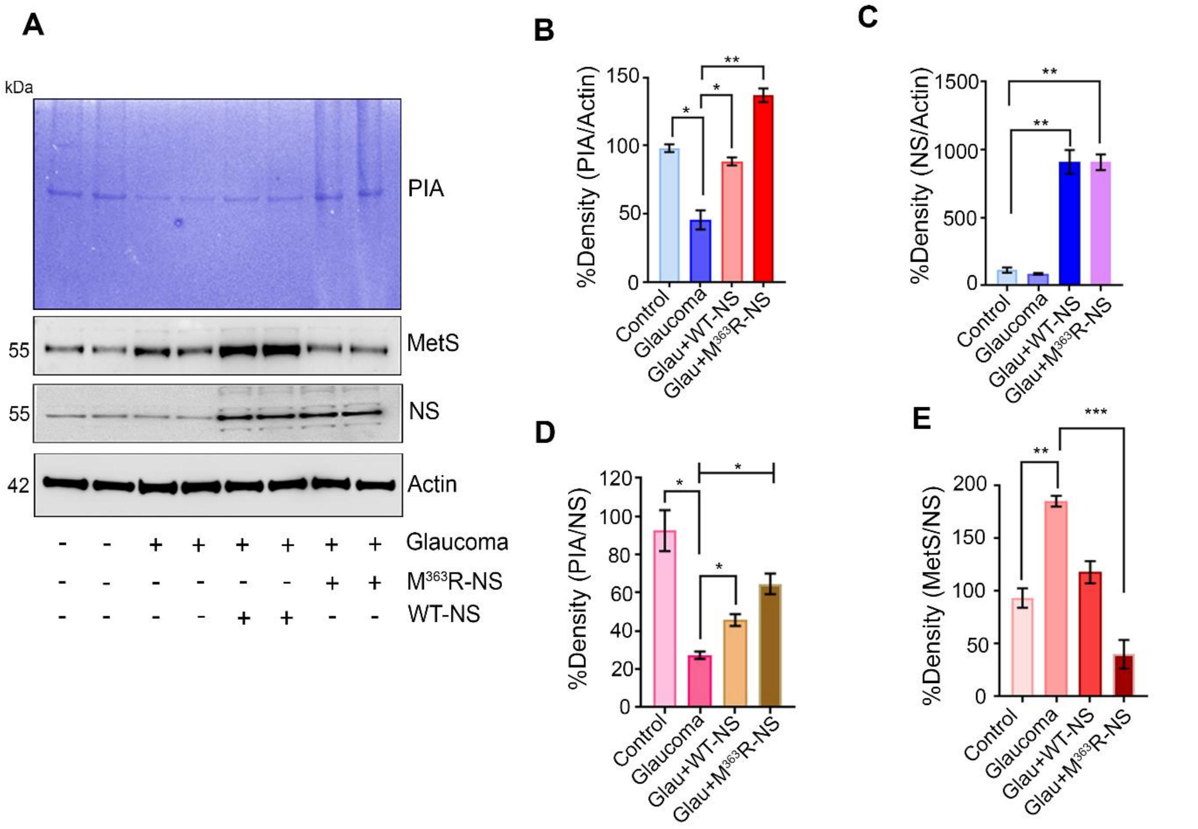

Figure S43 Plasmin inhibitory activity of M^{363}R-NS in retinal tissues subjected to exogenous NS treatment (A) Gelatin gel zymography showing PIA from control, glaucoma, glaucoma+WT-NS and glaucoma+ M^{363}R-NS treatment in high IOP condition. Retina lysates were loaded for WB and probed for neuroserpin and methionine sulfoxide (MetS) immunoreactivity. Actin was used as a loading control (B) Relative band intensities were quantified, and data analysis indicated significantly lowered PIA in glaucoma condition compared to control ($p<0.01$). PIA was significantly higher in retinas administered WT ($p<0.05$) and M^{363}R-NS ($p<0.02$), and a higher PIA was observed in M^{363}R-NS compared to WT-NS ($p<0.01$). (C) Higher levels of WT ($p<0.006$) and M^{363}R- neuroserpin ($p<0.0007$) were detected in the retinas of animals administered exogenous NS. (D) Relative band intensities were quantified, and data analysis indicated significantly higher PIA in retinal samples subjected to M^{363}R-NS treatment than the WT NS treatment ($p<0.05$). (E) Relative band intensities were quantified, and data analysis indicated significantly higher MetS reactivity in experimental glaucoma compared to control ($p<0.007$). Significantly lower MetS reactivity was observed in the M^{363}R-NS treatment subjected retinas compared to WT-NS treated retinas ($p<0.006$). n=3 animals in each group.

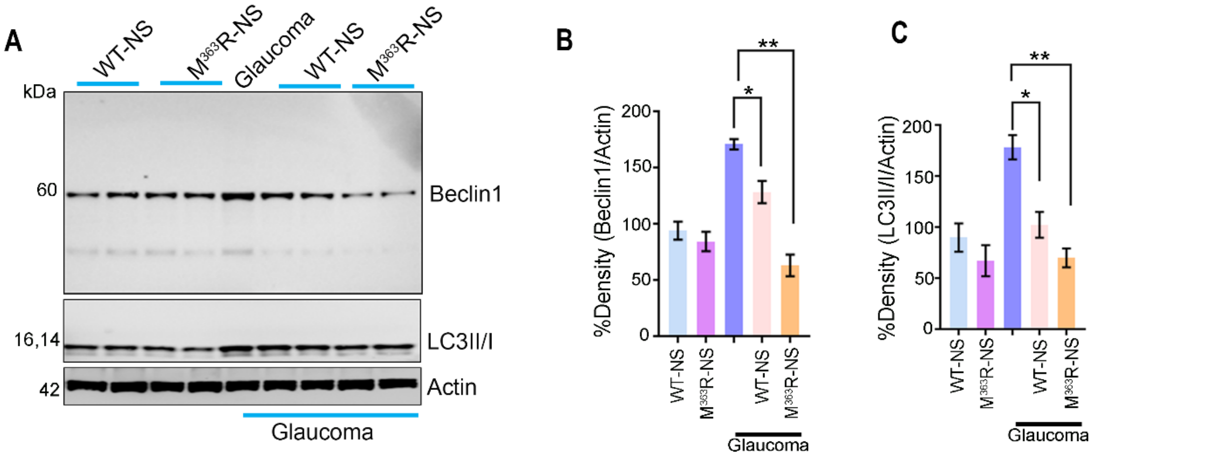

Figure S44 Alterations in autophagy markers upon WT and M^{363}R-NS administration under control and glaucoma condition (A) WB analysis of retinal lysates against Beclin-1 and LC3B-II/LC3B-I immunoreactivity in response to WT-NS and M^{363}R-NS administration in control and microbead administered eyes. β-actin immunoreactivity was measured as an endogenous control. (B) Immunoreactivity of Beclin-1 showed an increase in glaucomatous eyes. Significantly reduced Beclin-1 immunoreactivity was observed in eyes subjected to WT-NS ($p<0.05$) and M^{363}R-NS ($p<0.006$) administration (C) In the high IOP subjected group, the expression of LC3B-II/LC3B-I ratio was higher, however, WT ($p<0.03$) and M^{363}R - NS ($p<0.009$) administered glaucomatous group demonstrated reduced LC3B-II/LC3B-I ratio. n=3 animals in each group.

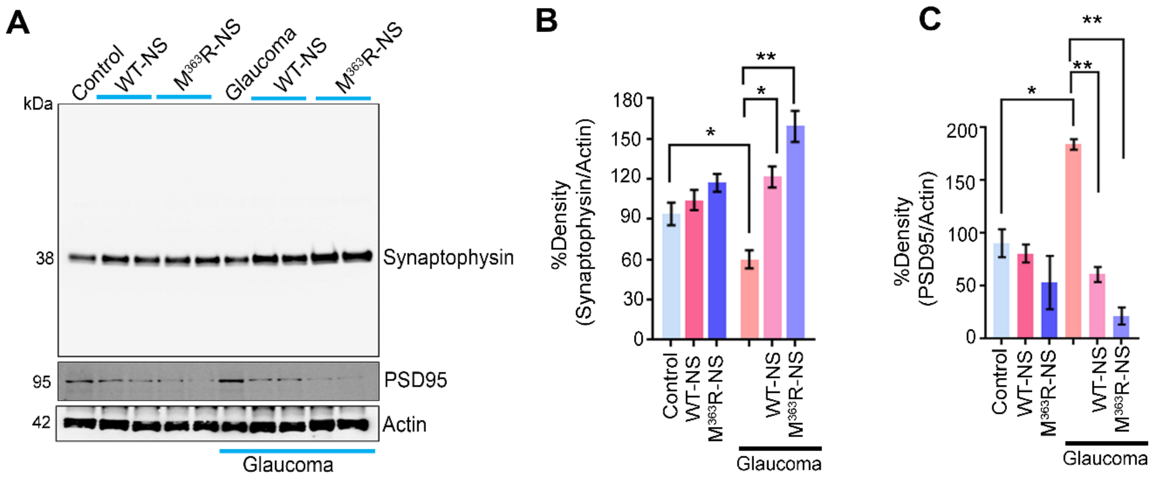

Figure S45 Pre- and post-synaptic marker changes in WT-NS and M^{363}R-NS administered mice (A) WB analysis of synaptophysin and PSD95 proteins in WT-NS and M^{363}R-NS administered mice in control and glaucoma conditions. Actin was used as a loading control. (B) In control, the immunoreactivity of synaptophysin showed no significant changes in retina lysates in either WT-NS or M363R-NS treated group. Induction of glaucoma caused a significant decrease in synaptophysin expression ($p<0.05$), however, WT ($p<0.02$) and M^{363}R-NS ($p<0.009$) treated group showed enhanced synaptophysin expression (C) Immunoreactivity of PSD95 showed a decrease in its levels in retina lysates from mice treated with WT and Mut-NS and induction of experimental glaucoma resulted in enhanced PSD95 levels ($p<0.02$). WT ($p<0.003$) and M^{363}R-NS ($p<0.002$) treated groups demonstrated reduced levels of PSD95 in glaucoma. n=3 animals/ group.

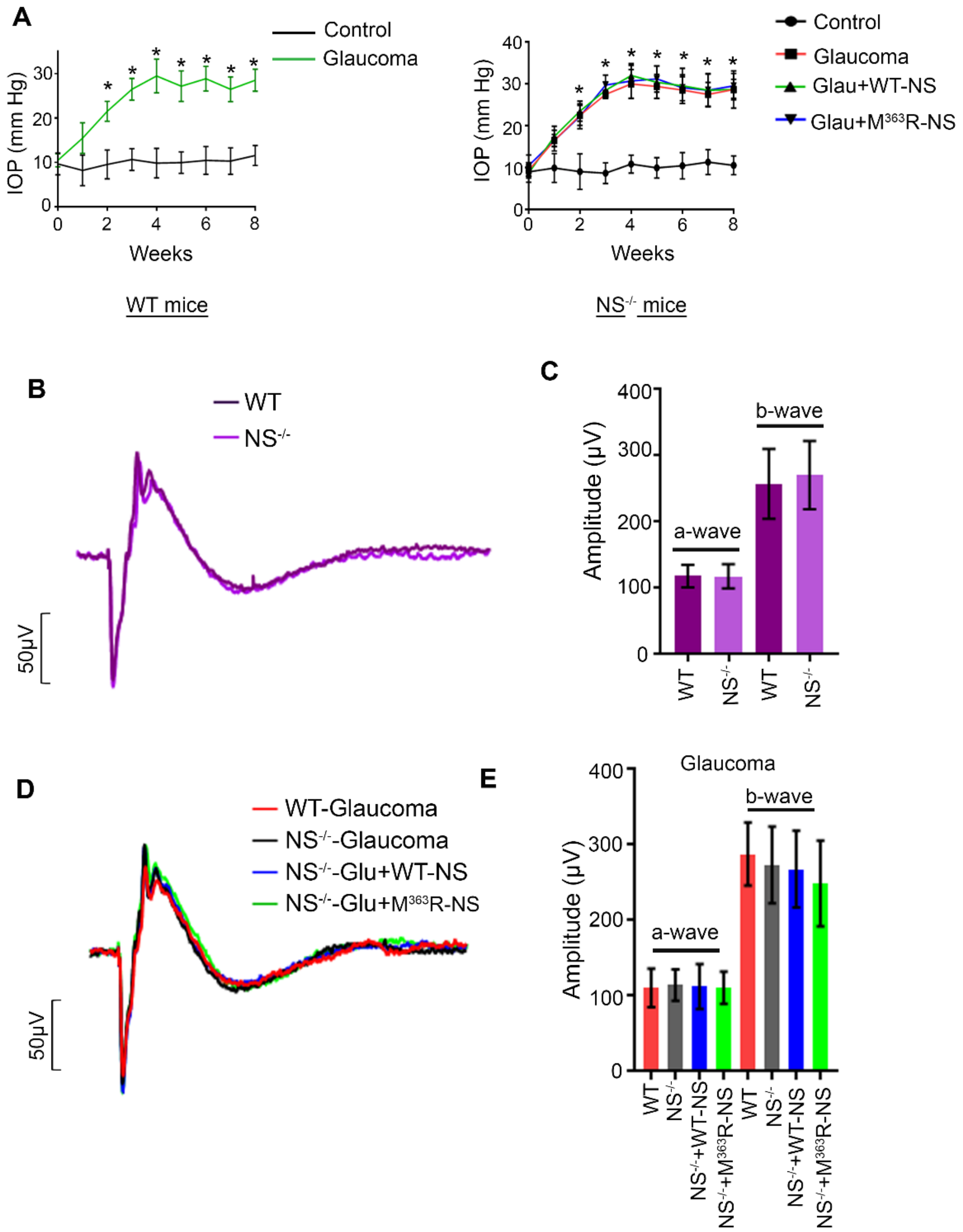

Figure S46 (A) WT and $NS^{-/-}$ control mice demonstrated a steady IOP level maintained at an average value of 10.10±2.3 and 9.94±3.3 mmHg, respectively. Weekly injections (8 weeks) of microbeads alone (27.76±2.3), microbeads+WT-NS (27.80±2.3 mmHg at eight weeks) and microbeads+M^{363}R-NS (28.39±2.4 mmHg at eight weeks) induced an elevation of IOP. (B) Average ERG traces of WT control and $NS^{-/-}$ mice at control IOP condition (C) Data analyses of ERG a- and b-wave amplitudes revealed no significant differences between the groups (WT mice control and $NS^{-/-}$ mice control) (D) Average ERG traces of WT (red), $NS^{-/-}$ (grey), $NS^{-/-}$ +WT-NS (blue) and $NS^{-/-}$+M^{363}R-NS (green) in experimental glaucoma conditions (E) Data analyses of ERG a- and b-wave amplitudes revealed no significant differences between groups (WT glaucoma, $NS^{-/-}$ glaucoma, $NS^{-/-}$ glaucoma+WT-NS and $NS^{-/-}$ glaucoma+M^{363}R-NS). n=10 in each group.

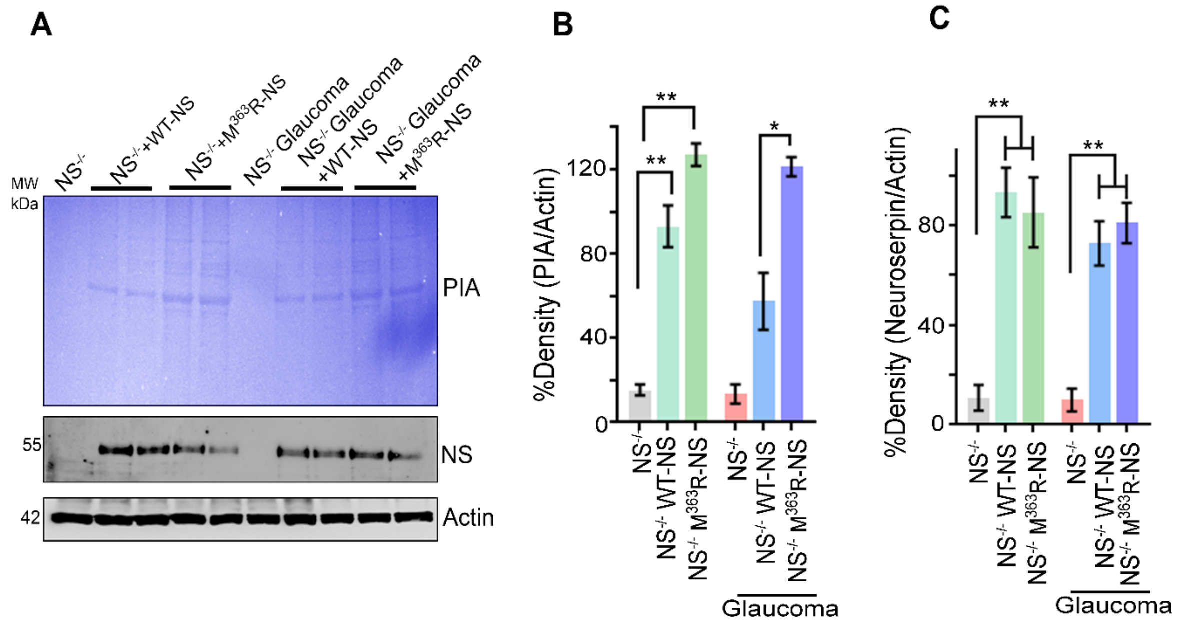

Figure S47 Plasmin inhibitory activity assessment in NS$^{-/-}$ mice retina treated with M^{363}R -NS protein in control and experimental glaucoma mice **(A)** Gelatin gel zymography indicating PIA from NS$^{-/-}$control, NS$^{-/-}$WT-NS, NS$^{-/-}$M^{363}R-NS in control and glaucoma conditions. Retina lysates were also subjected to WB and probed for neuroserpin immunoreactivity. Actin was used as a loading control **(B)** Relative band intensities were quantified, and data analysis indicated significantly higher plasmin inhibitory activity in both control and glaucoma conditions treated with WT-NS (control $p<0.008$; glaucoma $p<0.04$) and M^{363}R-NS in NS$^{-/-}$ mice (p<0.0.001). **(C)** Significantly higher levels of WT and M^{363}R- neuroserpin were detectable in NS$^{-/-}$ mice following M^{363}R-NS administration (WT control $p<0.009$; WT high IOP $p<0.01$; M^{363}R Control $p<0.01$; M^{363}R High IOP $p<0.008$). n=3 animals in each group.

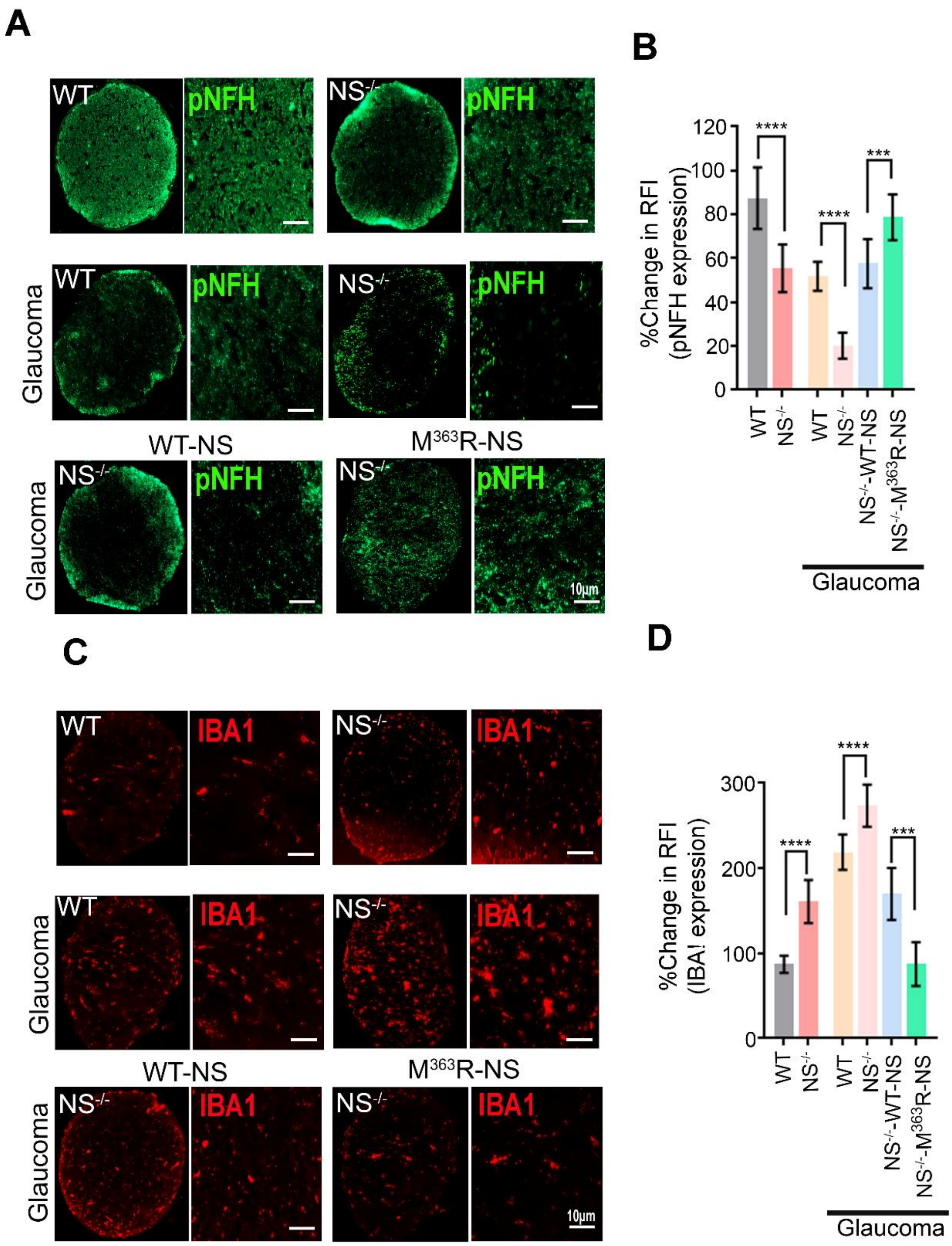

Figure S48 $M^{363}R$ modified neuroserpin intravitreal administration in the $NS^{-/-}$ mice rescues optic nerve structural deficits against glaucomatous damage. (A) Phosphorylated neurofilament heavy-chain (pNFH) immunoreactivity in control and glaucoma conditions treated with either WT-NS or $M^{363}R$-NS protein, photomicrographs showing pNFH immunostaining in transverse sections of the proximal portion of the optic nerve in normal and high IOP condition. Enlarged images are shown on the right-side panels. (B) Quantification of pNFH expression showed a significant decrease in NS-/- mice compared to WT mice ($p<0.0001$). Induction of glaucoma significantly reduced pNFH immunoreactivity in both WT and $NS^{-/-}$ mice, but a greater decline in pNFH expression was observed in $NS^{-/-}$ mice in experimental glaucoma ($p<0.0001$). Both WT-NS and $M^{363}R$-NS protein administration in $NS^{-/-}$ mice in high IOP conditions significantly

increased pNFH expression ($p<0.0001$), and the increase was significantly more in the case of mice administered $M^{363}R$-NS compared to the WT form ($p<0.0007$). (C) Photomicrographs showing ionized calcium-binding adaptor molecule 1 (IBA1) immunostaining patterns in cross-sections of WT and $NS^{-/-}$ mice optic nerves in control and glaucoma conditions injected with either WT-NS or $M^{363}R$-NS protein. Enlarged images are shown on the right (D) Quantification of IBA1 levels revealed a significant increase in IBA1 reactivity in $NS^{-/-}$ compared to WT mice ($p<0.0001$). Induction of glaucoma significantly increased IBA1 immunoreactivity in both WT and $NS^{-/-}$ mice, but a greater increase was observed in $NS^{-/-}$ mice ($p<0.002$). WT-NS and $M^{363}R$-NS protein overexpression in $NS^{-/-}$ mice in high IOP conditions resulted in a significant decrease in IBA1 expression ($p<0.0001$), and the decrease was more in the group administered $M^{363}R$ modified NS administered protein ($p<0.0005$). Scale bar = 10μm. n=4 animals in each group.

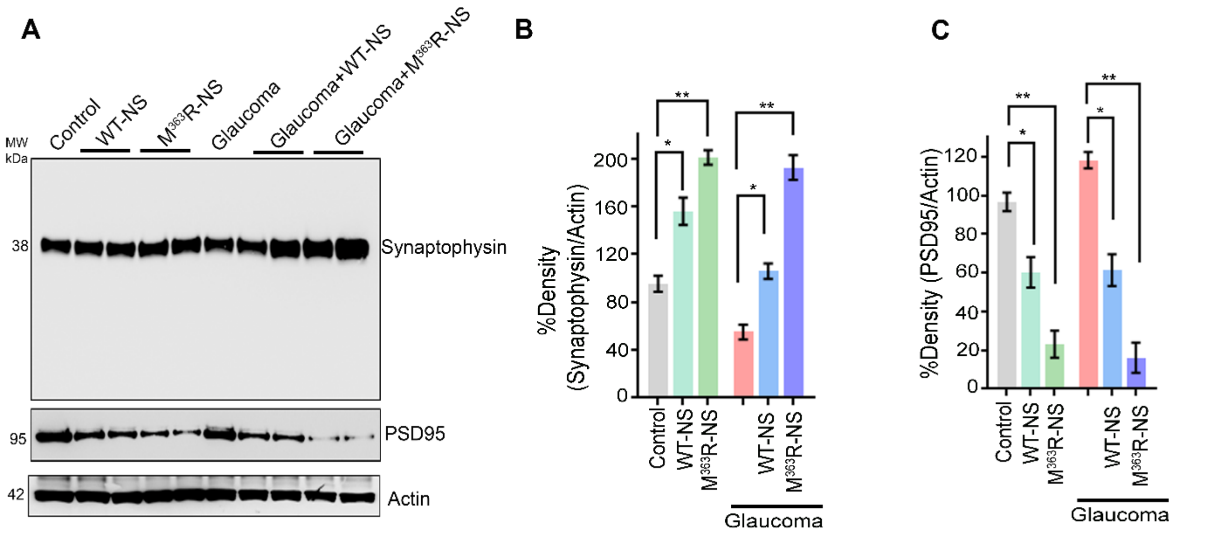

Figure S49 Changes in Pre- and post-synaptic markers in the $NS^{-/-}$ mice administered WT-NS or M^{363}R-NS protein in control and high IOP conditions (A) Expression levels of synaptophysin and PSD95 protein markers in retina lysates from $NS^{-/-}$ mice, $NS^{-/-}$ WT-NS, $NS^{-/-}$ M^{363}R-NS, $NS^{-/-}$ glaucoma, $NS^{-/-}$ glaucoma+WT-NS and $NS^{-/-}$ glaucoma+ M^{363}R-NS were evaluated. Actin was used as a loading control. (B) Densitometric evaluation of synaptophysin showed a significant increase in WT-NS ($p<0.02$) and M^{363}R-NS protein ($p<0.003$) administered in mice compared to control. Induction of experimental glaucoma resulted in a significant decline in synaptophysin immunoreactivity ($p<0.02$). WT ($p<0.01$) and M^{363}R-NS ($p<0.003$) administration in glaucoma enhanced synaptophysin expression, and the increase was significantly more in the M^{363}R-NS administered group compared to the WT NS administered animals ($p<0.009$) (C) Immunoreactivity of PSD95 showed a significant decrease in retinas treated with WT-NS and M^{363}R-NS compared to $NS^{-/-}$ control mice ($p<0.03$). Induction of experimental glaucoma induced a significant increase in PSD95 immunoreactivity in $NS^{-/-}$ mice retinas ($p<0.04$). Administration of WT ($p<0.01$) and M^{363}R-NS (0.003) in glaucoma significantly downregulated PSD95 expression, and the decrease was significantly more in the case of the M^{363}R-NS administered group compared to the WT NS administered animals ($p<0.02$). n=3 animals in each group.

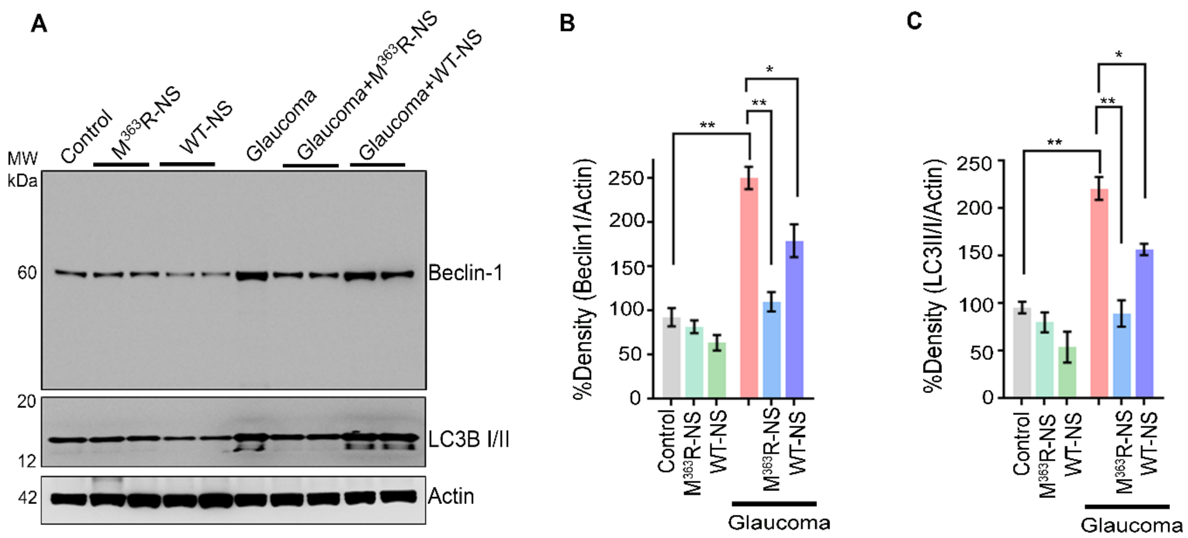

Figure S50 Altered autophagy markers in $NS^{-/-}$ mice in response to WT-NS and M^{363}R-NS intravitreal administration in control and high IOP condition (A) WB analysis of Beclin-1 and LC3B-II/LC3B-I levels in retina lysates of $NS^{-/-}$ mice in control and high IOP conditions with either WT-NS or M^{363}R-NS administration. β-actin immunoreactivity was measured as an endogenous control. (B) Densitometric Quantification of Beclin-1 immunoreactivity showed no significant changes in control IOP conditions. Induction of experimental glaucoma induced significant elevation of Beclin-1 immunoreactivity compared to control ($p<0.005$). The administration of M^{363}R-NS ($p<0.007$) or WT NS ($p<0.04$) proteins in $NS^{-/-}$ in glaucoma significantly downregulated Beclin-1 expression, and a greater decline of Beclin-1 expression was evident in $NS^{-/-}$ mice in M^{363}R-NS administered group ($p<0.04$). (C) Immunoreactivity of an LC3B-II/LC3B-I ratio showed no significant changes in control IOP conditions. Induction of experimental glaucoma induced significant elevation of LC3B-II/LC3B-I ratio compared to control ($p<0.005$). The administration of M^{363}R-NS ($p<0.009$) or WT NS ($p<0.02$) protein in $NS^{-/-}$ mice in glaucoma significantly downregulated the LC3B-II/LC3B-I ratio and greater decline in LC3B-II/LC3B-I ratio was evident in $NS^{-/-}$ mice administered M^{363}R-NS compared to WT-NS treated group ($p<0.02$). n=3 animals / group.

Table

Table S1: The following tables include all the primary and secondary antibodies use in western blot and immunofluorescence analysis.

Company	Primary antibody	Host	Application
**Santa Cruz**	Anti-Neuroserpin (sc48360)	Mouse	Intravitreal injection
	Anti-IgG (sc2025)	Mouse	Intravitreal injection
**Abcam, UK**	Anti-neuroserpin (ab33077)	Rabbit	Western blot: 1:1000 dilution Immunofluorescence: 1:300 dilution
	Anti-GFP (ab1218)	Mouse	Western blot: 1:1000 dilution Immunofluorescence: 1:300 dilution
	Anti-synaptophysin (ab32127)	Rabbit	Western blot: 1:2000 dilution Immunofluorescence: 1:300 dilution
	Anti-β III tubulin (ab7751)<br>Anti-β III tubulin (ab215037)	Mouse<br>Rabbit	Immunofluorescence: 1:300 dilution
	Anti-NeuN (ab104224)<br>Anti-NeuN (ab104225)	Mouse<br>Rabbit	Immunofluorescence: 1:300 dilution
	Anti-β actin (ab6276)	Mouse	Western blot: 1:10000 dilution
**Cell Signaling Technology, USA**	Anti-Beclin1 (3495)	Rabbit	Western blot: 1:1000 dilution
	Anti-LC3A/B (12741)	Rabbit	Western blot: 1:1000 dilution
**Novachem, Australia**	Anti-Iba1 (019-19741)	Rabbit	Immunofluorescence: 1:300 dilution
**BioLegend, USA**	Anti-pNFH (801601)	Mouse	Immunofluorescence: 1:300 dilution
**ThermoFisher Scientific, USA**	Anti-PSD95 (516900)	Rabbit	Western blot: 1:1000 dilution
**Merck, Germany**	Anti-Brn3a (MAB1585)	Mouse	Immunofluorescence: 1:300 dilution

Company	Secondary antibody
**R&D Systems, USA (For Western blot)**	Anti-Rabbit HRP secondary antibody (HAF008) (1:2500 dilution)
	Anti-Mouse HRP secondary antibody (HAF018) (1:2500 dilution)
**Jackson ImmunoResearch Laboratories, USA (For immunofluorescence)**	Cy3 AffiniPure Donkey Anti-Rabbit IgG (H+L) (711-165-152) (1:300 dilution)
	Alexa Fluor 488 AffiniPure Donkey Anti-Mouse IgG (H+L) (715-545-150) (1:300 dilution)
